# Early-Stage Alcoholic Cardiomyopathy Highlighted by Metabolic Remodeling, Oxidative Stress, and Cardiac Myosin Dysfunction in Male Rats

**DOI:** 10.3390/ijms26146766

**Published:** 2025-07-15

**Authors:** David V. Rasicci, Jinghua Ge, Adrien P. Chen, Neil B. Wood, Skylar M. L. Bodt, Allyson L. Toro, Alexandra Evans, Omid Golestanian, Md Shahrier Amin, Anne Pruznak, Nelli Mnatsakanyan, Yuval Silberman, Michael D. Dennis, Michael J. Previs, Charles H. Lang, Christopher M. Yengo

**Affiliations:** 1 Department of Cell and Biological Systems, Penn State College of Medicine, Hershey, PA 17033, USA; david.rasicci@hsc.wvu.edu (D.V.R.); jinghuage@pennstatehealth.psu.edu (J.G.); apc6225@psu.edu (A.P.C.); sbodt@pennstatehealth.psu.edu (S.M.L.B.); atoro@pennstatehealth.psu.edu (A.L.T.); ampruznak@psu.edu (A.P.); nmnatsakanyan@pennstatehealth.psu.edu (N.M.); mdennis@pennstatehealth.psu.edu (M.D.D.); clang@pennstatehealth.psu.edu (C.H.L.); 2 Department of Pathology, Anatomy, and Laboratory Medicine, West Virginia University School of Medicine, Morgantown, WV 26505, USA; omid.golestanian@hsc.wvu.edu (O.G.); amin.mdshahrier@mayo.edu (M.S.A.); 3 Department of Molecular Physiology and Biophysics, University of Vermont, Burlington, VT 05405, USA; nwood11@jhu.edu (N.B.W.); michael.previs@med.uvm.edu (M.J.P.); 4 Department of Neuroscience and Experimental Therapeutics, Penn State College of Medicine, Hershey, PA 17033, USA; aevans16@pennstatehealth.psu.edu (A.E.); ysilberman@pennstatehealth.psu.edu (Y.S.)

**Keywords:** myosin, actin, muscle contraction, mitochondria, heart failure, alcohol

## Abstract

Chronic ethanol use can lead to alcoholic cardiomyopathy (ACM), while the impact on the molecular and cellular aspects of the myocardium is unclear. Accordingly, male Sprague-Dawley rats were exposed to an ethanol-containing diet for 16 weeks and compared with a control group that was fed an isocaloric diet. Histological measurements from H&E slides revealed no significant differences in cell size. A proteomic approach revealed that alcohol exposure leads to enhanced mitochondrial lipid metabolism, and electron microscopy revealed impairments in mitochondrial morphology/density. Cardiac myosin purified from the hearts of ethanol-exposed animals demonstrated a 15% reduction in high-salt ATPase activity, with no significant changes in the in vitro motility and low-salt ATPase or formation of the super-relaxed (SRX) state. A protein carbonyl assay indicated a 20% increase in carbonyl incorporation, suggesting that alcohol may impact cardiac myosin through oxidative stress mechanisms. In vitro oxidation of healthy cardiac myosin revealed a dramatic decline in ATPase activity and in vitro motility, demonstrating a link between myosin protein oxidation and myosin mechanochemistry. Collectively, this study suggests alcohol-induced metabolic remodeling may be the initial insult that eventually leads to defects in the contractile machinery in the myocardium of ACM hearts.

## 1. Introduction

Alcohol (or ethanol) is a pervasive substance that transcends culture internationally. As the most consumed drug in human history, ethanol presents a global burden of disease, responsible for roughly 6% of all deaths (3.3 million/year) [1,2]. The detrimental impact of chronic ethanol consumption on human biology is well known in several organ systems, most notably the liver and central nervous system. However, detrimental impact on the cardiovascular system also has been reported, as excessive consumption has been associated with hypertension, stroke, obesity, and decreased systolic function [3]. Specifically, prolonged excessive ethanol consumption (>80 g of ethanol/day for >5 years) may lead to alcoholic cardiomyopathy (ACM), which can be defined as a dilated cardiomyopathy (DCM) of toxic origin with low left ventricular ejection fraction (<50%) and chamber dilatation (>2 SD) with potential progression to congestive heart failure [4]. ACM occurs in the absence of hypertension, valvular, or ischemic disease and is responsible for roughly 1/3 of all non-ischemic DCM cases, with a high mortality rate without a proactive attempt to achieve and maintain abstinence. Notably, men are at a much higher risk than women (9:1) [5].

Previous work has led to an understanding that ACM pathogenesis is multi-factorial. The oxidative nature of ethanol and its metabolites has been shown to induce myocytolysis, apoptosis, and necrosis, with compensatory hypertrophy, fibrosis, and activation of the renin–angiotensin system [5]. Moreover, ethanol consumption induces changes in cardiomyocyte membrane composition (e.g., phospholipids, ion channels, receptors) and physiology, including intracellular calcium transients [4]. Previous work also has demonstrated that protein balance is compromised in ACM, with increased protein degradation and loss of protein mass due to decreased global protein synthesis [6,7]. Metabolic and mitochondrial impairments also have been documented with changes in cardiomyocyte fatty acid uptake [8] and respiratory complex activities (e.g., decreases in Complexes I, II, and IV in the ETC) [9,10] as well as swollen megamitochondria and distortion of inner membrane cristae in the face of chronic ethanol consumption [10].

**Figure 1:**
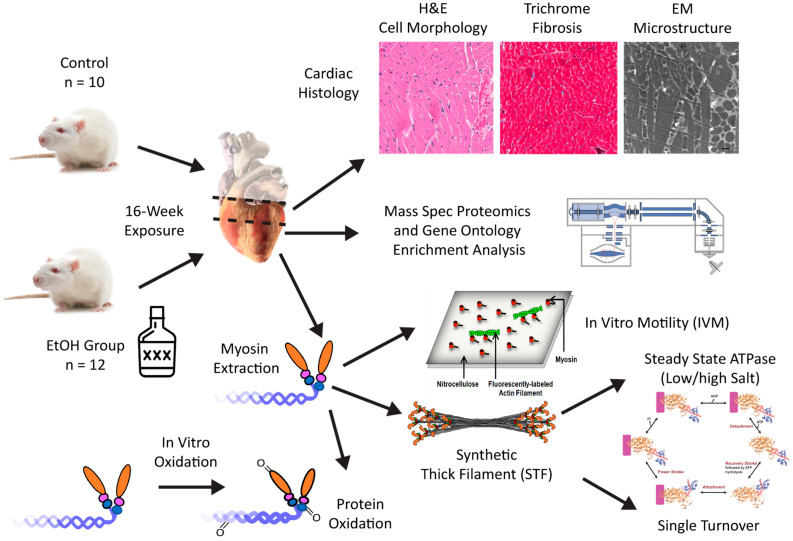
Workflow overview. A schematic representation of the project workflow is illustrated highlighting the key experimental steps: heart dissections from control and ethanol-consuming (EtOH) groups followed by cardiac histological analysis, mass spectrometry, myosin extraction, in vitro motility, assembly of synthetic thick filaments, measurements of steady-state ATPase activity and single ATP turnover rate, and protein oxidation experiments.

At an organ level, it is well understood that the dilated ACM heart exhibits decreased contractility. What underlies this decrease in systolic function has yet to be fully resolved. Given the multifaceted nature of the disease, it has been predicted that one (or more) of several factors may contribute to reduced contractility, including tissue level changes (e.g., fibrosis or elastic changes) or intracellular changes such as disruptions in calcium handling and excitation–contraction coupling, myofibrillary structure, decrease in overall cellular protein balance, mitochondrial dysfunction, and/or other metabolic changes such as decreased ATP/ADP ratios [4,5,10,11]. Few previous studies have focused specifically on the motor protein myosin, whose molecular interactions with actin filaments directly drive cardiac contractility. Accordingly, the primary aim of this study was to characterize the mechanochemical properties of myosin (e.g., in vitro motility, ATPase activity, and auto-inhibition/super relaxed state) following 16 weeks of ethanol consumption in adult male Sprague-Dawley rats. The second aim of the study was to characterize the ACM phenotype through histology, electron microscopy, mass spectrometry, and measures of protein oxidation. This approach examined the interplay between metabolic and contractile dysfunction, hypothesized to be a crucial aspect of ACM. For example, if mitochondrial dysfunction is the initial target of chronic alcohol consumption, enhanced reactive oxygen species may lead to oxidative damage of the contractile machinery. Thus, metabolism was assessed through proteomic analysis and mitochondrial morphology and matrix/density via electron microscopy. Myosin oxidation levels were measured directly in the alcohol-treated animals and were supplemented with in vitro oxidation experiments of healthy cardiac myosin in order to establish the causal effect of oxidation on myosin mechanochemistry. An overview of the experimental design and workflow is illustrated in Figure 1. Overall, the study was designed to provide insight into the molecular/cellular impairments that lead to reduced organ-level contractility in the ACM disease phenotype.

## 2. Results

### 2.1. Animal Morphometrics

For overall body mass, EtOH animals had decreased body mass compared with controls (EtOH = 300 ± 23.1 g vs. CTL = 325 ± 19.5 g; *p* = 0.0163). For heart mass, there was no statistical difference between the groups (EtOH = 834 ± 76 mg vs. CTL = 883 ± 54 mg; *p* = 0.1031). HW–BW ratios also were calculated, in which there was no significant difference between groups (EtOH = 2.77 ± 0.11 mg/g; CTL = 2.72 ± 0.09 mg/g; *p* = 0.2635). As a snapshot of blood ethanol levels at the time of euthanasia, serum was used to measure BAC. There was a clear difference between the groups (EtOH = 35.3 ± 25.5 mg/g; CTL = 1.93 ± 0.99 mg/g; *p* = 0.0005), confirming the impact of ethanol consumption (Supplementary Figure S1A). There was some variability in the BAC levels of the EtOH animals, which may be caused by the variability in ethanol intake on the day of euthanasia. The BAC measurements have a run variance of 2.5%, and thus, some of the control animals that did not receive any alcohol reported very low/non-zero BAC levels. We also tracked the dietary consumption in the alcohol group and found that there was some variability from animal to animal in the average daily consumption (Supplementary Figure S1B). The animal morphometrics are summarized in Table 1.

### 2.2. Routine Histology and Electron Microscopy

**Figure 2:**
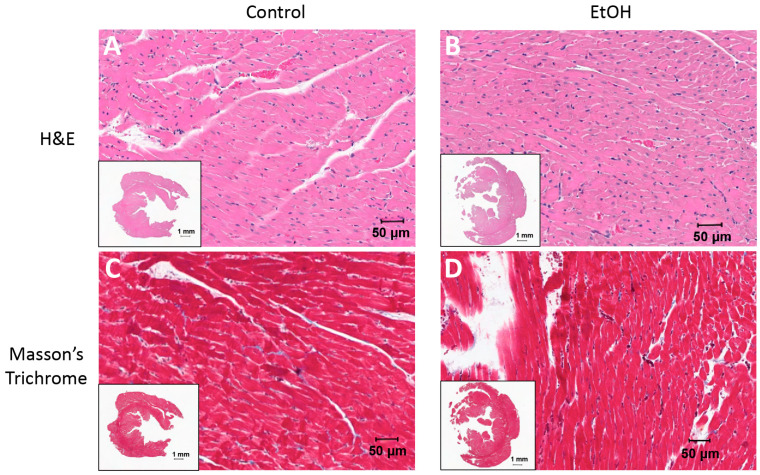
Histological images. Representative images of hematoxylin and eosin (H&E, **A**,**B**) and Masson’s Trichrome (**C**,**D**) staining from the control (**A**,**C**) and EtOH (**B**,**D**) groups at 20× magnification (insets at 1×).

Routine histology (H&E + Masson’s trichrome) and electron microscopy both were utilized to assess potential structural changes in response to ethanol consumption (CTL: *n* = 10, EtOH: *n* = 12). In H&E measures, cell diameter was measured in cross-section, which did not demonstrate a significant change in size (Table 2). There were no changes in H&E measures of cell diameter in the coronal plane (EtOH = 15.67 ± 1.53 µm vs. CTL = 15.42 ± 2.44 µm; *p* = 0.7761). Also, there were no detectable changes in fibrosis measured by Masson’s trichrome (Figure 2).

**Figure 3:**
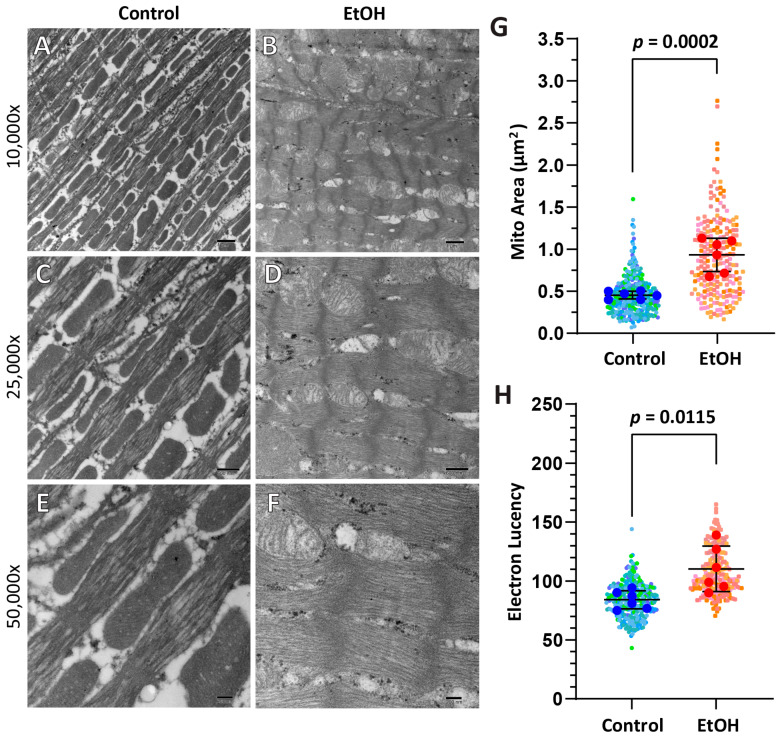
Electron microscopy (EM) images. Representative EM images highlight the ultrastructural differences observed between the control and EtOH groups presented at three different magnifications: 10,000× (**A**,**B**), 25,000× (**C**,**D**), and 50,000× (**E**,**F**). Quantitative analysis of mitochondrial area (**G**) and electron lucency (**H**) is illustrated using a SuperPlot, where color-coded transparent dots represent individual mitochondrial measurements collected from different animals. Each solid dot indicates the mean for each animal (*n* = 6 per group). The black line denotes the group mean ± SD. Statistical significance was evaluated using an unpaired *t*-test. Corresponding numerical data and *p*-values are provided in Table 2.

A subset of animals (*n* = 6) from each group was included in the ultrastructural EM analysis to examine sarcomere and mitochondrial structure. For sarcomere length (*n* = 6), there were no changes between groups (EtOH = 1659 ± 377 nm vs. CTL = 1918 ± 222 nm; *p* = 0.164), though there was notably higher variation in the EtOH group. Qualitatively, some EtOH-exposed animals demonstrated clear mitochondrial abnormalities (swelling, increased overall density, disrupted cristae) (Figure 3A–F). Mitochondrial abnormalities were supported quantitatively in the same subset of animals (*n* = 6), which demonstrated a change in mitochondrial area (EtOH = 0.93 ± 0.20 μm^2^ vs. CTL 0.45 ± 0.05 μm^2^; *p* = 0.0002) as well as swelling level, quantified by electron lucency (EtOH = 110.40 ± 19.32 AU vs. CTL = 84.18 ± 7.68 AU; *p* = 0.0115) (Figure 3G,H).

### 2.3. Proteomics via Mass Spectrometry

**Figure 4:**
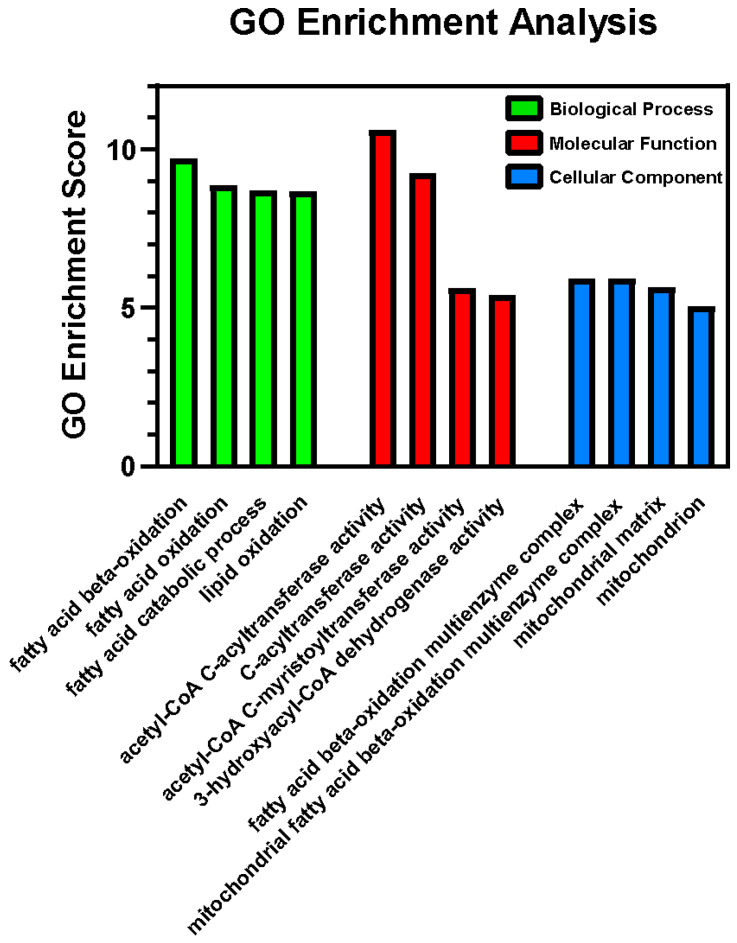
Gene Ontology (GO) Enrichment Analysis. GO enrichment analysis filtered through three primary categories: biological process (green), molecular function (red), and cellular component (blue). Results highlight that differentially expressed proteins are involved in fatty acid β-oxidation through specific multienzyme complexes within the mitochondria.

Proteins were digested with trypsin to produce peptides and analyzed by mass spectrometry. Relative protein abundances were determined by label-free quantification (LFQ). Proteins that met the threshold for differential expression are presented in Table 3, which includes gene name, protein accession number, fold change, and *p*-value of associated change. Accession numbers were input into GO Enrichment Analysis, which demonstrated an overwhelming enrichment in biological processes related to fatty acid β-oxidation, which was further supported via molecular function analysis (β-oxidation enzyme activity) and cellular component analysis (localized to fatty acid β-oxidation multienzyme complex and mitochondrial matrix) (Figure 4). The significant differences in proteins that were increased or decreased in the abundance in the EtOH group were assessed with a volcano plot (Supplemental Figure S2) with the threshold set at 1.2-fold differences and a *p*-value < 0.01. The results suggest an increase in fatty acid metabolism, with enhanced β-oxidation, perhaps triggered by an increase in fatty acid accumulation in cardiomyocytes. Other notable changes were altered levels of sarcomeric proteins, suggestive of remodeling of the contractile machinery, and proteins of the mitochondria matrix, indicative of mitochondria damage. For completion, all proteins detected by mass spectrometry analysis are reported in Supplementary Table S2.

### 2.4. Mechanical Properties—In Vitro Motility

**Figure 5:**
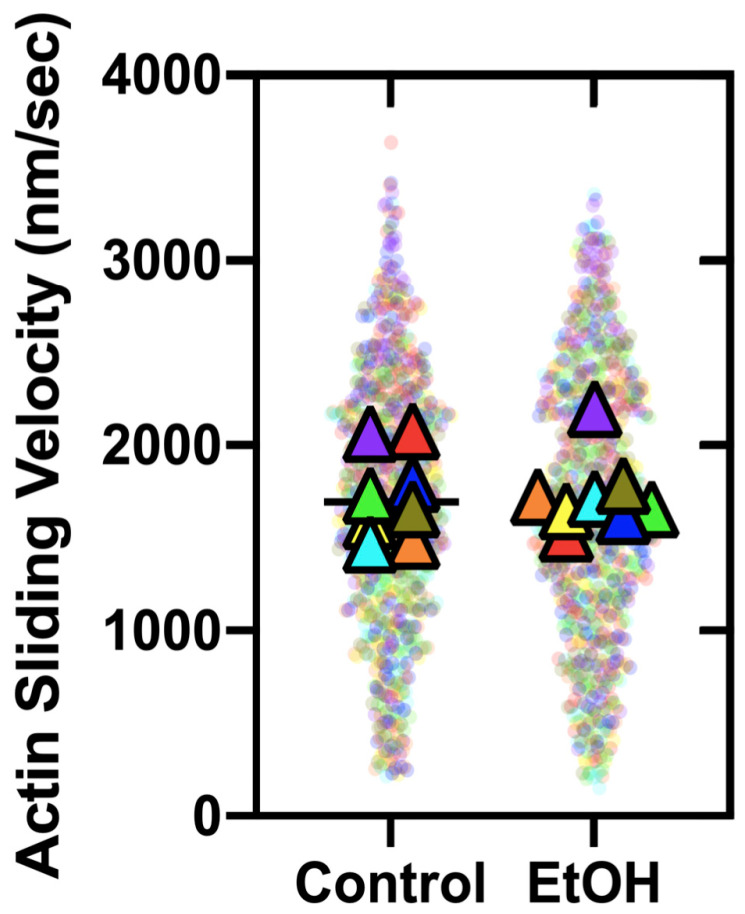
In vitro motility assay. Actin sliding velocities from the unloaded in vitro motility assay are represented as a SuperPlot. Each triangle corresponds to an individual experiment (sample mean) from separate myosin preparations (*n* = 8). The color-coded transparent dots illustrate the individual velocities associated with each triangle, while the black line represents the mean of the sample means.

The motor function of isolated full-length myosin molecules was assessed in unloaded in vitro motility (IVM) assays using a subset (*n* = 8) of animals from each group. No differences were detected between groups (EtOH = 1732 ± 203 nm/s vs. CTL = 1732 ± 235 nm/s; *p* = 1.000). Similarly, no differences were detected in the percentage of stuck filaments, an indication of damaged motors plated on the cover slip (EtOH = 13.4 ± 4.3% vs. CTL = 13.3 ± 6.7%; *p* = 0.9902) (Figure 5, Table 4). Thus, cardiac myosin extracted from respective groups exhibited similar behavior in the unloaded IVM assay.

### 2.5. Synthetic Thick Filament (STF) Formation

Using extracted full-length myosin (Supplemental Figure S3A), STFs were reconstituted through reduction in salt concentration as previously described [12]. SDS gels were utilized to assess the ability of myosin molecules to reassemble into thick filaments. Per densitometric analysis of SDS gels (Supplemental Figure S3B), myosin assembled into thick filaments, which precipitated and were pelleted by centrifugation. No detectable myosin was observed in the supernatant for either the control or ethanol groups.

### 2.6. Biochemical Properties—ATPase and Single Turnover

**Figure 6:**
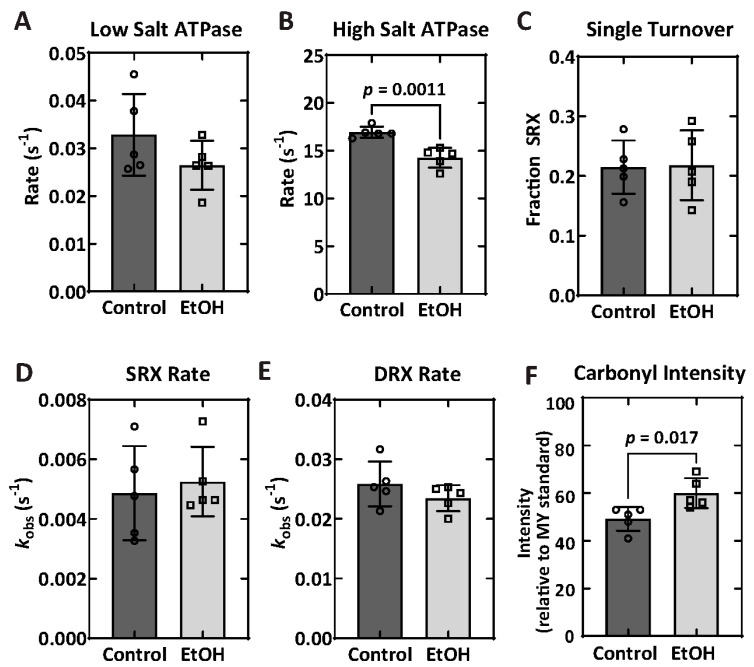
Mechanochemical characterization. Biochemical analyses of myosin properties in the control and EtOH groups, including low-salt ATPase activity (**A**), high-salt ATPase activity (**B**), fraction of the super-relaxed state (SRX) determined by single ATP turnover (**C**), SRX rates (**D**), DRX rates (**E**), and carbonyl intensity (**F**). Data are presented as mean ± SD (*n* = 5 per group). Statistical significance was evaluated using an unpaired *t*-test.

STFs were used to assess the biochemical properties of myosin, including enzymatic characterization of the motor (ATPase assays). A subset of animals (*n* = 5 for each group) were utilized in these assays. Under low-salt conditions in which the autoinhibited state of myosin can form, there was no difference between the groups (EtOH = 0.027 ± 0.005 s^−1^ vs. CTL = 0.033 ± 0.009 s^−1^; *p* = 0.1906). However, in the high-salt ATPase assay, which is extremely sensitive to the number of active myosin heads, there was a significant reduction in the enzymatic capacity of the STFs formed from extracted myosin from the ethanol group (EtOH = 14.3 ± 1.1 s^−1^ vs. CTL = 16.9 ± 0.6 s^−1^; *p* = 0.0011) (Figure 6A,B; Table 4).

The ATPase kinetics were further examined by performing single ATP turnover assays, which provide information about the fraction of myosin in the autoinhibited SRX and uninhibited disordered relaxed (DRX) state. In the low-salt conditions, there were no differences in SRX fraction (EtOH = 0.22 ± 0.06 vs. CTL = 0.22 ± 0.04; *p* = 1.000), SRX rate (EtOH = 0.0052 ± 0.0012 s^−1^ vs. CTL = 0.0049 ± 0.0016 s^−1^; *p* = 0.7549), or DRX rate (EtOH = 0.023 ± 0.002 s^−1^ vs. CTL = 0.026 ± 0.004 s^−1^; *p* = 0.1720) between groups (Figure 6C–E; Table 4). Representative single turnover traces are demonstrated in Supplemental Figure S4.

### 2.7. Protein Carbonylation

We investigated the degree of oxidative damage to myosin by performing a carbonylation assay. Carbonylation was examined in the extracted myosin via Western blot and normalized to protein concentration for a subset of animals (*n* = 5 per group). In this assay, there was a significant increase in the ratio of carbonylation in the EtOH group (EtOH = 60.0 ± 6.3% vs. CTL = 49.2 ± 5.0%; *p* = 0.017), roughly a 20% increase in the carbonylation of animals exposed to ethanol (Figure 6F; Table 4). Representative gels are included in Supplemental Figure S5.

### 2.8. In Vitro Oxidation Treatment of Myosin

**Figure 7:**
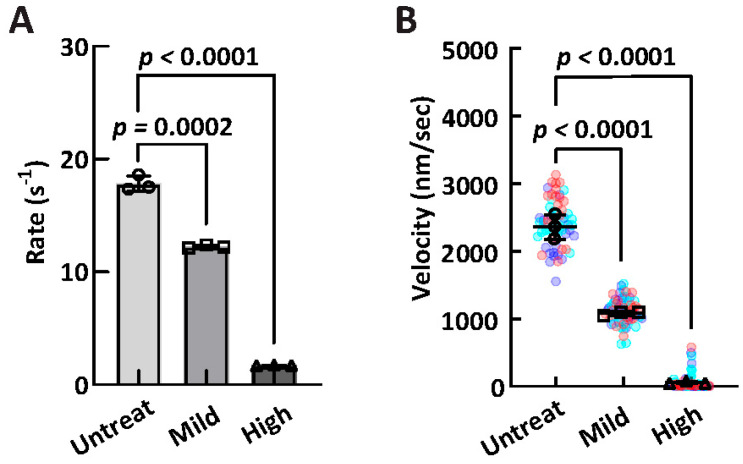
In vitro oxidation assay. Comparison of high-salt ATPase activity (**A**) and in vitro motility (**B**) across three conditions: untreated, mild oxidative treatment (1 mm H_2_O_2_), and high oxidative treatment (100 mM H_2_O_2_, 10 μM FeCl_3_, 1 mM ascorbic acid). Data are presented as mean ± SD (*n* = 3 per group). Statistical significance was evaluated using an unpaired *t*-test.

To assess the relationship between oxidation and myosin mechanochemical function, healthy rat cardiac myosin was extracted and treated in vivo with stepwise treatment (untreated, mild 1 mM H_2_O_2_, high at 100 mM H_2_O_2_ + 1 uM FeCl_3_ + 1 mM ascorbic acid), and then subsequently tested in ATPase and IVM assays for a subset of animals (*n* = 3). In the high-salt ATPase assay, a stepwise decrease in enzymatic capacity was seen from untreated myosin (17.83 ± 0.69 s^−1^) to mild treatment (12.24 ± 0.12 s^−1^) to high treatment (1.71 ± 0.03 s^−1^). Similarly, in the IVM assay, a stepwise decrease in ensemble motor function was observed from untreated myosin (2414 ± 185 nm/s) to mild treatment (1106 ± 31 nm/s) to high treatment (60 ± 16 nm/s). Results are reported in Figure 7 and Table 5.

### 2.9. mRNA Expression

To evaluate changes in antioxidant enzymes and pro-inflammatory factors, gene expression was quantified in heart tissue lysates by PCR analysis. In the hearts of animals exposed to ethanol, expression of both interferon alpha and interferon beta were reduced (Supplemental Figure S6A,B). Moreover, there was a trend toward upregulation of catalase (CAT, *p* = 0.0518) and tumor necrosis factor alpha (TNF⍺, *p* = 0.0662) in the hearts of mice exposed to ethanol (Supplemental Figure S6C,D). However, ethanol exposure did not alter cardiac expression of several other pro-inflammatory factors (REDD1, CCL2, CCL5, ICAM-1, IL6) or antioxidant enzymes (NQ01, HO-1, GPX1, SOD1, SOD2) (Supplemental Figure S7).

## 3. Discussion

Chronic ethanol consumption can lead to alcoholic cardiomyopathy (ACM), in which the heart assumes a dilated cardiomyopathy (DCM) phenotype with thin, weakened ventricular walls, enlarged chambers, and compromised systolic function [4,5]. It has been established that ACM pathogenesis is multi-factorial; however, few studies have focused specifically on the mechanochemical properties of myosin secondary to ethanol exposure. Accordingly, Sprague-Dawley rats were used to test the impact of ethanol consumption on cardiac structure, mitochondria morphology, mechanochemical properties of cardiac myosin, protein abundance via mass spectrometry, and the role of oxidation (Figure 1). Collectively, these results demonstrate that the 16-week exposure in rats resulted in significant metabolic remodeling, specifically upregulated mitochondrial lipid metabolism, which precedes major structural and contractile remodeling of the heart. Mitochondria were swollen with reduced matrix density and disrupted cristae morphology, providing further evidence of mitochondria dysfunction. Moreover, changes in myosin mechanochemistry are directly linked to protein oxidation. Collectively, these data imply that ethanol administration of 16 weeks captures an early stage of ACM pathogenesis, providing evidence that metabolic remodeling precedes contractile remodeling.

### 3.1. Morphometrics and Cardiac Structure

Despite an isocaloric diet, the ethanol-fed animals had a decreased body weight following the 16-week exposure. Chronic ethanol consumption has been associated with malnutrition and poor nutritional intake, metabolic changes, gastrointestinal inflammation, muscle atrophy, neurohormonal changes, and stress [13]. BAC measures confirmed an effective administration in which all EtOH animals regularly consumed ethanol (Supplemental Figure S1; Table 1). Notably, these BACs represent a minimum value of alcohol consumption at a single time point. Peak BACs would be expected in the early part of the dark cycle when liquid diet consumption is maximal.

Routine histologic examination demonstrated no changes in cell size and fibrosis (Figure 2; Table 2), inconsistent with the clinical phenotype of ACM with cellular hypertrophy and the initial hypothesis of the study. However, at an ultrastructural level of electron microscopy, there were clear signs of pathology, with altered mitochondrial size and density in some (but not all) animals (Figure 3). Some variability within the alcohol group was observed, with some animals demonstrating clear pathology, swollen megamitochondria with reduced matrix density and disrupted cristae, as previously reported in ACM [10,11]. Mitochondrial swelling occurs under certain pathological conditions due to increased calcium ion and water influx into the mitochondrial matrix [14]. As a result, the activation of the mitochondrial permeability transition pore (mPTP) occurs, leading to unfolding of mitochondrial cristae and a reduction in matrix density, which can be quantified by electron lucency measurements of EM images [15]. The prolonged activation of mPTP induces mitochondrial inner membrane depolarization and cellular ATP depletion, which could lead to detrimental consequences in the heart, leading to contractile dysfunction and initiation of apoptotic or necrotic cell death [16]. Other animals, however, did not have observable changes. Collectively, the changes at the ultrastructural level suggest this 16-week exposure marks an early stage of ACM pathogenesis. Mitochondrial changes were supported by the proteomic analysis, suggesting that the organelle is an early target of ethanol toxicity and that mitochondrial dysfunction could be the underlying cause of organ failure in the later stages of ACM.

### 3.2. Proteomics and GO Enrichment Analysis

Proteomics data demonstrated ethanol-induced changes in proteins involved in metabolic pathways and cytoskeletal structure. Differentially expressed proteins (DEPs) were studied individually (Table 3), and then were examined through Gene Ontology (GO) Enrichment Analysis through three primary filters: molecular function, biological process, and cellular component (Figure 4). Most commonly, the upregulated DEPs were localized to the mitochondria and related to metabolic processes (e.g., β-oxidation, the citric acid cycle, and the electron transport chain), which was consistent with mitochondrial abnormalities observed via electron microscopy. The proteomic results presented in this study demonstrate a window into possible changes in mitochondrial metabolism in ACM, and future studies that can perform direct metabolic profiling will be required to quantitate the overall impact on the specific pathways discussed below.

Regarding metabolism specifically, citrate synthase (Cs), a key enzyme catalyzing the first step in the Krebs cycle, demonstrated a 1.21-fold increase. Critical enzymes in the fatty acid oxidation pathway Acot2 and Acaa2 exhibited some of the highest fold changes (1.47 and 1.41, respectively), consistent with the β-oxidation activity in GO Enrichment Analysis (Figure 4). EtOH consumption also increased the abundance of related subunits Hadha and Hadhb (1.27- and 1.26-fold changes, respectively), which are subunits for the octamer mitochondrial trifunctional protein (MTP). MTP localizes to the inner mitochondrial membrane and serves as an important biomarker for mitochondrial health [17], previously shown to be essential for fatty acid β-oxidation and cardiolipin remodeling [18,19] and to be a link between fatty acid oxidation and OXPHOS [20]. These DEPs and the GO Enrichment Analysis emphasize the impact of ethanol on mitochondrial function and the disruption of cellular bioenergetics through lipid oxidation pathways in ACM. The most abundant molecular changes at this stage of ACM pathogenesis involve impaired metabolic remodeling at the mitochondrial matrix (Figure 4; Table 3), in agreement with previous studies [6,10]. Our results are consistent with previous studies that found an increase in fatty acid influx into cardiomyocytes due to ethanol exposure [10,21,22,23].

The healthy adult heart produces and consumes vast quantities of ATP necessary to sustain contractile function. The mitochondrial oxidative phosphorylation is the primary source of energy, contributing to ~95% of ATP production, while glycolysis supplies the rest of the ATP [24]. Under normal conditions, fatty acids are the primary substrate for ATP production in the heart, with ~60% of ATP being derived from mitochondrial fatty acid oxidation. Another remarkable ability of the heart is its metabolic flexibility to switch between different substrates (fatty acids, ketone bodies, amino acids, and pyruvate) [24]. The uptake and utilization of these substrates are tightly regulated, as the heart has a limited ability to store them intracellularly. Dysregulation of these processes, leading to changes in fatty acid influx and oxidation, has been reported in different pathological conditions that cause heart failure [25]. Myocardial fatty acid oxidation was reported to increase in heart failure associated with obesity and diabetes and to decrease during ischemia-induced cardiac dysfunction [24,25]. Enhanced fatty acid oxidation results in the inhibition of glucose oxidation due to a negative regulatory loop between these two processes, known as the Randle cycle or glucose fatty acid cycle [26]. Increased levels of metabolic intermediates (acetyl-CoA and NADH), generated by fatty acid oxidation, inhibit pyruvate dehydrogenase, a key enzyme required for glucose oxidation. An increase in another Krebs cycle intermediate, citrate, inhibits phosphofructokinase-1, the enzyme that catalyzes the rate-limiting step in glycolysis [27]. This type of metabolic regulation is considered an adaptive response in healthy cells since it ensures the most efficient use of fuel sources based on energy demand. It is, however, suggested to be maladaptive in disease states, as it leads to the loss of cardiac metabolic flexibility under energetically demanding conditions of a failing heart, impaired glucose metabolism, and cellular energy deficits [27]. Additionally, enhanced fatty acid influx into the mitochondria or reduced fatty acid oxidation may contribute to excess lipid deposition in the heart and the formation of lipid droplets, a phenomenon known to cause mitochondrial dysfunction [28]. Future studies will investigate the underlying molecular mechanisms of metabolic rewiring in ACM. Experiments that directly examine mitochondrial fatty acid transport, regulation of fatty acid oxidation, and other metabolic pathways will be required to fully characterize metabolic changes in ACM. Another critical aspect is the investigation of mitochondrial bioenergetic function following alcohol exposure with a variety of methods that allow the assessment of mitochondrial uncoupling and the efficiency of ATP production (e.g., simultaneous measurements of mitochondrial oxygen consumption rate and membrane potential with Oroboros high-resolution respirometry, ATP/ADP ratio, and calcium handling).

Notably, other DEPs of interest involve cytoskeletal architecture and oxidative pathways. Csrp3 (cysteine and glycine rich protein) localizes to the Z-disc and is involved in cardiac mechanosensory processes [29]. Mutations in this gene previously have been shown to cause heritable forms of HCM and DCM [30,31]. Thus, its downregulation (0.74-fold change) may serve as a precursor to the structural dysregulation previously observed in the ACM phenotype. In contrast, upregulation of vinculin (1.26-fold change) and tropomyosin 1 (1.83-fold change) implies changes in intercellular adhesion, cell-matrix interaction, mechanosensation, and cytoskeletal dynamics. Vinculin overexpression has been associated with focal adhesion complex formation at the site of integrin binding and may have implications in the transmission of contractile forces and in heart failure [32,33]. Regarding oxidative pathways, catalase demonstrated a 1.51-fold increase, suggesting altered peroxisomal activity secondary to ethanol-induced free radicals. Concurrently, downregulated DEPs pertaining to oxidation included monoamine oxidase A (Maoa, 0.51-fold change), haptoglobin (Hp, 0.50-fold change), and murinoglobulin 1 (Mug1, 0.78-fold change), further supportive of oxidative dysregulation. In cardiomyocytes specifically, altered monoamine oxidase expression has been linked to oxidative stress, mitochondrial dysfunction (e.g., calcium homeostasis), and cardiac damage [34,35]. Reduced levels of haptoglobin may be reflective of enhanced hemolysis and may predispose anemia [36,37]. These cytoskeletal elements and oxidative markers may provide future investigators target proteins of interest in ACM pathogenesis and other forms of heart failure. All other DEPs with a significant fold change are included in Table 3.

### 3.3. Mechanochemical Properties of Myosin

Muscle contraction is driven by the actomyosin ATPase cycle, in which the energy from ATP hydrolysis is used to produce actin filament sliding and muscle shortening, effectively linking chemical and mechanical processes [38,39]. Given the reactive nature of ethanol and its metabolites, it is plausible that oxidative changes to myofilamentous proteins may result in deficits of the mechanochemical properties of cardiac myosin. Previous work has shown reduced myofibrillar ATPase at high calcium and reduced actin-activated myosin ATPase in ethanol-fed hamsters [40]. Others have demonstrated a significant shift in myosin isoforms following a 12-week exposure in adult rats that also corresponded to a decrease in myosin ATPase. Gel electrophoresis demonstrated an increase in β-cardiac myosin from 9.7% in the control group to 35.1% in the EtOH group with a concomitant 30% decrease in myosin Ca^2+^ ATPase [41]. These authors attributed the decrease in ATPase to the inherent differences in the mechanochemical properties of α- vs. β-cardiac myosin [42]. Although the previous work demonstrated alcohol exposure can impact cardiac myosin function and/or isoform expression, no studies have fully characterized myosin motor function with in vitro motility assays, nor have they examined the autoinhibited super-relaxed state of myosin.

In the present data set, there were no detected changes between groups in myosin heavy chain isoforms, nor was there a change in motor function, as measured by unloaded in vitro motility (Figure 5; Table 4). Moreover, no change was detected in the low-salt ATPase in synthetic thick filaments (Figure 6A). However, low-salt myosin ATPase (Mg^2+^-ATPase) in the absence of actin is quite slow (~0.02–0.05 s^−1^); thus, small changes in activity may be difficult to detect. In contrast, the high-salt ATPase of the extracted myosin demonstrated a 15% decrease in the EtOH group (Figure 6B), as the high-salt conditions enhance the ATPase activity and provide a clear indication of the number of active myosin heads. This result coincides with the 22% increase in oxidation of the heavy chain of cardiac myosin in the ethanol group (Figure 6F), supporting the hypothesis that myosin oxidation may reduce the number of active myosin heads. Single turnover measurements in myosin dialyzed into low-salt conditions revealed no changes in SRX fraction, SRX rate, or DRX rates between the groups (Figure 6C–E), suggesting oxidative damage may not reduce the ATPase activity by increasing the fraction of myosin in the autoinhibited SRX state. Importantly, the extracted myosin was subjected to an actin spindown purification step prior to the in vitro motility, synthetic thick filaments formation, and myosin dialysis for single turnover. Thus, the possibility that damaged or oxidized myosin heads may have been removed at this step cannot be excluded, which would desensitize any assay following this purification step. In addition, a “dead-head” actin-blocking step was used in the in vitro motility assay to prevent damaged heads from slowing the actin gliding generated by active myosin heads. This possibility is further explored below.

### 3.4. Ethanol and Oxidation

Ethanol and its metabolites permeate membranes to initiate the propagation of oxidative reactions through reactive oxygen species (ROS), resulting in damage to proteins, lipids, and nucleic acids [43]. Previous studies have demonstrated that in vivo and in vitro oxidation can impact the structure of cardiac myosin, associated with potential denaturation and a reduction in mechanochemical function [44,45,46,47]. Accordingly, protein carbonylation of the myosin heavy chain was measured directly via OxyBlot assays. This analysis demonstrated a small but significant increase from 49.2% in the control group to 60% in the EtOH group, an overall 22% increase in carbonyl content (Figure 6F; Table 4). No changes were detected in myosin light chains, implying they are protected from oxidative insult relative to myosin heavy chain residues. Evidence of oxidative stress is further supported by the mRNA analysis, which demonstrated a trend of an increase in the expression of CAT and TNF⍺ and a significant reduction in IFN⍺ and IFNβ (Supplemental Figure S6), as oxidative stress influences the production of antioxidant enzymes and pro-inflammatory mediators [44].

As previously stated, it is plausible that a significant portion of the oxidized myosin was removed via the actin spindown, effectively desensitizing the OxyBlot assay. Accordingly, to measure the direct impact of protein oxidation on myosin mechanochemistry, functional assays were repeated following an in vitro oxidation of purified healthy myosin with a mild (1 mM H_2_O_2_) and high (100 mM H_2_O_2_) treatment of exogenous in vitro oxidation. These treatments demonstrated a clear, dose-dependent, stepwise decrease in in both ATPase and in vitro motility, demonstrating a direct link between oxidation and deficits in cardiac myosin mechanochemistry (Figure 7; Table 5). Oxidative effects of specific myosin residues and subsequent changes in myosin function have been shown previously in both cardiac [48] and skeletal muscle [49,50], and thus, this data set contributes to our understanding of cardiac myosin and the ACM disease phenotype specifically.

### 3.5. Limitations

This study does not come without limitations. Notably, the study lacks in vivo functional testing such as echocardiography to better understand organ-level cardiac function throughout the 16-week time course, which may impact the translational scope of the conclusions. While 16 weeks previously has been demonstrated to be sufficient to induce cardiac changes in rats such as reduced stroke volume, cardiac output, and end diastolic volume [51], 24 weeks of ethanol exposure demonstrated much more pronounced effects [21]. Thus, having multiple time points of ethanol exposure (e.g., 8 weeks, 16 weeks, and 24 weeks) would strengthen the interpretation of disease pathogenesis. Additionally, these studies focused solely on the Lieber–DeCarli diet model for EtOH exposure. While all animals were euthanized at the same time point, group variability in the BAC data is likely induced by individual differences in consumption as well as timing of the last drinking session prior to euthanasia, which cannot be fully standardized with the Lieber–DeCarli method. However, even with the inherent variability from the model chosen (Supplementary Figure S1B), significant impacts were found in cardiac tissues between groups. It is important to note that the one-time BAC sampling in these studies was used as a snapshot of consumption, but the single BAC measure should not affect the overall interpretation of the study, which is dependent on the long-term EtOH consumption rather than acute EtOH effects. Future studies should further examine the impact of other diets on the outcomes here as well as differences between forced versus self-administered EtOH intake models. Regarding myosin extraction, future mechanochemical and oxidation experiments may perform the assay prior to the actin spindown, which would allow insight into the totality of myosin activity in extracted motors in hearts from the control and ethanol-consuming rats. Notably, the OxyBlot assay employed is specific to protein carbonylation, which is a proxy for overall oxidation and excludes important measures such as nitrosylation and lipid peroxidation. Lastly, the current study included only male rats, which was designed due to the discrepancy of ACM incidence in men vs. women (9:1), though post-menopausal women have been reported to be at pronounced risk for the disease [52].

### 3.6. Summary

Alcoholic cardiomyopathy is a multi-factorial disease, previously demonstrated to be associated with changes in protein balance, calcium handling, protein oxidation, and mitochondrial dysfunction. Our data demonstrate that metabolic remodeling, specifically an upregulation of β-oxidation at the inner mitochondrial membrane, precedes major structural remodeling and that small, significant changes in myosin mechanochemistry are components of early ACM pathogenesis. The DEPs and metabolic processes identified within the current study may serve as disease markers and may form the basis for future investigation into a more complete understanding of ACM pathogenesis. Future studies on ACM should emphasize specific mitochondrial substrate analyses to better understand how changes in mitochondrial morphology and machinery induce metabolic rewiring and impact cellular bioenergetics.

## 4. Materials and Methods

### 4.1. Animal Care and Handling

A total of 22 pathogen-free Sprague-Dawley (Crl:SD) adult male rats 2 months of age (Charles River, Cambridge, MA, USA) were utilized in this study (Figure 1). Upon arrival at the Pennsylvania State University College of Medicine, the rats were housed 2 per cage during a one-week quarantine period and were provided standard rodent chow (Envigo Global no. 8604 diet; percent calories from protein 32%, from fat 14%, and from carbohydrates 54%; Envigo Teklad; Boston, MA, USA) and water ad libitum. All rats were kept in a temperature- (22–24 °C) and humidity- (50–60%) controlled environment with a 12 h light/dark cycle. At the conclusion of the acclimation period, rats were housed in solid-bottom cages with corncob bedding to avoid foot irritation. Block randomization was used to assign rats to either an ethanol-containing liquid diet (*n* = 12) or isocaloric isonitrogenous (no ethanol) control diet (*n* = 10) to minimize bias [53].

Each group was maintained for 16 weeks on the nutritionally adequate Lieber–DeCarli liquid diet (Bio-Serv, Frenchtown, NJ, USA). While shorter durations of ethanol intake yield reproducible results in other organs (e.g., liver), it previously has been determined that longer durations of ethanol consumption are necessary to produce evidence of alcoholic cardiomyopathy [10]. Rats consuming the ethanol-containing diet initially received 12% of total calories from ethanol, and this percentage was increased weekly by 12% until a maximum of 36% of caloric intake from ethanol was achieved. Time-matched pair-fed control animals received a liquid diet where maltose-dextran was isocalorically substituted for ethanol. Consumption of the liquid diet was assessed daily, and animals were weighed weekly. Ethanol consumption in this model typically produces blood ethanol levels that are comparable to that observed in human subjects.

Animal handling and all experiments discussed within were approved by the Institutional Animal Care and Use Committee of the Pennsylvania State University College of Medicine (IACUC ID: PROTO201800341) and adhere to the NIH guidelines for the use of experimental animals.

### 4.2. Sacrifice and Harvesting of Tissue

Prior to euthanasia, rats were anesthetized deeply with isoflurane (3–4% + oxygen). Animals were all euthanized at the same time of day, blood was collected from the abdominal aorta for blood alcohol content (BAC) determination, and then, all cardiac tissue was excised immediately. This method is consistent with the recommendations of the AVMA Guidelines for the Euthanasia of Animals. The cardiac tissue was rinsed briefly in 1× PBS, dabbed dry, and then weighed. The cardiac tissue then was dissected and sectioned in a reproducible manner. A cut was made at the atrioventricular junction, and the atria were discarded. A coronal section of the superior-most portion of the ventricles was taken and immediately placed in 10% formalin to begin the fixation process for histology (FFPE: formalin–fixed paraffin embedded). The remaining portion of the ventricle was frozen in liquid nitrogen and immediately powderized using a mortar and pestle. The powderized tissue was separated into aliquots (roughly 150 mg) for myosin extraction.

### 4.3. Blood Alcohol Content

The BAC content was determined using the well-validated Analox GL-5 Alcohol Analyzer system (Analox Instruments, Stourbridge, UK), according to manufacturer instructions. The Analox system takes advantage of the ability of ethanol oxidase to oxidize ethanol and create acetaldehyde and hydrogen peroxide. Under these conditions, oxygen consumption is directly proportional to ethanol concentration. Sample retest variability in this system is 2.5%; thus, two samples per animal were analyzed in duplicate, and the average per animal was reported. 

### 4.4. Routine Histology, Electron Microscopy, and Analysis

Cardiac tissue for histology underwent routine formalin fixation and paraffin embedding in the Pennsylvania State University College of Medicine Molecular and Histopathology Core. Sections at 5 microns were collected and stained with hematoxylin and eosin (H&E) or Masson’s trichrome. Prepared slides were visualized using a Leica Aperio GT450 Digital Pathology Scanner (Wetzlar, Germany) from 4 to 40×. Cell diameter was measured in histological cross-sections of the cardiac tissue using ImageJ, v1.54p (NIH). To account for variations in myocardial syncytium, only clear sections of cardiomyocyte cross-sections were included in the analysis. We located sections of the syncytium that were in cross-section that were clearly demonstrating delineation between adjacent fibers. We also located sections of the syncytium in which the cut was parallel with the fibers. Twenty measurements of cellular diameter were taken per animal, and these measurements were averaged to compare cellular dimensions between groups.

The FFPE histology blocks also were utilized to conduct ultrastructure analysis via electron microscopy. A punch biopsy of the block of the left ventricle was taken from six animal blocks per group. The sample was deparaffinized and fixated in glutaraldehyde and osmium tetroxide prior to dehydration and acetone/epoxy resin infiltration. Samples were embedded in BEEM capsules and polymerized in an oven overnight (60 °C). Following screening of 1-micron sections, 70–85 nm sections were collected with a Leica EM UC7 instrument. Samples were stained with 2% uranyl acetate, lead citrate, rinsed, and then visualized on the JEOL JEM 1010 transmission electron microscope (Tokyo, Japan). Images were magnified at 5000, 10,000, 25,000, and 50,000× and collected via an AMT Hamamatsu Orca-HR digital camera and imaging system (Hamamatsu, Japan). Ultrastructure EM images at 10,000× were used for analysis of sarcomeric length (Z-line to Z-line) and for mitochondrial density and lucency via ImageJ. Mitochondria were manually segmented throughout the images to ensure precise identification. Mitochondrial area was measured via densitometric methods, while the level of swelling (electron lucency) was determined by measuring the mean pixel intensity within the selected regions [54,55].

### 4.5. Mass Spectrometry

To determine the proteomic profile of the ethanol and control groups, liquid chromatography–mass spectrometry (LCMS) was performed. A 1–5 mg piece of each sample was sent to the University of Vermont on dry ice. In brief, each piece of muscle was placed in a glass-bottom dissection chamber containing 150 μL 0.1% Rapigest SF Surfactant (Waters Corporation; Milford, MA, USA) and mechanically triturated with forceps. The solubilized proteins were reduced by addition of 0.75 μL 1 M dithiothreitol (DTT) and heating at 100 °C for 10 min. Proteins were alkylated by addition of 22.5 μL of 100 mM iodoacetamide (Acros Organics; Geel, Belgium) in 50 mM ammonium bicarbonate, followed by a 30 min incubation in the dark at 22 °C. The proteins were cleaved into tryptic peptides by addition of 25 μL of 0.2 μg/μL trypsin (Promega; Madison, WI, USA) in 50 mM ammonium bicarbonate and incubation for 18 h at 37 °C. Following the digestion, the samples were dried by centrifugal evaporation and reconstituted in 100 μL of a 7% formic acid in 50 mM ammonium bicarbonate solution to inactivate trypsin and degrade Rapigest (1 h, 37 °C). Samples were dried once more and reconstituted in 100 μL 0.1% trifluoroacetic acid (TFA) for further cleavage of Rapigest (1 h, 37 °C). Samples were dried a final time, reconstituted in 100 μL 0.1 TFA, and centrifuged for 5 min at 18,800 g (Thermo, Sorvall LegendMicro 21 R; Waltham, MA, USA), and then, 75 μL of the supernatant was removed for analysis by LCMS15. Briefly, a 20 μL aliquot of each sample was injected onto an Acquity UPLC HSS T3 column (100 Å, 1.8 μm, 2.1 mm × 150 mm, Waters Corporation; Milford, MA, USA) attached to an UltiMate3000 ultrahigh-pressure liquid chromatography (UHPLC) system (Dionex; Sunnyvale, CA, USA). The UHPLC effluent was directly infused into a Q Exactive Hybrid Quadrupole-Orbitrap mass spectrometer (Thermo Fisher Scientific; Waltham, MA, USA). Data were collected in data-dependent MS/MS mode. Peptides were identified by SEQUEST, and LC peak areas of peptides of interest, being those unique to or shared between protein isoforms, were extracted using the Proteome Discoverer 2.2 software package. LC peak areas were imported into Excel for further analysis [56]. The average abundance of the top 3 peptides unique to any protein or those shared between multiple protein sequences was normalized to the sum of the abundance for all proteins identified in each sample. The average relative abundance (±SD) was reported.

Mass spectrometry results were filtered by differentially expressed proteins (DEPs) that demonstrated >20% change (upregulation or downregulation) with a *p*-value < 0.01. The DEPs that met these thresholds then were input into Gene Ontology (GO) Enrichment Analysis via PANTHER Overrepresentation Test [57,58]. The three categories (biological processes, molecular function, and cellular components) were included in the analysis. The negative natural logarithm of the *p*-values generated from the software was calculated to produce the GO Enrichment Scores, an indication of the degree of differential expression.

### 4.6. Myosin Extraction

Full-length myosin was extracted directly from rat cardiac tissue, adapted from previously described protocols [59,60]. Briefly, powderized tissue was retrieved from liquid nitrogen, and 150 mg was weighed for extraction. The tissue was placed in extraction buffer consisting of 400 mM KCl, 150 mM K_2_HPO_4_, 10 mM Na_4_P_2_O_7_, 1 mM MgCl_2_ (pH 6.8), 2 mM DTT, 1 mM PMSF, and 10 μg/mL aprotinin and leupeptin and homogenized for 10 min. The samples were spun for 5 min at 10,000 g at 4 °C and then spun in an ultracentrifuge for 20 min at 53,000 rpm at 4 °C (TLA120.2 rotor) to remove cellular debris. The myosin-containing supernatant was then precipitated by 10-fold dilution with dH_2_O with 2 mM DTT for 60 min on ice. The samples were spun once more for 10 min at 10,000 g at 4 °C, and the pellets were rinsed with cold dH_2_O and resuspended in high-salt resuspension buffer, containing 25 mM imidazole, 600 mM KCl, 1 mM EGTA, and 4 mM MgCl_2_ (pH 7.4). To remove “dead heads” from the samples, myosin was further purified using an actin spindown protocol in which F-actin was added at 1.5× molar ratio relative to myosin concentration. The actomyosin was pelleted by ultracentrifugation (95,000 rpm in TLA120.2 rotor, 15 min, 4 °C) and then released in MOPS300 buffer containing 10 mM MOPS, pH 7.0, 300 mM KCl, 1 mM EGTA, 1 mM MgCl_2_, and 1 mM DTT, in the presence of 2 mM ATP (95,000 rpm in TLA120.2 rotor, 10 min, 4 °C). Final myosin concentrations were calculated via Bradford assay, and the quality of the extraction was assessed by SDS-PAGE.

### 4.7. In Vitro Motility

The in vitro motility (IVM) assay was performed with the full-length cardiac myosin purified from rat hearts as described above, utilizing previously established protocols [59,61]. Briefly, microscope cover slips were coated with 1% nitrocellulose in amyl acetate (Ladd Research) and applied to a microscope slide with double-sided tape to create a flow cell. Myosin in MOPS300 buffer at a concentration of 0.125 mg/mL (0.5 μM) was applied directly to the nitrocellulose surface, and the surface subsequently was blocked with BSA (1 mg/mL). Unlabeled sheared actin (2 µM) followed by ATP (2 mM) was added to ensure blocking of inactive myosin heads (“dead heads”). Fluorescently labeled actin (10 nM final, labeled with phalloidin-Alexa 555; Thermo Fisher Scientific; Waltham, MA, USA) was then added to the flow cell. To initiate motility, an activation buffer containing 0.35% methylcellulose, an ATP regeneration system (2 mM ATP, 5 mg/mL glucose, 46 U/mL pyruvate kinase, and 0.46 mM phosphoenolpyruvate), oxygen scavengers (0.1 mg/mL glucose oxidase, 0.018 mg/mL catalase), and 10 mM DTT was added. The slide was visualized promptly with a NIKON TE2000 microscope (DsRedfilter; excitation/emission 555/588 nm; Tokyo, Japan) equipped with a 60×/1.4 NA phase objective and a Perfect Focus System. All images were acquired at 1 s intervals for 2 min using a shutter-controlled CoolSNAP HQ2 cooled CCD digital camera (Photometrics) binned 2 × 2. Temperature (25 ± 1 °C) was monitored using a thermocouple meter (Stable Systems International). Videos were exported to ImageJ and prepared for automated FAST software (Fast Automated Spud Tracker, v1.0.1; Stanford, CA, USA) motility analysis [62], from which > 1000 actin filaments from one experiment (i.e., slide) per myosin extraction (*n* = 8) at 0.5 µM myosin were compiled for statistical analysis. The sample means of independent experiments were averaged, and the data were presented as a SuperPlot, as previously described [63,64].

### 4.8. Synthetic Thick Filament Formation

Synthetic thick filaments (STFs) were reconstituted from purified myosin, adapted from previously established protocols [12]. Briefly, myosin in high-salt buffer (MOPS300) (10 mM MOPS, pH 7.0, 300 mM KCl, 1 mM EGTA, 1 mM MgCl_2_, and 1 mM DTT) was diluted 10-fold with MOPS0 (identical to MOPS300 but with 0 mM KCl) to reduce the ionic strength (final concentration of 30 mM KCl). At lower ionic strength, myosin molecules in solution can reconstitute into thick filaments, previously coined synthetic thick filaments (STFs). The diluted sample was placed on a rocker at 4 °C overnight, and a precipitate was visible in the solution the following day. Samples were gently homogenized, and 50 μL was used to assess filament formation by ultracentrifugation. The samples were spun in an ultracentrifuge for 10 min at 95,000 rpm and 4 °C (TLA120.2 rotor). The supernatant was removed, and the pellet was resuspended in 50 μL of 6 M urea. Supernatant and pellet samples were run in tandem on an SDS gel to assess the degree of filament formation.

### 4.9. Synthetic Thick Filament ATPase

A low-salt ATPase assay was conducted using an Applied Photophysics stopped-flow apparatus (Surrey, UK) to measure the steady-state ATPase activity of synthetic thick filaments. The assay utilized an NADH-coupled system in an MOPS30DTT buffer, which included 10 mM MOPS (pH 7.0), 30 mM KCl, 1 mM EGTA, 1 mM MgCl_2_, and 1 mM DTT at 25 °C. Upon the addition of 1 mM ATP, changes in absorption at 340 nm were recorded for 200 s, and the resulting transients were fitted to a linear function. A standard curve generated from known ADP concentrations was employed to determine the ATPase rate. Myosin concentration was determined using a Bradford assay prior to dilution.

A high-salt ATPase assay was performed at room temperature (22 ± 1 °C) in an NH_4_^+^ ATPase buffer containing 25 mM Tris (pH 8.0), 0.4 M NH_4_Cl, 2 mM EDTA, and 0.2 M sucrose, as previously described [65]. The reaction was initiated by adding 4 mM NH_4_^+^-ATP to a solution containing 0.2 µM myosin. Samples were taken at set time intervals, every 1 min over a 5-min period, and the reaction was quenched with a stop solution containing 60 mM EDTA (pH 6.5) and 6.6% SDS. The amount of phosphate liberated over time was determined using a colorimetric method involving color development with 0.5% ammonium molybdate and 0.5% ferrous sulfate in 1 N H_2_SO_4_. Absorbance was read using a plate reader at 620 nm after a 15-min incubation at room temperature. A standard curve with known phosphate concentrations was used to determine the free phosphate concentration. The experiment was performed in triplicate for each preparation.

### 4.10. Single Turnover Assay

In the single turnover measurements, the fluorescence of 2′-deoxy-ATP labeled with N-Methylanthraniloyl at the 3′-ribose position (mant-ATP) (Jena Bioscience, NU203; Jena, Germany) was monitored using 290 nm excitation and a 395 nm long pass emission filter. Myosin was first precipitated and dialyzed overnight in MOPS30DTT buffer, and experiments were conducted in the same buffer. For the measurements, 1 μM myosin was incubated with 20 μM mant-ATP for 30 s at room temperature before rapidly mixing the complex with a saturating concentration of ATP (4 mM) to achieve final concentrations of 0.5 µM myosin, 10 µM mant-ATP, and 2 mM ATP. Upon binding to myosin, the mant-ATP fluorescence increases, mant fluorescence is unchanged during the ATP hydrolysis and phosphate release steps, and mant-ADP fluorescence decreases upon dissociation from myosin. The slowest step in the cycle is the release of phosphate from myosin, and thus, the entire cycle time can be monitored with the single turnover method. The fluorescence decay was monitored over 1000 s to observe the single mant-ATP turnover from myosin chased by dark ATP. Myosin can populate an autoinhibited state with 5–10-fold slower ATPase, known as the super-relaxed state (SRX), and an uninhibited state known as the disordered-relaxed state (DRX). Thus, the mant fluorescence transients typically follow a two-exponential decay with the slow phase representing the SRX rate constant and the fast phase representing the DRX rate constant. The data were analyzed using a two-exponential fitting model to determine the SRX/DRX ratio, SRX rate, and DRX rate [66,67].

### 4.11. Actin Preparation

F-actin used in myosin extraction assay was purified from rabbit skeletal acetone powder, as previously described [68].

### 4.12. Protein Carbonylation of Cardiac Myosin

To assess differences in protein oxidation, a Protein Carbonyl Assay Western Blot Kit (Abcam, ab178020; Cambridge, UK) was utilized. Residual myosin samples from extractions were flash-frozen in liquid nitrogen and stored at −80 °C. Samples were thawed in parallel and diluted down to 2.0 μM in preparation for a derivation reaction with 2,4 dinitrophenylhydrazine (DNPH), which reacts specifically with protein carbonyl groups, a common marker of protein oxidation. Each protein sample was subjected to a DNPH reaction and a derivation control reaction for 15 min at room temperature. The samples then were neutralized and run on a 15% polyacrylamide gel. The gels were transferred to a PVDF membrane, which was incubated with DNPH primary antibody overnight, washed, and then incubated with horseradish peroxidase (HRP) goat anti-rabbit secondary antibody before development with enhanced chemiluminescence (ECL). Both antibodies were supplied with the Abcam kit. SDS-PAGE was performed with approximately 100 ng of protein per well to visualize the signal for the myosin heavy chain (MHC). Coomassie gels were run in tandem to confirm protein concentration of the protein samples. Densitometry of myosin heavy chains in both Western blots as well as Coomassie SDS gels were calculated using ImageJ. Oxidation densitometry was normalized to a carbonylated BSA standard on each blot, and protein concentration was calculated using a myosin standard. The degree of protein oxidation was expressed as the ratio (Western blot densitometry/Coomassie densitometry) for each protein sample. Ratios were averaged by group (control vs. ethanol) and then compared statistically by a Student’s *t*-test.

### 4.13. In Vitro Oxidative Treatment

To further examine the impact of oxidation on myosin, in vitro oxidation experiments on cardiac muscle myosin extracted from healthy rats were performed. Cardiac myosin was exposed to increasing amounts of exogenous H_2_O_2_, with a mild treatment at 1 mM H_2_O_2_ and a high oxidative treatment using a hydroxyl radical-generating system (10 μM FeCl_3_, 1 mM ascorbic acid, 100 mM H_2_O_2_) for 30 min at room temperature. The oxidation reaction was terminated by adding 2% β-mercaptoethanol, followed by overnight dialysis to remove residual reactants.

### 4.14. mRNA Expression

Total RNA from rat heart tissue was extracted with TRIzol (Invitrogen, 15596026; Waltham, MA, USA) and reverse transcribed using the High Capacity cDNA kit (Applied Biosystems, 4374966; Foster City, CA, USA). Quantitative real-time PCR was performed using Quantitect SYBR Green Master Mix (Qiagen, 204143; Hilden, Germany) on a QuantStudio 12K Flex Real-Time PCR System (Thermo Fisher Scientific; RRID:SCR_021098; Waltham, MA, USA). Expression of target mRNAs was normalized to GAPDH using the 2^−ΔΔCT^ method. PCR primer sequences are listed in Supplemental Table S1.

### 4.15. Statistics

All measures were compared using parametric Student’s *t*-tests (Control vs. EtOH) with statistical significance set at *p* ≤ 0.05 unless indicated otherwise.

## Figures and Tables

**Table 1:** Animal characteristics at sacrifice (16-week treatment).

	Body Weight (g)	Heart Weight (mg)	HW–BW (mg/g)	Serum (BAC) (mg/dL)
Control (*n* = 10)	325 ± 19.5	883 ± 54	2.72 ± 0.09	1.93 ± 0.99
EtOH (*n* = 12)	300 ± 23.1	834 ± 76	2.77 ± 0.11	35.3 ± 25.5
*p*-value	0.0163	0.1031	0.2635	0.0005

**Table 2:** Histology and EM measurements.

	Cell Size (Cross-Section) (µm)	Sarcomere Length * (nm)	Mitochondrial Area * (µm^2^)	Mitochondrial Lucency *
Control (*n* = 10)	15.42 ± 2.44	1918 ± 222	0.45 ± 0.05	84.18 ± 7.68
EtOH (*n* = 12)	15.67 ± 1.53	1649 ± 377	0.93 ± 0.20	110.40 ± 19.32
*p*-value	0.7761	0.164	0.0002	0.0115

* Sarcomere length and mitochondrial analysis (*n* = 6).

**Table 3:** Summary of mass spectrometry.

Gene	Accession	Description	Fold Change	*p*-Value
*Myl4*	P17209	Myosin essential (alkali) light chain, atrial/fetal	0.0711	5.98 × 10^−4^
*Nppa*	P01161	Naturietic peptide A	0.3614	2.87 × 10^−4^
*Hp*	P06866	Haptoglobin	0.5042	2.43 × 10^−5^
*Maoa*	G3V9Z3	Monoamine oxidase A	0.5191	1.51 × 10^−6^
*Mug1*; *A1i3*	Q03626; P14046	Murinoglobulin 1 + alpha 1-inhibitor 3	0.6320	3.56 × 10^−4^
*Eno3*	P15429	Enolase 3	0.6765	4.64 × 10^−3^
*Ckb*	P07335	Creatine kinase B	0.6767	3.18 × 10^−4^
*Csrp3*	G3V7U0	Cysteine- and glycine-rich protein	0.7421	6.70 × 10^−3^
*Mug1*	Q03626	Murinoglobulin 1	0.7824	8.73 × 10^−3^
*Cs*	G3V936	Citrate synthase	1.2097	6.45 × 10^−3^
*Suclg2*	B1H270	Succinate-CoA ligase subunit B	1.2122	1.66 × 10^−6^
*Pygb*	B2GV03	Glycogen phosphorylase	1.2468	3.91 × 10^−3^
*Hadhb*	Q60587	Hydroxylacyl-coenzyme A dehydrogenase trifunctional complex subunit B	1.2563	1.86 × 10^−7^
*Vcl*	P85972	Vinculin	1.2609	1.71 × 10^−3^
*Hadha*	Q64428	Hydroxylacyl-coenzyme A dehydrogenase trifunctional complex subunit A	1.2679	9.55 × 10^−7^
*Hadh*	Q9WVK7	Hydroxylacyl-coenzyme A dehydrogenase	1.2762	6.20 × 10^−4^
*ATP8*	Q5UAJ5	ATP synthase protein 8, mitochondrial	1.3721	2.81 × 10^−3^
*Decr1*	G3V734	2,4-dienoyl-CoA reductase	1.3817	6.78 × 10^−3^
*Scp2*	P11915	Non-specific lipid-transfer protein	1.3861	2.49 × 10^−6^
*Acaa2*	P13437	Acetyl-CoA acyltransferase 2	1.4164	1.23 × 10^−5^
*Acot2*	O55171	Acylcoenzyme A thioesterase 2	1.4683	6.47 × 10^−10^
*Cat*	P04762	Catalase	1.5054	2.18 × 10^−6^
*Tpm1*	F7FK40	Tropomyosin alpha-1 chain	1.8349	3.22 × 10^−3^

**Table 4:** Extracted myosin mechanochemistry.

Parameter	Control (*n* = 5)	EtOH (*n* = 5)	*p*-Value
Low-salt ATPase (s^−1^)	0.033 ± 0.009	0.027 ± 0.005	0.1906
High-salt ATPase (s^−1^)	16.9 ± 0.6	14.3 ± 1.1	0.0011
SRX fraction	0.22 ± 0.04	0.22 ± 0.06	1.000
SRX rate (s^−1^)	0.0049 ± 0.0016	0.0052 ± 0.0012	0.7549
DRX rate (s^−1^)	0.026 ± 0.004	0.023 ± 0.002	0.1720
IVM velocity (nm/s)	1732 ± 235	1732 ± 203	1.000
IVM stuck%	13.3 ± 6.7%	13.4 ± 4.3%	0.9902
Protein carbonylation	49.2 ± 5.0%	60.0 ± 6.3%	0.017

**Table 5:** Summary of in vitro oxidative treatments.

	High-Salt ATPase (s^−1^)	IVM Velocity (nm/s)
Untreated (*n* = 3)	17.83 ± 0.69	2414 ± 185
Mild treatment (*n* = 3)	12.24 ± 0.12	1106 ± 31
High treatment (*n* = 3)	1.71 ± 0.03	60 ± 16

## Data Availability

All of the data that support the findings in this study are included in the manuscript or the Supplemental Data.

## Supplementary Materials

The following supporting information can be downloaded at: https://www.mdpi.com/article/10.3390/ijms26146766/s1.

## Abbreviations

The following abbreviations are used in this manuscript:

ACM	Alcoholic cardiomyopathy
DCM	Dilated cardiomyopathy
SRX	Super-relaxed state
DRX	Disordered-relaxed state
IVM	In vitro motility
EM	Electron microscopy
H&E	Hematoxylin and eosin
BAC	Blood alcohol content
DEPs	Differentially expressed proteins
GO	Gene Ontology
CAT	Catalase
TNF⍺	Tumor necrosis factor alpha
IFN⍺/IFNβ	Interferon alpha/beta
SOD1/SOD2	Superoxide dismutase 1/2
GPX1	Glutathione peroxidase 1
NQ01	NAD(P)H quinone dehydrogenase 1
HO-1	Heme oxygenase 1
REDD1	Regulated in Development and DNA Damage Responses 1
CCL2/CCL5	Chemokine (C-C motif) Ligand 2/5
ICAM-1	Intercellular adhesion molecule 1
STF	Synthetic thick filament
MTP	Mitochondrial trifunctional protein
OXPHOS	Oxidative phosphorylation
PCR	Polymerase chain reaction
TFA	Trifluoroacetic acid
SDS-PAGE	Sodium Dodecyl Sulfate–Polyacrylamide Gel Electrophoresis
DNPH	2,4-Dinitrophenylhydrazine
MHC	Myosin heavy chain
BSA	Bovine serum albumin
ECL	Enhanced chemiluminescence
PBS	Phosphate buffered saline
DTT	Dithiothreitol
EGTA	Ethylene glycol tetraacetic acid
MOPS	3-(N-morpholino)propanesulfonic acid
UHPLC	Ultra-high performance liquid chromatography
LCMS	Liquid chromatography–mass spectrometry
NIH	National Institutes of Health
IACUC	Institutional Animal Care and Use Committee
PVDF	Polyvinylidene difluoride
HRP	Horseradish peroxidase
