# Supplementary material for: Early-Stage Alcoholic Cardiomyopathy Highlighted by Metabolic Remodeling, Oxidative Stress, and Cardiac Myosin Dysfunction in Male Rats
Source: Int J Mol Sci. 2025 Jul 15;26(14):6766. doi: 10.3390/ijms26146766 (PMC12296025; doi:10.3390/ijms26146766)
Supplement: Supplementary file 1 [file ijms-26-06766-s001.zip › ijms-3659198-supplementary.pdf]

# Supporting Information

# Early-Stage Alcoholic Cardiomyopathy Highlighted by Metabolic Remodeling, Oxidative Stress, and Cardiac Myosin Dysfunction in Male Rats

David V. Rasicci[1,2*], Jinghua Ge[1*], Adrien P. Chen[1], Neil B. Wood[3], Skylar ML Bodt[1], Allyson L. Toro[1], Alexandra Evans[4], Omid Golestanian[2], Md Shahrier Amin[2], Anne Pruznak[1], Nelli Mnatsakanyan[1], Yuval Silberman[4], Michael D. Dennis[1], Michael J. Previs[3], Charles H. Lang[1], Christopher M. Yengo[1**]

Affiliations:
[1]Department of Cell and Biological Systems, Penn State College of Medicine, Hershey, PA 17033
[2]Department of Pathology, Anatomy, and Laboratory Medicine, West Virginia University School of Medicine, Morgantown, WV 26505
[3]Department of Molecular Physiology and Biophysics, University of Vermont, Burlington, VT 05405
[4]Department of Neuroscience and Experimental Therapeutics, Penn State College of Medicine, Hershey, PA 17033

* co-first authors
**corresponding author, Email: cmy11@psu.edu. Phone: 717-531-8575

This PDF file includes:
Supplemental Table S1. Oligonucleotides Used in qPCR Analysis.
Supplementary Figure S1. Blood Alcohol Concentration and Alcohol Consumption.
Supplementary Figure S2. Volcano plot of differentially abundant proteins in the EtOH group compared to control.
Supplementary Figure S3. Myosin Extraction and Synthetic.
Supplementary Figure S4. Single ATP Turnover.
Supplementary Figure S5. Carbonylation Analysis.
Supplementary Figure S6. mRNA Expression for IFNα, IFNβ, CAT, and TNF.
Supplementary Figure S7. mRNA Expression for REDD1, CCL2, CCL5, ICAM, IL6, NQO1, HO-1, GPX1, SOD1, and SOD2.
Supplementary Table S2. Summary of Mass Spectrometry Results

Supplemental Table S1. **Oligonucleotides Used in qPCR Analysis**

| Gene | Forward Primer Sequence | Reverse Primer Sequence |
|---|---|---|
| CAT | CCGCCTTTTTGCTTACCCAG | GAGCACGGTAGGGACAGTTC |
| CCL2 | CACTCACCTGCTGCTACTCA | GCTTGGTGACAAAAACTACAGC |
| CCL5 | TGCTGCTTTGCCTACCTCTC | TCCTTCGAGTGACAAACACGA |
| DDIT4 | GGAGGACGAGAAACGATCCC | TCTTGTCCGCAATCTTCGCT |
| GAPDH | AGTTCAACGGCACAGTCAAG | TACTCAGCACCAGCATCACC |
| GPX1 | CAGTCCACCGTGTATGCCTT | GAGGGACGCGACATTCTCAA |
| HMOX1 | CAGGGAAGGCTTTAAGCTGGT | GGGTTCTGCTTGTTTCGCTC |
| ICAM1 | CTCACAGGGTACTTCCCCCA | CAGGTGAGGACCATATAGCAGA |
| IFNA1 | AGCAACAAAGCCTCAGGAACA | GGATCTGCTGGGCATCCATCT |
| IFNB1 | CTTGGGTGACATCCACGACT | AAGACTTCTGCTCGGACCAC |
| IL1B | AGGCTTCCTTGTGCAAGTGTC | TCATCTGGACAGCCCAAGTC |
| IL6 | CATTCTGTCTCGAGCCCACC | GCTGGAAGTCTCTTGCGGAG |
| NQO1 | ATTGTATTGGCCCACGCAGA | GATTCGACCACCTCCCATCC |
| SOD1 | TCATTGGCCGTACTATGGTGG | CCAATCACACCACAAGCCAAG |
| SOD2 | GGAGCAAGGTCGCTTACAGA | TCCCACACATCAATCCCCAG |
| TNF | ATGGGCTCCCTCTCATCAGT | GCTTGGTGGTTTGCTACGAC |

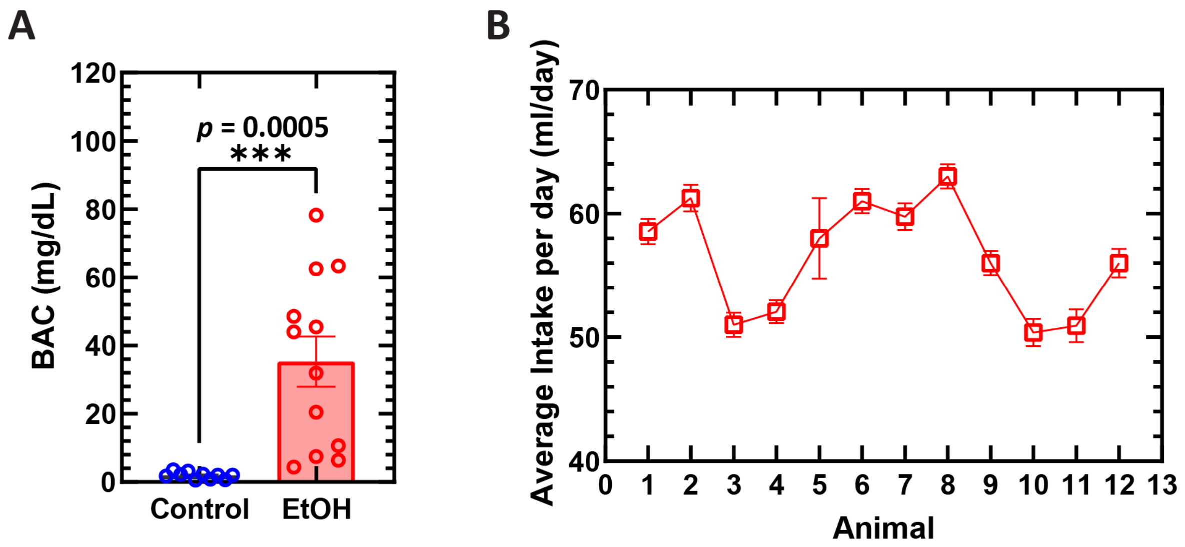


**Supplementary Figure S1.** Blood Alcohol Concentration and Alcohol Consumption. (A) Difference in blood alcohol levels between the control group (n = 10) and the EtOH group (n = 12) measured from blood serum at time of sacrifice. (B) Average daily alcohol consumption in the EtOH group. Data are presented as mean ± SEM, statistical significance was evaluated using an unpaired t-test.

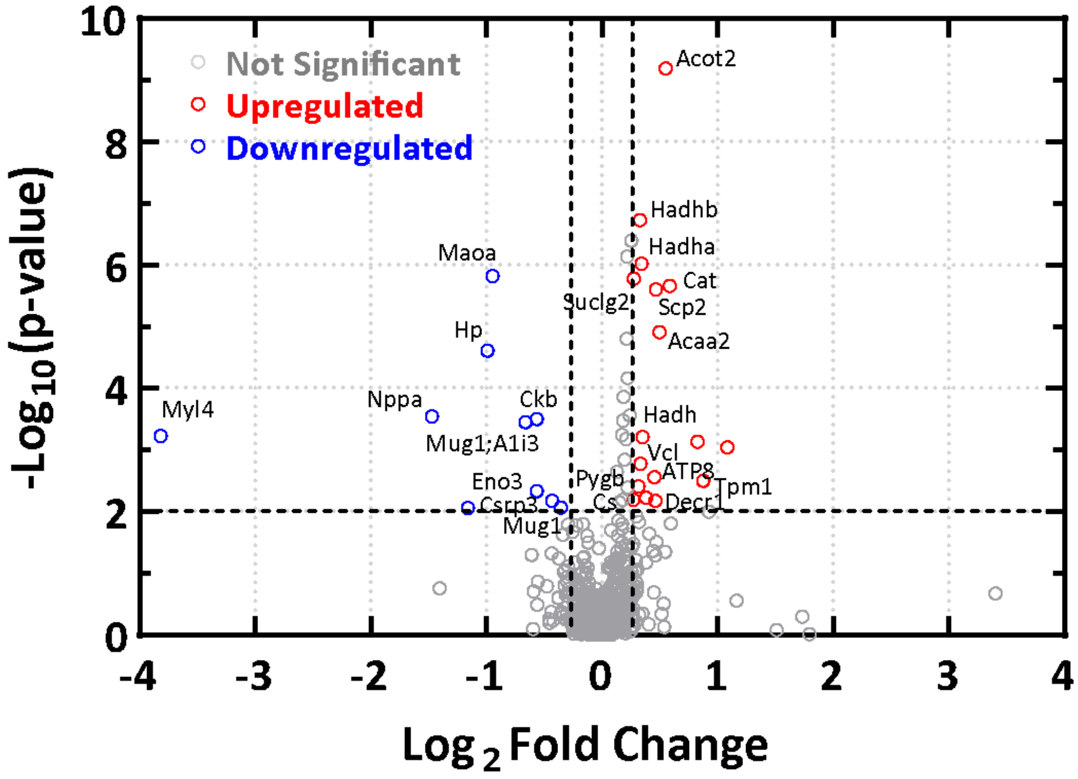


**Supplementary Figure S2.** Volcano plot of differentially abundant proteins in the EtOH group compared to control. The threshold for significance was set at a 1.2-fold change in abundance with a p-value < 0.01. Proteins that were significantly altered are labeled on the plot. Upregulated proteins (fold change ≥ 1.2, p-value < 0.01) are shown in red, downregulated proteins (fold change ≤ 0.8, p-value < 0.01) are shown in blue, and non-significant proteins are shown in gray.

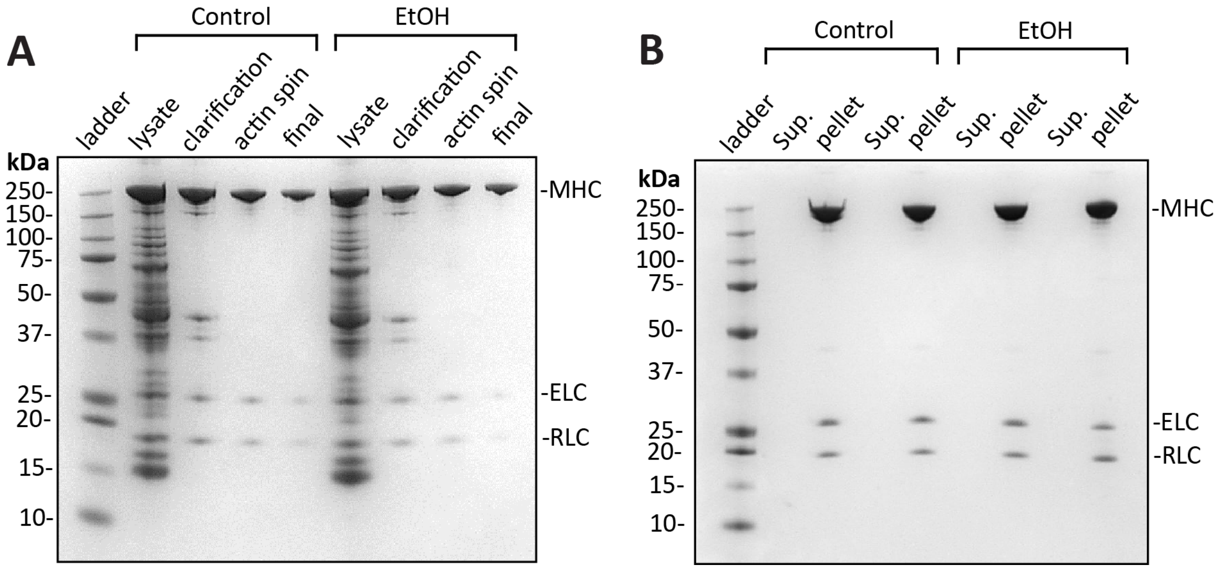


**Supplementary Figure S3.** Myosin Extraction and Synthetic Thick Filament Assembly. (A) Representative SDS-PAGE gel showing the steps of the myosin extraction protocol. Lanes 2 to 5 contain samples from control heart tissue, following lysis, ultracentrifugation, actin spin-down, and final dialysis. Lanes 6 to 10 represent the corresponding steps for the EtOH (alcohol-rich diet) group. (B) Representative SDS-PAGE gel showing synthetic thick filament assembly. Filamentary myosin was pelleted by centrifugation, and no detectable myosin was observed in the supernatant for either the control or EtOH groups.

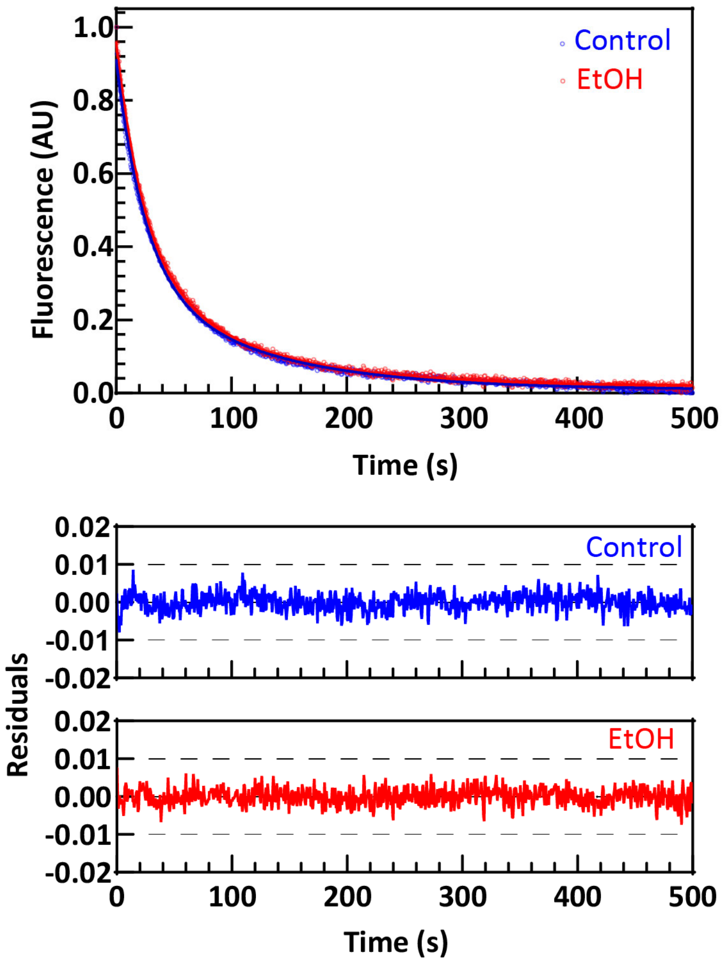


**Supplementary Figure S4.** Single ATP Turnover. Representative single mant-ATP turnover traces are presented. The data were fitted to a two-exponential function, with the fitting residuals shown for the control (blue) and EtOH (red) groups. The rate constants of fast phase (DRX) and slow phase (SRX) are 0.025 $s^{-1}$ and 0.0035 $s^{-1}$ in the control group, and 0.025 $s^{-1}$ and 0.0053 $s^{-1}$ in the EtOH group. And the relative amplitudes of slow component (SRX fraction) are 0.20 and 0.21 in the control group and EtOH group, respectively.

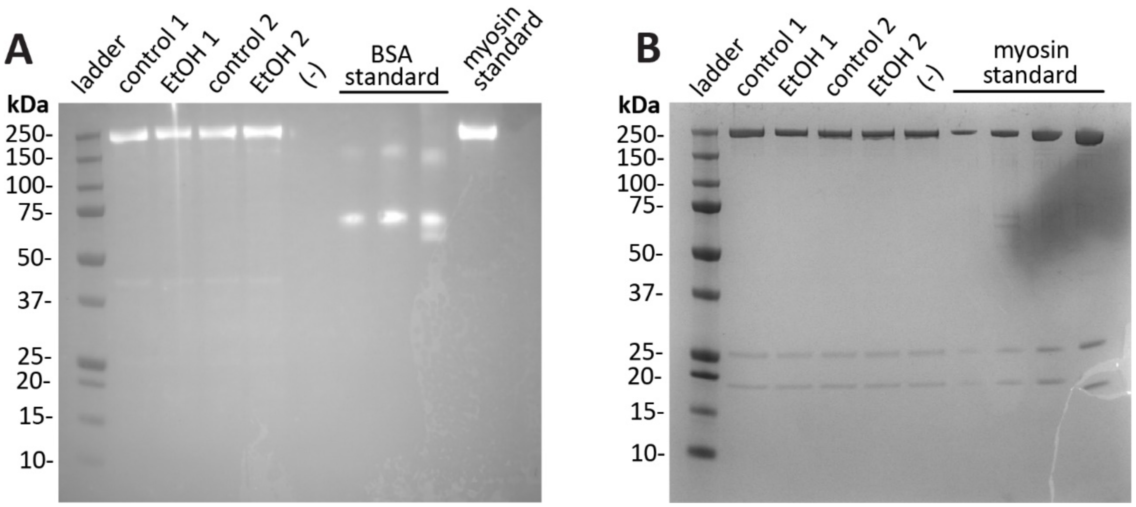


**Supplementary Figure S5.** Carbonylation Analysis. (A) Representative Western blot showing two separate myosin preparations from the control and EtOH groups. From left to right: molecular weight ladder, followed by myosin samples with DNPH derivatization of carbonyl compounds, and a negative control (-) without DNPH. Also shown are a carbonylated BSA standard and a myosin standard. (B) Representative SDS-PAGE gel, Coomassie stained, used to calculate the protein concentration of myosin samples, relative to a myosin standard of known concentration.

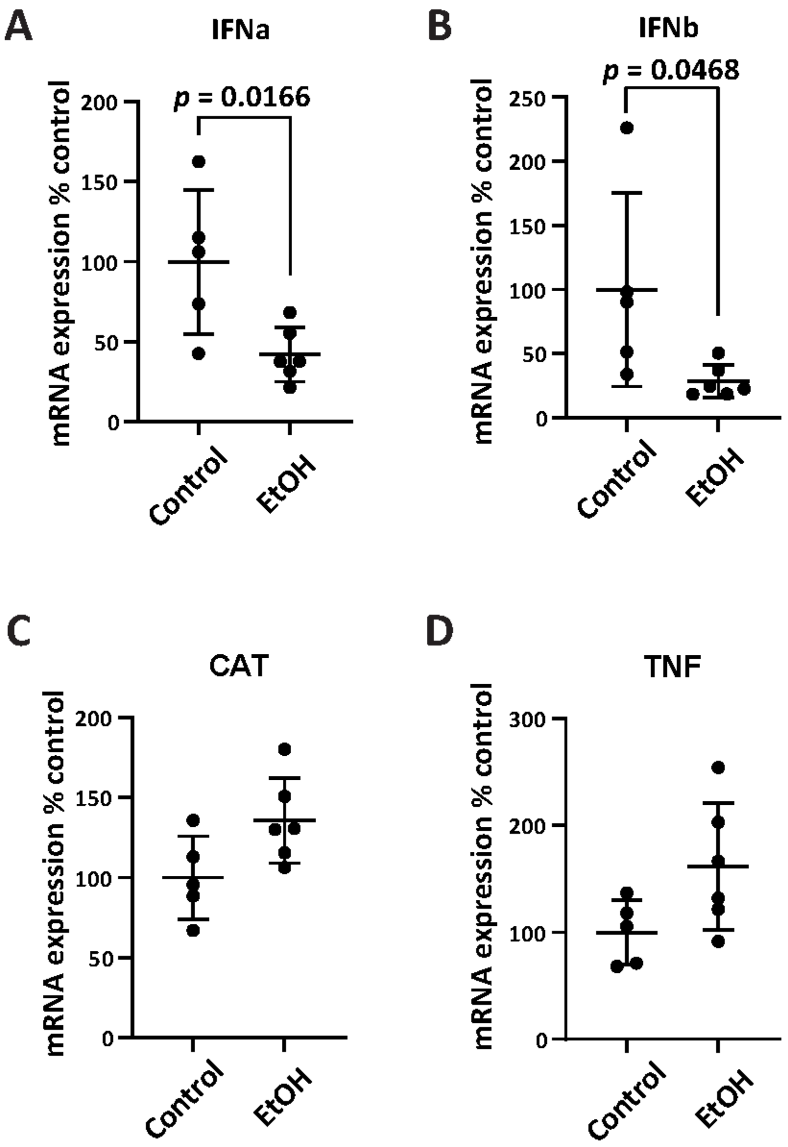


**Supplementary Figure S6**. mRNA Expression. Comparison of mRNA expression levels between the control and EtOH groups for (A) IFNα, (B) IFNβ, (C) CAT, and (D) TNF. Each solid dot represents the mean expression value for an individual animal (n = 5 for control, n = 6 for EtOH). The black line indicates the group mean ± SD. Statistical significance was determined using an unpaired t-test.

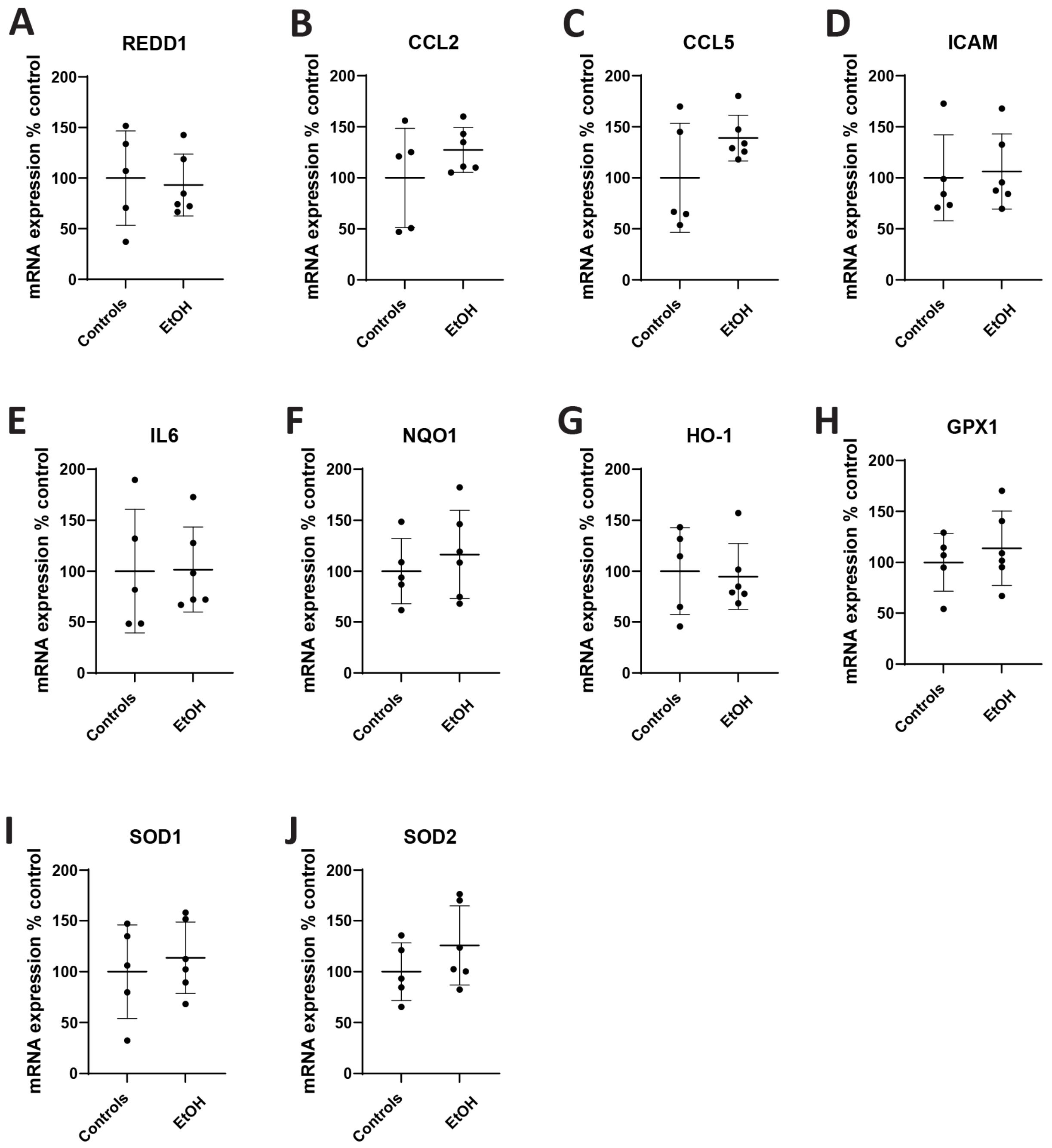


**Supplementary Figure S7.** mRNA Expression. Comparison of mRNA expression levels between the control and EtOH groups for (A) REDD1, (B) CCL2, (C) CCL5, (D) ICAM, (E) IL6, (F) NQO1, (G) HO-1, (H) GPX1, (I) SOD1, and (J) SOD2. Each solid dot represents the mean expression value for an individual animal (n = 5 for control, n = 6 for EtOH). The black line indicates the group mean ± SD.

Supplemental Table S2. **Summary of Mass Spectrometry Results**

| | | | Abundance (Top 3) | | SD of Abundance (Top 3) | | EtOH/Control | | T-test |
|---|---|---|---|---|---|---|---|---|---|
| **Gene** | **Master. Protein. Accessions** | **Peptides** | **EtOH** | **Control** | **EtOH** | **Control** | Ratio | SD of ratio | p_value |
| A1bg | Q9EPH1 | 4 | 7.97E-05 | 8.47E-05 | 6.31E-10 | 1.93E-09 | 0.9277 | 0.2830 | 9.98E-01 |
| A1i3 | P14046 | 4 | 2.80E-05 | 4.50E-05 | 9.33E-11 | 2.26E-10 | 0.6559 | 0.0920 | 5.09E-02 |
| A1m | Q63041 | 5 | 5.77E-05 | 7.82E-05 | 5.27E-10 | 9.38E-10 | 0.7404 | 0.1668 | 4.75E-02 |
| Ablim1; Ablim1 | F1LWK7; Q3KR72 | 2 | 1.39E-05 | 1.16E-05 | 1.59E-11 | 9.69E-12 | 1.2338 | 0.2310 | 1.69E-01 |
| Acaa2 | P13437 | 5 | 8.31E-04 | 5.56E-04 | 3.83E-08 | 1.22E-08 | 1.4164 | 0.1760 | 1.23E-05 |
| Acad9 | B1WC61 | 4 | 2.52E-05 | 2.42E-05 | 1.22E-10 | 7.40E-12 | 1.0403 | 0.0957 | 5.19E-01 |
| Acadl | P15650 | 5 | 1.10E-03 | 1.04E-03 | 2.50E-08 | 5.50E-08 | 1.0307 | 0.1344 | 6.49E-01 |
| Acadm | G3V796 | 5 | 1.93E-04 | 1.81E-04 | 6.34E-10 | 1.27E-09 | 1.0753 | 0.0530 | 1.10E-01 |
| Acads | Q6IMX3 | 5 | 1.63E-04 | 1.54E-04 | 1.22E-09 | 9.13E-10 | 1.0540 | 0.1082 | 4.90E-01 |
| Acadvl | Q5M9H2 | 5 | 1.94E-04 | 1.82E-04 | 4.74E-10 | 7.64E-10 | 1.0958 | 0.0516 | 2.29E-03 |
| Acat1 | P17764 | 5 | 5.11E-04 | 4.74E-04 | 2.61E-09 | 3.60E-09 | 1.0570 | 0.0351 | 2.31E-01 |
| Aco2 | Q9ER34 | 5 | 1.25E-03 | 1.15E-03 | 5.99E-08 | 2.67E-08 | 1.0176 | 0.1982 | 7.34E-01 |
| Acot13 | D3ZA93 | 2 | 9.02E-05 | 9.77E-05 | 3.64E-10 | 2.24E-10 | 0.9257 | 0.0749 | 6.34E-01 |
| Acot2 | O55171 | 5 | 2.40E-04 | 1.65E-04 | 1.92E-09 | 1.33E-09 | 1.4683 | 0.4256 | 6.47E-10 |
| Acp1 | Z4YNF4 | 2 | 2.14E-05 | 2.28E-05 | 1.32E-10 | 1.59E-11 | 0.8928 | 0.1764 | 7.89E-01 |
| Acsf2 | Q499N5 | 5 | 1.06E-04 | 9.13E-05 | 1.05E-09 | 1.34E-10 | 1.0481 | 0.1983 | 2.38E-01 |
| Acsl1 | P18163 | 5 | 1.75E-04 | 1.61E-04 | 4.18E-10 | 5.55E-10 | 1.0824 | 0.0409 | 6.07E-02 |
| Acss1 | D3ZZN3 | 5 | 4.98E-05 | 4.68E-05 | 2.05E-10 | 7.71E-11 | 1.0399 | 0.1231 | 8.11E-02 |
| Acta2; P68035 | B0BMT0; P68035 | 5 | 5.12E-03 | 5.32E-03 | 2.31E-06 | 1.25E-06 | 0.8722 | 0.3775 | 5.99E-01 |
| Actb | P60711 | 5 | 1.39E-04 | 1.46E-04 | 3.66E-09 | 1.67E-09 | 0.8398 | 0.3653 | 8.53E-01 |
| Actb; Acta2; P68035 | P60711; B0BMT0; P68035 | 5 | 5.23E-03 | 4.43E-03 | 2.97E-06 | 8.52E-07 | 1.1046 | 0.8748 | 7.48E-01 |
| Actbl2; Actb; Acta2; P68035 | D3ZRN3; P60711; B0BMT0; P68035 | 5 | 7.94E-03 | 7.91E-03 | 1.91E-06 | 1.12E-06 | 0.8938 | 0.2262 | 7.28E-01 |
| Actn1 | Q6T487 | 5 | 2.36E-05 | 2.34E-05 | 1.54E-10 | 6.63E-11 | 1.0352 | 4.3322 | 7.10E-01 |
| Actn1; Actn2 | Q6T487; D3ZCV0 | 5 | 3.56E-04 | 3.36E-04 | 4.41E-09 | 2.50E-09 | 1.0362 | 0.0581 | 8.12E-01 |
| Actn2 | D3ZCV0 | 5 | 3.95E-04 | 4.14E-04 | 7.97E-09 | 6.00E-09 | 0.9460 | 0.0660 | 3.44E-01 |
| Acyp2 | D4A1G1 | 3 | 1.94E-05 | 1.91E-05 | 2.23E-11 | 1.48E-11 | 0.9754 | 0.1057 | 3.20E-01 |
| Adck3 | Q5BJQ0 | 5 | 6.94E-05 | 5.34E-05 | 4.53E-10 | 1.83E-10 | 1.2293 | 0.1565 | 1.20E-02 |
| Adsl | D3ZW08 | 2 | 5.21E-06 | 6.49E-06 | 5.25E-12 | 2.08E-12 | 0.8405 | 0.1662 | 2.12E-02 |
| Adssl1 | M0R9J5 | 5 | 1.08E-04 | 1.02E-04 | 5.84E-10 | 4.59E-10 | 0.9347 | 0.2168 | 4.70E-01 |
| Afg3l2 | F1LN92 | 2 | 1.62E-05 | 1.55E-05 | 2.62E-12 | 2.84E-12 | 1.0446 | 0.0241 | 3.57E-01 |
| Agl | D4AEH9 | 5 | 2.43E-05 | 2.02E-05 | 3.21E-10 | 2.05E-11 | 1.0995 | 0.4019 | 9.45E-02 |
| Ahnak | M0R9D5 | 5 | 1.15E-04 | 1.02E-04 | 2.47E-10 | 3.45E-10 | 1.1312 | 0.1072 | 6.38E-01 |
| Ahsg | P24090 | 4 | 3.88E-05 | 4.26E-05 | 2.75E-10 | 1.93E-10 | 0.9265 | 0.2888 | 1.43E-01 |
| Aifm1 | Q9JM53 | 4 | 3.07E-05 | 2.77E-05 | 1.51E-11 | 1.17E-11 | 1.0596 | 0.0388 | 2.01E-02 |
| Ak1 | P39069 | 5 | 1.13E-04 | 1.30E-04 | 7.26E-09 | 2.41E-09 | 0.8689 | 0.2766 | 2.27E-01 |

| | | | | | | | | | |
|---|---|---|---|---|---|---|---|---|---|
| Ak2 | P29410 | 2 | 6.64E-05 | 6.15E-05 | 8.04E-11 | 6.91E-11 | 1.0669 | 0.0428 | 4.69E-01 |
| Ak3 | P29411 | 2 | 2.18E-05 | 1.77E-05 | 2.69E-10 | 2.33E-11 | 1.2290 | 0.7042 | 6.15E-02 |
| Ak4 | Q9WUS0 | 2 | 2.71E-05 | 2.31E-05 | 2.66E-11 | 8.33E-12 | 1.1514 | 0.0697 | 2.58E-01 |
| Akr1b1 | P07943 | 5 | 1.31E-04 | 1.35E-04 | 3.31E-09 | 2.19E-09 | 0.9092 | 0.2238 | 2.93E-01 |
| Akr1b10 | Q6AY99 | 3 | 1.42E-05 | 1.60E-05 | 3.00E-11 | 3.23E-11 | 0.8809 | 0.1931 | 1.71E-01 |
| Akr1cl | D3ZF77 | 2 | 3.17E-05 | 2.85E-05 | 1.75E-10 | 9.83E-11 | 1.1022 | 0.3586 | 4.71E-01 |
| Alb | P02770 | 5 | 4.04E-03 | 4.57E-03 | 2.25E-06 | 2.05E-06 | 0.8175 | 0.1675 | 7.66E-02 |
| Aldh1a1 | P51647 | 3 | 1.04E-05 | 9.21E-06 | 5.92E-11 | 2.36E-11 | 1.3048 | 2.5800 | 6.69E-02 |
| Aldh2; Aldh2 | P11884; F1LN88 | 5 | 1.35E-04 | 1.24E-04 | 3.68E-10 | 2.51E-10 | 1.1028 | 0.0541 | 9.82E-02 |
| Aldh6a1 | G3V7J0 | 5 | 2.17E-04 | 2.15E-04 | 1.14E-09 | 8.95E-10 | 1.0312 | 0.0699 | 2.63E-01 |
| Aldh7a1 | Q64057 | 4 | 1.71E-05 | 1.70E-05 | 1.02E-11 | 7.22E-12 | 0.9467 | 0.1848 | 3.17E-01 |
| Aldh9a1 | Q9JLJ3 | 5 | 6.42E-05 | 4.72E-05 | 7.93E-10 | 1.63E-10 | 1.1939 | 0.2148 | 9.12E-02 |
| Aldoa | P05065 | 5 | 6.77E-04 | 6.86E-04 | 2.15E-08 | 1.67E-08 | 0.9815 | 0.1058 | 5.43E-01 |
| Alpha II spectrin | C9EH87 | 5 | 6.72E-05 | 4.87E-05 | 5.86E-10 | 2.05E-10 | 1.3298 | 0.2402 | 2.31E-02 |
| Anxa11 | Q5XI77 | 3 | 2.20E-05 | 1.86E-05 | 3.11E-11 | 2.32E-11 | 1.1778 | 0.1724 | 2.39E-01 |
| Anxa2 | Q07936 | 4 | 1.61E-05 | 1.72E-05 | 4.03E-11 | 2.51E-11 | 0.9404 | 0.2212 | 8.05E-01 |
| Anxa5 | P14668 | 5 | 6.63E-05 | 7.11E-05 | 1.71E-10 | 2.37E-10 | 0.9548 | 0.1130 | 2.29E-01 |
| Anxa6 | P48037 | 5 | 4.56E-05 | 4.49E-05 | 8.28E-11 | 6.87E-11 | 1.0707 | 0.2665 | 7.60E-01 |
| Apoa1 | P04639 | 5 | 8.46E-05 | 9.51E-05 | 7.45E-10 | 1.33E-09 | 0.8453 | 0.2227 | 3.36E-01 |
| Apoa2 | P04638 | 2 | 8.50E-06 | 1.24E-05 | 3.14E-11 | 3.90E-11 | 0.6802 | 0.3248 | 3.27E-01 |
| Apoa4 | P02651 | 4 | 4.42E-05 | 4.90E-05 | 2.95E-10 | 1.07E-10 | 1.0047 | 0.2119 | 4.31E-01 |
| Apobec2 | B4F789 | 2 | 3.19E-05 | 3.37E-05 | 3.46E-11 | 2.81E-11 | 0.9345 | 0.0474 | 2.42E-01 |
| Apoo | M0R7G4 | 3 | 3.16E-05 | 2.67E-05 | 6.53E-11 | 2.49E-11 | 1.1539 | 0.1051 | 3.64E-01 |
| Apool | Q5U1W6 | 2 | 1.70E-05 | 1.68E-05 | 8.21E-12 | 7.62E-12 | 1.0511 | 0.0508 | 4.99E-01 |
| Aprt | P36972 | 4 | 3.53E-05 | 3.72E-05 | 2.21E-10 | 1.79E-10 | 0.9074 | 0.2153 | 7.18E-01 |
| Arf3 | P61206 | 3 | 1.93E-05 | 2.07E-05 | 8.11E-11 | 6.85E-11 | 0.9314 | 0.2911 | 5.42E-01 |
| Arhgdia | Q5XI73 | 2 | 2.73E-05 | 2.67E-05 | 2.97E-11 | 1.08E-11 | 1.0309 | 0.0646 | 4.43E-01 |
| Aspn | Q5XIH1 | 3 | 5.95E-06 | 8.71E-06 | 6.11E-11 | 5.08E-11 | 0.7481 | 3.5187 | 5.82E-01 |
| Atic | O35567 | 3 | 2.26E-05 | 2.20E-05 | 5.92E-11 | 2.83E-11 | 1.0301 | 0.2285 | 7.67E-01 |
| Atp12a; Atp1a1 | G3V8S4; P06685 | 3 | 5.34E-05 | 4.98E-05 | 8.54E-11 | 7.29E-11 | 1.0984 | 0.0556 | 6.37E-01 |
| Atp1a1 | P06685 | 5 | 7.79E-05 | 6.61E-05 | 1.25E-10 | 1.00E-10 | 1.1673 | 0.0578 | 6.89E-05 |
| Atp1b1 | P07340 | 3 | 5.43E-05 | 4.60E-05 | 1.06E-10 | 1.28E-10 | 1.1192 | 0.0755 | 5.99E-01 |
| Atp2a2 | P11507 | 5 | 5.25E-04 | 5.53E-04 | 6.02E-09 | 5.82E-09 | 0.9885 | 0.0430 | 3.94E-01 |
| Atp5a1 | F1LP05 | 5 | 2.66E-03 | 2.30E-03 | 8.44E-08 | 9.30E-08 | 1.1284 | 0.0383 | 5.74E-04 |
| Atp5b | G3V6D3 | 5 | 2.72E-03 | 2.45E-03 | 9.81E-08 | 7.75E-08 | 1.1009 | 0.0277 | 2.51E-02 |
| Atp5d | G3V7Y3 | 3 | 3.83E-04 | 3.47E-04 | 2.38E-09 | 2.01E-09 | 0.9904 | 0.0383 | 9.84E-01 |
| Atp5e | P29418 | 3 | 1.60E-04 | 1.40E-04 | 2.60E-10 | 2.93E-10 | 1.1471 | 0.0343 | 1.52E-01 |
| Atp5f1 | P19511 | 5 | 4.62E-04 | 4.30E-04 | 3.01E-09 | 3.00E-09 | 0.9536 | 0.2157 | 9.06E-01 |
| Atp5h | P31399 | 5 | 5.28E-04 | 5.06E-04 | 8.40E-09 | 7.32E-09 | 1.0262 | 0.0611 | 5.45E-01 |
| Atp5i | P29419 | 4 | 3.78E-04 | 3.39E-04 | 2.19E-09 | 2.09E-09 | 1.0228 | 0.0449 | 8.61E-01 |
| Atp5j | P21571 | 4 | 1.37E-04 | 1.22E-04 | 1.54E-09 | 9.21E-10 | 1.1444 | 1.0395 | 7.14E-01 |
| Atp5j2 | D3ZAF6 | 4 | 3.08E-04 | 2.75E-04 | 3.28E-09 | 3.99E-09 | 1.2784 | 5.6127 | 4.50E-01 |
| Atp5l | Q6PDU7 | 4 | 7.57E-05 | 6.75E-05 | 8.74E-10 | 3.97E-10 | 0.8903 | 0.8335 | 7.46E-01 |
| Atp5o | Q06647 | 5 | 7.78E-04 | 7.05E-04 | 5.82E-09 | 1.03E-08 | 1.1113 | 0.0747 | 2.06E-01 |
| ATP8 | Q5UAJ5 | 2 | 2.57E-04 | 1.94E-04 | 9.02E-09 | 1.71E-09 | 1.3721 | 0.5243 | 2.81E-03 |
| Auh | F1LU71 | 5 | 3.01E-05 | 2.77E-05 | 4.53E-11 | 2.59E-11 | 1.0264 | 0.0825 | 2.44E-01 |
| Bcat2 | G3V8U8 | 5 | 6.49E-05 | 5.94E-05 | 5.12E-10 | 1.18E-10 | 0.9482 | 0.1750 | 8.65E-01 |

| | | | | | | | | | |
|---|---|---|---|---|---|---|---|---|---|
| Bcl-2-interacting death suppressor | Q156J1 | 2 | 2.07E-05 | 2.09E-05 | 1.38E-11 | 1.66E-11 | 0.9948 | 0.0627 | 6.43E-01 |
| Bdh1 | P29147 | 3 | 6.07E-05 | 5.81E-05 | 3.55E-09 | 7.88E-10 | 0.8336 | 0.5567 | 5.52E-01 |
| Bgn | P47853 | 2 | 3.91E-05 | 1.07E-05 | 1.15E-10 | 7.52E-11 | 2.8560 | 3.0059 | 8.36E-01 |
| Blvrb | B5DF65 | 3 | 1.97E-05 | 2.19E-05 | 1.89E-10 | 4.48E-11 | 0.8715 | 0.3087 | 1.58E-01 |
| Bsg | P26453 | 4 | 3.61E-05 | 4.18E-05 | 1.11E-09 | 5.99E-10 | 0.9646 | 0.3370 | 7.88E-01 |
| C1qbp | O35796 | 3 | 4.43E-05 | 4.35E-05 | 4.04E-11 | 8.27E-11 | 1.0065 | 0.0560 | 6.87E-01 |
| C3 | M0RBF1 | 5 | 7.87E-05 | 8.12E-05 | 3.78E-10 | 1.52E-10 | 0.8532 | 0.1641 | 1.26E-01 |
| Ca1 | B0BNN3 | 5 | 3.61E-05 | 4.07E-05 | 1.54E-10 | 3.34E-10 | 0.8537 | 0.2295 | 2.82E-01 |
| Ca2 | P27139 | 5 | 5.00E-05 | 5.35E-05 | 2.61E-10 | 7.27E-10 | 0.8542 | 0.4352 | 3.34E-01 |
| Calm1 | P62161 | 2 | 4.21E-05 | 4.00E-05 | 7.58E-11 | 1.17E-10 | 1.0338 | 0.0682 | 9.65E-01 |
| Canx | P35565 | 2 | 1.38E-05 | 1.36E-05 | 1.10E-11 | 6.95E-12 | 1.0293 | 0.1687 | 7.75E-01 |
| Capza2 | Q3T1K5 | 2 | 1.36E-05 | 1.51E-05 | 1.18E-11 | 6.63E-12 | 0.8943 | 0.0777 | 4.86E-01 |
| Cardiac titin N2B isoform (Fragment) | Q7TMZ9 | 5 | 8.79E-05 | 9.26E-05 | 5.87E-10 | 4.03E-10 | 0.9693 | 0.1109 | 5.95E-01 |
| Casq2 | F1M944 | 5 | 2.94E-04 | 2.82E-04 | 1.74E-09 | 7.24E-10 | 1.0290 | 0.0263 | 3.18E-01 |
| Cat | P04762 | 5 | 1.82E-05 | 1.24E-05 | 1.41E-11 | 3.73E-12 | 1.5054 | 0.1665 | 2.18E-06 |
| Cav1 | P41350 | 3 | 3.77E-05 | 4.23E-05 | 6.21E-11 | 2.57E-10 | 0.8906 | 0.1159 | 1.87E-01 |
| Cav3; Cav1 | P51638; P41350 | 2 | 4.06E-05 | 3.79E-05 | 1.43E-10 | 8.01E-11 | 1.0595 | 0.2955 | 9.27E-01 |
| Cbr1 | M0R3X6 | 4 | 4.17E-05 | 2.64E-05 | 1.23E-10 | 3.71E-11 | 1.3676 | 0.6756 | 2.07E-01 |
| CD36/FAT | O35754 | 5 | 1.10E-04 | 9.74E-05 | 4.11E-10 | 3.14E-10 | 1.0501 | 0.1466 | 7.42E-01 |
| Cdh2 | G3V803 | 3 | 1.67E-05 | 1.52E-05 | 1.25E-11 | 1.36E-11 | 1.0833 | 0.1419 | 4.96E-01 |
| Cfl2 | M0RC65 | 2 | 1.51E-05 | 1.58E-05 | 7.03E-11 | 6.08E-11 | 0.9087 | 3.2458 | 6.30E-01 |
| Chchd3 | D3ZUX5 | 5 | 9.15E-05 | 7.87E-05 | 1.31E-10 | 9.09E-11 | 1.1005 | 0.0497 | 3.50E-01 |
| Cilp2 | D3ZE05 | 2 | 1.32E-05 | 2.31E-05 | NA | 2.73E-10 | 0.6638 | 0.0000 | 8.09E-01 |
| Cisd1 | B0K020 | 3 | 2.12E-05 | 1.50E-05 | 1.20E-10 | 1.95E-10 | 3.4791 | 586.1979 | 9.68E-01 |
| Ckb | P07335 | 5 | 5.16E-05 | 7.97E-05 | 2.10E-10 | 7.17E-10 | 0.6767 | 0.0841 | 3.18E-04 |
| Ckm | P00564 | 5 | 1.03E-03 | 1.05E-03 | 4.23E-08 | 3.33E-08 | 0.9661 | 0.0776 | 1.78E-01 |
| Ckmt2 | B0BNC0 | 5 | 1.88E-03 | 1.87E-03 | 8.64E-08 | 1.44E-07 | 1.0167 | 0.0723 | 5.56E-01 |
| Cltc | F1M779 | 3 | 1.31E-05 | 1.39E-05 | 1.75E-11 | 1.97E-11 | 0.9324 | 0.1674 | 3.32E-01 |
| Col15a1 | F1LPD0 | 2 | 1.21E-05 | 1.12E-05 | 2.10E-11 | 3.52E-11 | 1.0813 | 0.3884 | 5.40E-01 |
| Col1a1 | P02454 | 5 | 4.25E-04 | 4.75E-04 | 3.87E-07 | 1.31E-07 | 0.9174 | 2.3245 | 5.98E-01 |
| Col1a1; Col2a1 | P02454; F1LRM7 | 2 | 2.07E-04 | 2.51E-04 | 1.05E-07 | 6.10E-08 | 0.8815 | 2.6322 | 9.73E-01 |
| Col1a2 | F1LS40 | 5 | 2.42E-04 | 2.56E-04 | 9.70E-08 | 3.26E-08 | 1.0612 | 2.1149 | 5.61E-01 |
| Col2a1 | F1LRM7 | 2 | 3.73E-05 | 2.85E-05 | 6.08E-09 | 5.73E-10 | 1.3796 | 7.1401 | 5.21E-02 |
| Col3a1 | P13941 | 5 | 5.61E-04 | 6.16E-04 | 2.66E-07 | 1.32E-07 | 0.9453 | 0.9268 | 7.16E-01 |
| Col4a1 | F1MA59 | 3 | 4.19E-05 | 3.61E-05 | 2.49E-10 | 1.14E-10 | 1.1598 | 0.3073 | 2.72E-01 |
| Col4a2 | F1M6Q3 | 5 | 3.31E-05 | 2.80E-05 | 8.98E-11 | 4.07E-11 | 1.2301 | 0.2791 | 1.43E-01 |
| Col5a1 | Q9JI03 | 2 | 1.33E-05 | 1.47E-05 | 1.03E-10 | 8.87E-11 | 0.9458 | 0.8468 | 8.32E-01 |
| Col5a2 | F1LQ00 | 3 | 4.68E-05 | 5.68E-05 | 4.68E-09 | 2.13E-09 | 0.8984 | 0.8397 | 5.69E-01 |
| Col6a1 | D3ZUL3 | 5 | 6.92E-05 | 6.36E-05 | 8.06E-10 | 1.11E-09 | 1.0614 | 0.4614 | 7.85E-01 |
| Col6a2 | F1LNH3 | 5 | 1.50E-04 | 9.74E-05 | 1.13E-08 | 5.35E-09 | 1.1701 | 0.7955 | 3.70E-01 |

| | | | | | | | | | |
|---|---|---|---|---|---|---|---|---|---|
| Col6a3 | D4A115 | 5 | 6.94E-05 | 5.45E-05 | 3.65E-10 | 4.69E-10 | 1.1989 | 0.3945 | 3.76E-02 |
| Coq9 | Q68FT1 | 4 | 5.05E-05 | 4.86E-05 | 6.02E-11 | 8.36E-11 | 0.7304 | 1.2212 | 6.35E-01 |
| COX1 | A0A0A1FZ 34 | 2 | 1.93E-05 | 2.59E-05 | 1.86E-10 | 8.69E-11 | 0.6654 | 0.2625 | 1.96E-01 |
| COX2; COX2 | A0A097PE 04; Q5UAJ6 | 4 | 6.18E-04 | 5.76E-04 | 9.26E-09 | 1.06E-08 | 0.9888 | 0.3578 | 9.93E-01 |
| Cox4i1 | P10888 | 5 | 8.05E-04 | 7.38E-04 | 2.00E-08 | 1.45E-08 | 1.1169 | 0.1148 | 5.94E-02 |
| Cox5a | P11240 | 5 | 6.16E-04 | 5.32E-04 | 5.80E-09 | 6.99E-09 | 1.0975 | 0.1440 | 6.10E-01 |
| Cox5b | P12075 | 5 | 2.48E-04 | 2.54E-04 | 4.72E-09 | 3.70E-09 | 0.9090 | 1.4680 | 8.95E-01 |
| Cox6b1 | D3ZD09 | 5 | 6.20E-04 | 5.73E-04 | 5.27E-09 | 2.58E-08 | 1.0202 | 0.0589 | 6.21E-01 |
| Cox6c2 | P11951 | 3 | 3.65E-04 | 3.50E-04 | 4.73E-09 | 4.63E-09 | 1.0423 | 0.1355 | 6.02E-01 |
| Cox7a2l | D3ZYX8 | 2 | 5.25E-05 | 4.98E-05 | 5.95E-11 | 3.07E-11 | 1.0528 | 0.0374 | 7.75E-02 |
| Cox7b | P80431 | 2 | 1.15E-04 | 1.35E-04 | 7.61E-10 | 4.20E-10 | 0.8860 | 0.0456 | 5.80E-01 |
| Cox7c | P80432 | 2 | 1.26E-04 | 1.24E-04 | 2.44E-09 | 9.37E-10 | 1.0123 | 0.3425 | 7.09E-01 |
| Cp | G3V7K3 | 5 | 3.46E-05 | 3.07E-05 | 2.03E-10 | 1.69E-10 | 1.0591 | 0.5072 | 5.77E-01 |
| Cpt1b | Q63704 | 5 | 8.61E-05 | 8.36E-05 | 2.12E-10 | 1.53E-10 | 1.0225 | 0.0760 | 6.78E-01 |
| Cpt2 | P18886 | 5 | 8.10E-05 | 6.51E-05 | 1.46E-10 | 5.24E-11 | 1.1813 | 0.0563 | 2.80E-04 |
| Crat | Q704S8 | 5 | 5.98E-05 | 6.29E-05 | 2.04E-10 | 1.29E-10 | 0.9563 | 0.0891 | 8.69E-01 |
| Crip2 | P36201 | 2 | 3.83E-05 | 4.20E-05 | 7.70E-11 | 1.66E-10 | 0.9087 | 0.1464 | 1.17E-01 |
| Cryab | P23928 | 5 | 7.40E-04 | 8.00E-04 | 2.13E-08 | 2.75E-08 | 0.9291 | 0.0616 | 3.85E-01 |
| Cs | G3V936 | 5 | 1.11E-03 | 8.66E-04 | 5.07E-08 | 1.80E-08 | 1.2097 | 0.1025 | 6.45E-03 |
| Csrp3 | G3V7U0 | 5 | 8.76E-05 | 1.36E-04 | 9.48E-10 | 9.74E-10 | 0.7421 | 0.0903 | 6.70E-03 |
| Ctsd | P24268 | 3 | 4.21E-05 | 3.86E-05 | 1.66E-10 | 5.39E-11 | 1.0820 | 0.3243 | 3.40E-01 |
| Cyb5r3 | P20070 | 2 | 2.05E-05 | 1.95E-05 | 1.50E-11 | 1.39E-11 | 1.0410 | 0.0683 | 8.81E-01 |
| Dbi | Q6TXF3 | 2 | 2.67E-05 | 2.96E-05 | 3.88E-11 | 4.45E-11 | 0.7716 | 0.0820 | 2.55E-01 |
| Dbt | B2GV15 | 5 | 2.25E-05 | 1.94E-05 | 2.04E-11 | 1.93E-11 | 1.0444 | 0.1214 | 1.95E-01 |
| Dcn | Q01129 | 5 | 6.67E-05 | 6.13E-05 | 1.76E-09 | 1.50E-09 | 1.0626 | 0.6927 | 9.51E-01 |
| Ddt | P80254 | 4 | 3.98E-05 | 4.33E-05 | 2.85E-10 | 1.92E-10 | 1.0479 | 0.7710 | 3.14E-01 |
| Decr1 | G3V734 | 5 | 1.90E-04 | 1.50E-04 | 2.24E-09 | 8.31E-10 | 1.3817 | 0.3146 | 6.78E-03 |
| Des | Q6P725 | 5 | 3.75E-04 | 3.53E-04 | 2.76E-09 | 3.47E-09 | 1.0956 | 0.0514 | 7.65E-02 |
| Dhrs4 | Q8VID1 | 5 | 4.71E-05 | 4.11E-05 | 2.37E-11 | 4.43E-11 | 1.1687 | 0.0645 | 4.08E-03 |
| Dlat | P08461 | 5 | 3.17E-04 | 2.82E-04 | 1.68E-09 | 1.70E-09 | 1.1302 | 0.0580 | 3.19E-02 |
| Dld | Q6P6R2 | 5 | 2.82E-04 | 2.60E-04 | 2.65E-09 | 1.24E-09 | 1.0490 | 0.0501 | 3.86E-01 |
| Dlst | G3V6P2 | 5 | 3.18E-04 | 2.93E-04 | 7.79E-10 | 1.35E-09 | 1.0601 | 0.0321 | 2.52E-01 |
| Dmd | F1M705 | 2 | 2.20E-05 | 1.81E-05 | 4.03E-11 | 1.47E-11 | 1.1349 | 0.1152 | 7.68E-01 |
| Dnm1l | O35303 | 2 | 1.44E-05 | 1.33E-05 | 3.13E-11 | 7.64E-12 | 1.0492 | 0.1172 | 7.85E-01 |
| Dpysl2 | P47942 | 3 | 2.41E-05 | 2.43E-05 | 4.64E-11 | 6.60E-11 | 0.9407 | 0.1763 | 5.53E-01 |
| Dsp | F1LMV6 | 5 | 2.26E-05 | 2.12E-05 | 2.25E-11 | 1.17E-11 | 1.0576 | 0.0768 | 8.17E-01 |
| Dstn | Q7M0E3 | 2 | 1.97E-05 | 7.22E-06 | 1.93E-10 | 2.03E-11 | 10.6129 | 59.1903 | 2.15E-01 |
| Ech1 | Q62651 | 5 | 2.04E-04 | 1.70E-04 | 9.15E-10 | 5.47E-10 | 1.1991 | 0.0779 | 2.53E-01 |
| Echs1 | P14604 | 5 | 3.92E-04 | 3.51E-04 | 7.30E-09 | 6.32E-09 | 1.0559 | 0.1205 | 7.12E-01 |
| Eci1 | P23965 | 5 | 3.63E-04 | 3.01E-04 | 3.02E-09 | 3.26E-09 | 1.1662 | 0.0858 | 4.08E-02 |
| Eci2 | Q5XIC0 | 5 | 5.92E-05 | 5.47E-05 | 7.20E-11 | 6.44E-11 | 1.0739 | 0.0909 | 2.72E-01 |
| Eef1a2 | P62632 | 5 | 8.77E-05 | 1.05E-04 | 2.12E-09 | 2.15E-09 | 0.8030 | 0.2538 | 9.05E-02 |
| Eef2 | P05197 | 5 | 5.06E-05 | 5.10E-05 | 2.01E-10 | 7.05E-11 | 0.9624 | 0.0829 | 4.02E-01 |
| Ehd1 | Q641Z6 | 2 | 1.50E-05 | 1.61E-05 | 1.68E-11 | 9.62E-12 | 0.8856 | 0.0896 | 6.88E-01 |
| Ehd2 | Q4V8H8 | 5 | 3.65E-05 | 3.25E-05 | 6.16E-11 | 3.78E-11 | 1.1466 | 0.0947 | 1.45E-03 |
| Ehd4 | Q8R3Z7 | 3 | 1.66E-05 | 1.66E-05 | 9.33E-12 | 3.85E-12 | 1.0090 | 0.0503 | 8.26E-01 |
| Eif4a2 | Q5RKI1 | 2 | 1.47E-05 | 1.57E-05 | 1.43E-11 | 9.25E-12 | 0.9512 | 0.0837 | 9.38E-01 |
| Eif5a | Q3T1J1 | 3 | 3.42E-05 | 3.44E-05 | 1.62E-10 | 2.24E-10 | 0.9713 | 0.2276 | 7.70E-01 |

| | | | | | | | | | |
|---|---|---|---|---|---|---|---|---|---|
| Eno2 | P07323 | 2 | 3.85E-05 | 4.82E-05 | 7.23E-11 | 5.92E-11 | 0.8144 | 0.1019 | 2.35E-01 |
| Eno3 | P15429 | 5 | 6.75E-05 | 9.93E-05 | 1.71E-09 | 6.16E-10 | 0.6765 | 0.1778 | 4.64E-03 |
| Ephx2 | P80299 | 2 | 8.17E-06 | 1.06E-05 | 1.89E-11 | 1.86E-11 | 0.7705 | 0.2683 | 2.48E-01 |
| ES1 protein homolog, mitochondrial | P56571 | 5 | 2.96E-04 | 2.79E-04 | 2.51E-09 | 1.80E-09 | 1.1000 | 0.0617 | 4.50E-02 |
| Etfa | P13803 | 5 | 3.32E-04 | 2.91E-04 | 2.05E-09 | 2.34E-09 | 1.1306 | 0.0524 | 1.89E-02 |
| Etfb | Q68FU3 | 5 | 3.94E-04 | 3.43E-04 | 3.92E-09 | 3.59E-09 | 1.1596 | 0.0873 | 1.56E-05 |
| Etfdh | Q66HF3 | 5 | 1.56E-04 | 1.33E-04 | 2.72E-10 | 3.20E-10 | 1.1926 | 0.0388 | 4.05E-07 |
| Fabp3 | P07483 | 5 | 2.97E-03 | 2.70E-03 | 2.22E-06 | 6.95E-07 | 1.0749 | 0.3761 | 6.01E-01 |
| Fabp4 | Q9R290 | 5 | 3.15E-04 | 2.61E-04 | 3.10E-08 | 3.50E-09 | 1.1322 | 0.3794 | 1.81E-01 |
| Fahd2 | B2RYW9 | 3 | 1.85E-05 | 1.68E-05 | 1.16E-11 | 5.23E-12 | 1.0875 | 0.0563 | 3.10E-01 |
| Fam162a | Q4QQV3 | 5 | 6.10E-05 | 4.84E-05 | 4.00E-10 | 1.81E-10 | 1.2102 | 0.2968 | 2.07E-01 |
| Fam65b | Q7TP54 | 4 | 2.74E-05 | 2.39E-05 | 1.59E-10 | 3.19E-11 | 0.9827 | 0.3863 | 3.98E-02 |
| Fbn1 | G3V9M6 | 5 | 5.33E-05 | 4.65E-05 | 8.02E-10 | 4.76E-10 | 0.9866 | 1.7055 | 3.52E-01 |
| Fga | P06399 | 4 | 2.89E-05 | 3.13E-05 | 2.63E-10 | 2.76E-10 | 0.8920 | 0.3083 | 6.87E-01 |
| Fgb | P14480 | 2 | 2.14E-05 | 2.63E-05 | 9.52E-11 | 1.59E-10 | 0.8073 | 0.1984 | 2.25E-01 |
| Fgg | P02680 | 3 | 1.63E-05 | 2.04E-05 | 3.14E-11 | 5.52E-11 | 0.7980 | 0.1519 | 9.47E-02 |
| Fh | P14408 | 5 | 1.63E-04 | 1.47E-04 | 9.38E-10 | 4.99E-10 | 1.1320 | 0.0600 | 6.52E-03 |
| Fhl2 | O35115 | 5 | 6.03E-05 | 8.50E-05 | 2.05E-09 | 1.38E-09 | 0.6811 | 0.3022 | 1.37E-01 |
| Flna | C0JPT7 | 5 | 1.11E-05 | 7.50E-06 | 4.87E-10 | 3.68E-11 | 1.4617 | 13.1979 | 4.52E-02 |
| Flnc | D3ZHA0 | 5 | 1.81E-05 | 1.72E-05 | 1.58E-11 | 1.35E-11 | 1.0172 | 0.0963 | 6.14E-01 |
| Flnc; Flna | D3ZHA0; C0JPT7 | 2 | 8.47E-06 | 9.95E-06 | 5.48E-11 | 9.66E-12 | 0.8373 | 0.8347 | 6.89E-01 |
| Fth1 | Q5FVS1 | 3 | 8.05E-05 | 9.14E-05 | 6.86E-10 | 5.33E-10 | 0.8772 | 0.1329 | 1.05E-01 |
| Gapdh | P04797 | 5 | 8.80E-04 | 8.80E-04 | 6.99E-08 | 2.73E-08 | 1.0006 | 0.2506 | 9.08E-01 |
| Gbas | Q5RK08 | 5 | 5.19E-04 | 5.15E-04 | 1.02E-07 | 5.73E-08 | 1.0246 | 0.2389 | 8.50E-01 |
| Gc | P04276 | 2 | 2.50E-05 | 2.20E-05 | 3.12E-11 | 1.98E-11 | 0.9808 | 0.7078 | 4.23E-01 |
| Gdi2 | P50399 | 3 | 1.53E-05 | 1.52E-05 | 1.14E-11 | 5.36E-12 | 0.9571 | 0.0862 | 8.35E-01 |
| Glud1 | P10860 | 5 | 5.02E-05 | 5.56E-05 | 1.68E-10 | 1.46E-10 | 0.9095 | 0.0736 | 2.99E-01 |
| Glyceraldehyde-3-phosphate dehydrogenase; Gapdh | D3ZEN2; P04797 | 2 | 9.65E-04 | 9.75E-04 | 6.79E-08 | 4.72E-08 | 0.9943 | 0.1284 | 8.17E-01 |
| Gnb4 | O35353 | 2 | 2.30E-05 | 1.89E-05 | 5.40E-11 | 1.33E-11 | 1.1765 | 0.1463 | 3.09E-01 |
| Got1 | P13221 | 5 | 3.12E-04 | 3.11E-04 | 8.80E-09 | 4.09E-09 | 0.9889 | 0.1008 | 6.51E-01 |
| Got2 | P00507 | 5 | 8.89E-04 | 7.69E-04 | 4.44E-08 | 1.48E-08 | 1.1587 | 0.1313 | 6.77E-04 |
| Gpi | Q6P6V0 | 5 | 1.01E-04 | 1.04E-04 | 2.00E-09 | 1.03E-09 | 0.9510 | 0.2215 | 2.23E-01 |
| Gpx1 | P04041 | 5 | 5.88E-05 | 6.12E-05 | 9.75E-11 | 1.61E-10 | 0.9618 | 0.2104 | 8.39E-01 |
| Grpel1 | P97576 | 3 | 2.60E-05 | 2.24E-05 | 2.05E-11 | 1.79E-11 | 1.1572 | 0.0873 | 4.97E-02 |
| Grtp1 | Q6TXE7 | 2 | 1.60E-05 | 1.35E-05 | 1.87E-11 | 2.55E-11 | 1.2130 | 0.4850 | 7.17E-01 |
| Gsn | Q68FP1 | 2 | 1.33E-05 | 1.53E-05 | 1.84E-11 | 2.68E-11 | 0.8751 | 0.1857 | 2.86E-01 |
| Gstk1 | P24473 | 2 | 1.16E-05 | 1.20E-05 | 2.92E-12 | 4.88E-12 | 0.9718 | 0.0463 | 4.39E-01 |
| Gstm2 | P08010 | 5 | 6.43E-05 | 7.41E-05 | 3.07E-10 | 3.87E-10 | 0.8888 | 1.3655 | 8.96E-01 |
| Gstm2; Gstm3 | P08010; P08009 | 5 | 1.51E-04 | 1.46E-04 | 1.39E-09 | 1.21E-09 | 0.9977 | 0.1034 | 8.18E-01 |

| | | | | | | | | | |
|---|---|---|---|---|---|---|---|---|---|
| Gstp1 | P04906 | 3 | 2.29E-05 | 2.32E-05 | 1.68E-10 | 6.74E-11 | 1.0417 | 0.1751 | 7.37E-01 |
| H1f0 | P43278 | 2 | 4.23E-05 | 4.13E-05 | 7.36E-11 | 1.45E-10 | 1.0329 | 0.1147 | 5.29E-01 |
| H2afv | D4AEC0 | 2 | 1.49E-05 | 1.58E-05 | 6.42E-12 | 2.29E-11 | 0.9530 | 0.1225 | 6.38E-01 |
| H2afv; H2afx; Histone H2A type 1 | D4AEC0; D3ZXP3; P02262 | 2 | 8.32E-04 | 8.43E-04 | 2.41E-08 | 4.35E-08 | 0.9211 | 0.0814 | 8.97E-01 |
| H2afx | D3ZXP3 | 2 | 2.88E-05 | 2.46E-05 | 2.75E-11 | 3.82E-11 | 1.1899 | 0.1207 | 2.55E-01 |
| Hadh | Q9WVK7 | 5 | 4.19E-04 | 3.35E-04 | 4.76E-09 | 3.97E-09 | 1.2762 | 0.1012 | 6.20E-04 |
| Hadha | Q64428 | 5 | 4.74E-04 | 3.81E-04 | 4.49E-09 | 4.89E-09 | 1.2679 | 0.0908 | 9.55E-07 |
| Hadhb | Q60587 | 5 | 4.33E-04 | 3.49E-04 | 5.69E-09 | 4.39E-09 | 1.2563 | 0.0998 | 1.86E-07 |
| Hba1 | P01946 | 5 | 4.86E-03 | 6.34E-03 | 2.02E-06 | 9.48E-06 | 0.8426 | 0.2880 | 2.16E-01 |
| Hba1; LOC287167 | P01946; Q63910 | 2 | 2.76E-03 | 3.26E-03 | 1.12E-06 | 4.72E-06 | 1.0289 | 0.5048 | 7.79E-01 |
| Hbb | P02091 | 4 | 2.35E-03 | 2.78E-03 | 1.04E-06 | 3.97E-06 | 0.8457 | 0.3562 | 5.76E-01 |
| Hbb; Hemoglobin subunit beta-2 | P02091; P11517 | 5 | 3.11E-03 | 3.79E-03 | 8.38E-07 | 4.29E-06 | 0.7993 | 0.2543 | 1.56E-01 |
| Hbb; Zero beta-globin (Fragment); Hemoglobin subunit beta-2; Hbb-b1 | P02091; Q63011; P11517; Q62669 | 3 | 3.32E-03 | 3.95E-03 | 1.03E-06 | 5.42E-06 | 0.8802 | 0.2548 | 8.56E-01 |
| Hbe1; Hbb; Hemoglobin subunit beta-2 | O88752; P02091; P11517 | 2 | 3.96E-03 | 3.94E-03 | 1.35E-05 | 1.96E-05 | 0.9913 | 0.7786 | 9.26E-01 |
| Hemoglobin subunit beta-2 | P11517 | 2 | 9.39E-04 | 9.00E-04 | 1.09E-07 | 4.53E-07 | 0.9827 | 0.4102 | 7.81E-01 |
| Hhatl | D4A9P9 | 4 | 2.77E-05 | 2.70E-05 | 4.00E-11 | 7.41E-11 | 1.0132 | 0.1046 | 6.45E-01 |
| Hibadh | P29266 | 5 | 5.40E-05 | 5.05E-05 | 7.15E-11 | 6.21E-11 | 1.0673 | 0.0867 | 2.50E-01 |
| Hibch | Q5XIE6 | 3 | 1.18E-05 | 1.18E-05 | 1.27E-11 | 1.41E-11 | 1.0104 | 0.2104 | 9.26E-01 |
| Hint2 | D4AB01 | 2 | 2.12E-05 | 1.99E-05 | 1.12E-11 | 7.64E-12 | 1.0420 | 0.0591 | 8.88E-01 |
| Hist1h2bo | D3ZNH4 | 5 | 4.45E-04 | 4.31E-04 | 6.52E-09 | 1.32E-08 | 1.0092 | 0.0868 | 6.77E-01 |
| Hist1h4b | P62804 | 5 | 6.70E-04 | 5.94E-04 | 2.28E-08 | 2.52E-08 | 0.9817 | 0.1599 | 9.94E-01 |
| Hist2h3c2 | D3ZJ08 | 3 | 4.15E-04 | 3.89E-04 | 5.75E-09 | 1.03E-08 | 1.2027 | 0.2619 | 7.11E-01 |
| Histone H2A type 1 | P02262 | 3 | 3.24E-04 | 3.18E-04 | 6.65E-09 | 1.73E-08 | 1.0583 | 0.0924 | 7.12E-01 |
| Hk1 | P05708 | 5 | 2.86E-05 | 2.62E-05 | 3.02E-11 | 1.07E-11 | 1.0023 | 0.0752 | 4.67E-01 |
| Hmg1l1 | D3ZL49 | 2 | 8.17E-06 | 9.81E-06 | 4.18E-11 | 1.57E-11 | 0.8246 | 0.6163 | 5.42E-01 |

| | | | | | | | | | |
|---|---|---|---|---|---|---|---|---|---|
| Hnrnpa2b1 | A7VJC2 | 2 | 3.42E-05 | 3.35E-05 | 7.69E-11 | 7.06E-11 | 0.9570 | 0.1365 | 6.90E-01 |
| Hnrnph1 | G3V9Q3 | 2 | 8.06E-06 | 9.45E-06 | 4.83E-12 | 1.01E-11 | 0.8588 | 0.1251 | 8.70E-01 |
| Hnrpk | Q5D059 | 3 | 1.51E-05 | 1.49E-05 | 9.07E-12 | 1.10E-11 | 0.9956 | 0.0877 | 9.99E-01 |
| Hp | P06866 | 3 | 1.24E-05 | 2.47E-05 | 9.47E-11 | 5.43E-11 | 0.5042 | 0.1789 | 2.43E-05 |
| Hprt1 | P27605 | 5 | 5.80E-05 | 5.93E-05 | 1.37E-10 | 1.38E-10 | 0.9493 | 0.3823 | 5.31E-01 |
| Hpx | P20059 | 5 | 6.42E-05 | 8.14E-05 | 4.02E-10 | 6.73E-10 | 0.8176 | 0.2171 | 1.64E-02 |
| Hrc | Q80W59 | 2 | 9.90E-06 | 1.02E-05 | 6.11E-12 | 3.31E-12 | 0.9850 | 0.0815 | 9.63E-01 |
| Hsd17b10 | B0BMW2 | 5 | 1.43E-04 | 1.22E-04 | 1.01E-09 | 7.79E-10 | 1.1527 | 0.1941 | 3.68E-02 |
| Hsdl2 | Q4V8F9 | 4 | 6.98E-05 | 6.35E-05 | 2.80E-10 | 1.39E-10 | 1.0813 | 0.1065 | 6.62E-02 |
| Hsp90aa1 | P82995 | 5 | 3.13E-05 | 3.16E-05 | 1.44E-10 | 8.16E-11 | 1.0100 | 0.1417 | 9.72E-01 |
| Hsp90ab1 | P34058 | 5 | 1.01E-04 | 1.14E-04 | 2.41E-09 | 6.63E-10 | 0.8904 | 0.2734 | 3.35E-01 |
| Hsp90ab1; Hsp90aa1 | P34058; P82995 | 5 | 1.01E-04 | 1.06E-04 | 2.50E-10 | 1.82E-10 | 0.9381 | 0.0451 | 8.21E-01 |
| Hspa1l; Hspa8 | P55063; P63018 | 2 | 1.09E-04 | 1.08E-04 | 9.71E-11 | 6.16E-11 | 1.0058 | 0.0134 | 9.48E-01 |
| Hspa1l; Hspa8; Hspa5 | P55063; P63018; P06761 | 3 | 2.01E-04 | 1.94E-04 | 2.00E-10 | 3.58E-10 | 1.0359 | 0.0141 | 8.78E-01 |
| Hspa4 | F1LRV4 | 2 | 1.25E-05 | 1.36E-05 | 9.23E-12 | 5.03E-12 | 0.9110 | 0.0693 | 1.07E-01 |
| Hspa5 | P06761 | 5 | 3.97E-05 | 3.85E-05 | 8.22E-11 | 5.65E-11 | 1.0017 | 0.0807 | 7.70E-01 |
| Hspa8 | P63018 | 5 | 1.77E-04 | 1.78E-04 | 2.91E-10 | 1.88E-10 | 0.9823 | 0.0164 | 7.25E-01 |
| Hspa8; D3ZH98 | P63018; D3ZH98 | 3 | 7.12E-05 | 6.48E-05 | 1.30E-10 | 5.31E-11 | 1.2285 | 0.1994 | 5.97E-01 |
| Hspa9 | F1M953 | 5 | 1.40E-04 | 1.21E-04 | 1.36E-10 | 1.48E-10 | 1.1650 | 0.0307 | 7.29E-07 |
| Hspb1 | G3V913 | 5 | 1.24E-04 | 1.21E-04 | 4.03E-09 | 1.87E-09 | 0.9568 | 0.1758 | 4.58E-01 |
| Hspb2 | O35878 | 2 | 1.67E-05 | 1.83E-05 | 1.26E-11 | 5.83E-12 | 0.9106 | 0.0523 | 1.70E-01 |
| Hspb6 | P97541 | 5 | 2.23E-04 | 2.04E-04 | 1.39E-09 | 1.01E-09 | 1.0557 | 0.0826 | 7.64E-01 |
| Hspd1 | P63039 | 5 | 3.43E-04 | 3.09E-04 | 3.84E-09 | 2.17E-09 | 1.1209 | 0.0615 | 3.88E-02 |
| Hspe1 | P26772 | 5 | 2.35E-04 | 1.89E-04 | 5.67E-09 | 9.26E-10 | 1.1735 | 0.1973 | 7.04E-02 |
| Hspg2 | F1LTJ5 | 5 | 1.75E-05 | 1.57E-05 | 6.01E-11 | 1.80E-11 | 1.1974 | 0.3851 | 9.79E-02 |
| Hspg2 | F1M566 | 5 | 3.33E-05 | 3.07E-05 | 1.61E-10 | 5.64E-11 | 1.0673 | 0.2185 | 8.02E-01 |
| Hspg2; Hspg2 | F1LTJ5; F1M566 | 5 | 3.28E-05 | 2.80E-05 | 9.15E-11 | 5.43E-11 | 1.1710 | 0.2359 | 4.19E-01 |
| Idh1 | P41562 | 3 | 6.15E-05 | 4.42E-05 | 8.79E-10 | 7.33E-11 | 1.1912 | 0.4258 | 3.95E-01 |
| Idh2 | P56574 | 5 | 1.62E-03 | 1.59E-03 | 4.82E-08 | 4.31E-08 | 1.0313 | 0.0602 | 2.31E-01 |
| Idh2; Idh1 | P56574; P41562 | 2 | 1.08E-03 | 1.05E-03 | 7.98E-08 | 2.08E-08 | 1.0218 | 0.0770 | 5.99E-01 |
| Idh3a | F1LNF7 | 5 | 1.38E-04 | 1.33E-04 | 6.94E-10 | 7.43E-10 | 1.0459 | 0.0747 | 2.59E-01 |
| Idh3B | Q68FX0 | 5 | 8.48E-05 | 8.04E-05 | 3.48E-10 | 8.17E-11 | 0.9885 | 0.0764 | 9.09E-01 |
| Idh3g | P41565 | 5 | 5.56E-05 | 5.26E-05 | 1.43E-10 | 7.70E-11 | 1.0240 | 0.3323 | 6.86E-01 |
| Immt | Q3KR86 | 5 | 1.09E-04 | 9.68E-05 | 2.45E-10 | 1.12E-10 | 1.1347 | 0.0672 | 3.36E-04 |
| Itih3 | Q63416 | 2 | 1.29E-05 | 1.23E-05 | 2.81E-11 | 2.25E-11 | 1.0535 | 0.3267 | 5.38E-01 |
| Itih4 | Q5EBC0 | 5 | 1.20E-04 | 1.33E-04 | 2.99E-09 | 2.76E-09 | 0.8211 | 0.1869 | 5.36E-01 |
| Ivd | P12007 | 5 | 9.66E-05 | 8.42E-05 | 4.61E-10 | 9.14E-10 | 1.0902 | 0.1527 | 1.66E-01 |
| Jup | P70565 | 5 | 2.69E-05 | 2.28E-05 | 4.02E-11 | 1.92E-11 | 1.1136 | 0.1206 | 5.71E-01 |
| Krt1 | Q6IMF3 | 3 | 4.56E-05 | 3.50E-05 | 1.32E-08 | 5.49E-10 | 1.3047 | 13.8086 | 6.01E-03 |
| Krt10 | Q6IFW6 | 2 | 1.35E-05 | 9.21E-06 | 1.40E-09 | 1.39E-10 | 1.3873 | 17.1921 | 3.11E-02 |
| Krt6a | Q4FZU2 | 2 | 5.63E-05 | 3.16E-05 | 1.94E-08 | 1.27E-09 | 1.7790 | 23.4569 | 7.46E-04 |
| Krt8 | Q10758 | 3 | 2.08E-05 | 1.02E-05 | 5.95E-09 | 5.76E-10 | 2.1232 | 37.8976 | 9.21E-04 |

| | | | | | | | | | |
|---|---|---|---|---|---|---|---|---|---|
| Lama2 | F1M614 | 5 | 2.35E-05 | 2.20E-05 | 1.48E-11 | 2.16E-11 | 1.1126 | 0.1045 | 7.08E-03 |
| Lamb1 | D3ZQN7 | 5 | 2.66E-05 | 2.21E-05 | 2.72E-11 | 2.11E-11 | 1.1056 | 0.1238 | 1.26E-01 |
| Lamb2 | M0R6K0 | 5 | 3.75E-05 | 3.68E-05 | 1.06E-10 | 2.68E-10 | 1.1589 | 0.1610 | 3.47E-01 |
| Lamc1 | F1MAA7 | 5 | 3.92E-05 | 3.57E-05 | 6.28E-11 | 5.92E-11 | 1.1086 | 0.0915 | 1.20E-01 |
| Ldb3 | A0A096MJ01 | 5 | 9.51E-05 | 8.43E-05 | 5.15E-10 | 2.84E-10 | 1.0993 | 0.1933 | 7.31E-01 |
| Ldb3 | Q5XIG1 | 3 | 8.41E-05 | 8.59E-05 | 8.66E-10 | 6.49E-10 | 0.9715 | 0.1249 | 7.78E-01 |
| Ldb3; Ldb3 | Q5XIG1; A0A096MJ01 | 5 | 2.10E-04 | 2.16E-04 | 2.52E-09 | 1.44E-09 | 1.0071 | 0.0947 | 7.91E-01 |
| Ldha | P04642 | 5 | 3.75E-04 | 4.11E-04 | 3.67E-08 | 2.68E-08 | 0.8939 | 0.2557 | 5.67E-02 |
| Ldha; Ldhb | P04642; P42123 | 3 | 1.02E-03 | 1.05E-03 | 1.75E-07 | 1.25E-07 | 0.9857 | 0.1450 | 9.80E-01 |
| Ldhb | P42123 | 5 | 1.86E-03 | 1.95E-03 | 7.35E-07 | 5.14E-07 | 0.9628 | 0.2574 | 4.64E-01 |
| Letm1 | Q5XIN6 | 2 | 1.61E-05 | 1.67E-05 | 6.97E-12 | 1.58E-11 | 0.9662 | 0.0633 | 5.41E-01 |
| Lgals1 | P11762 | 5 | 5.80E-05 | 5.55E-05 | 1.03E-10 | 1.41E-10 | 1.0186 | 0.0729 | 6.69E-01 |
| Lmna | G3V8L3 | 5 | 5.98E-05 | 5.69E-05 | 1.10E-10 | 1.09E-10 | 0.9922 | 0.1341 | 4.61E-01 |
| LOC100360413 | M0R757 | 2 | 1.33E-05 | 1.64E-05 | 4.79E-11 | 6.96E-11 | 0.8117 | 0.3350 | 1.36E-01 |
| LOC100360413; Eef1a2 | M0R757; P62632 | 5 | 2.72E-04 | 2.75E-04 | 6.90E-10 | 5.87E-10 | 0.9484 | 0.0357 | 7.12E-01 |
| LOC100360791 | M0R8Q2 | 2 | 2.74E-05 | 3.25E-05 | 6.86E-11 | 1.81E-11 | 0.8573 | 0.0703 | 1.66E-02 |
| LOC100361144 | D4A4P3 | 3 | 2.22E-05 | 1.54E-05 | 1.77E-10 | 1.27E-10 | 1.4504 | 1.8801 | 3.13E-01 |
| LOC100362298 | D3ZM33 | 2 | 2.02E-05 | 1.83E-05 | 2.57E-11 | 8.81E-12 | 1.1456 | 0.1584 | 6.50E-02 |
| LOC100362339 | D4A6G6 | 2 | 1.55E-05 | 1.48E-05 | 2.75E-12 | 5.93E-12 | 1.0526 | 0.0413 | 2.68E-01 |
| LOC100363268 | B5DEL8 | 5 | 1.20E-04 | 1.26E-04 | 7.00E-10 | 3.95E-10 | 0.9203 | 0.0826 | 2.29E-01 |
| LOC100912386 | M0RAK8 | 2 | 1.38E-05 | 1.36E-05 | 2.86E-12 | 3.50E-12 | 1.0156 | 0.0349 | 9.06E-01 |
| LOC100912599 | D3ZCZ9 | 4 | 4.61E-05 | 4.48E-05 | 4.46E-10 | 5.94E-10 | 0.9748 | 0.9268 | 5.68E-01 |
| LOC102554591 | M0RCE8 | 2 | 1.51E-05 | 1.59E-05 | 1.58E-11 | 7.91E-12 | 0.9330 | 0.1105 | 5.39E-01 |
| LOC102554591 | F1LVX3 | 2 | 1.24E-05 | 1.33E-05 | 2.85E-11 | 2.70E-11 | 0.9644 | 0.2453 | 8.40E-01 |
| LOC287167 | Q63910 | 4 | 2.90E-05 | 2.15E-05 | 3.51E-10 | 3.54E-10 | 1.1398 | 1.0162 | 7.15E-01 |
| LOC367586 | Q5M7V3 | 5 | 2.18E-04 | 2.66E-04 | 5.03E-09 | 2.07E-08 | 0.7933 | 0.2381 | 2.37E-02 |
| LOC498555 | D4A4D5 | 2 | 2.73E-05 | 2.97E-05 | 2.45E-11 | 2.09E-11 | 0.8978 | 0.0583 | 5.42E-01 |
| LOC500183 | Q4KM66 | 5 | 1.61E-04 | 2.10E-04 | 2.75E-09 | 7.33E-09 | 0.7205 | 0.1720 | 1.64E-01 |
| LOC679794 | D4A5L9 | 5 | 6.99E-04 | 6.75E-04 | 5.50E-08 | 4.03E-08 | 0.9192 | 0.2003 | 5.36E-01 |

| | | | | | | | | | |
|---|---|---|---|---|---|---|---|---|---|
| LOC683884 | D3ZF13 | 4 | 2.39E-05 | 2.40E-05 | 3.04E-10 | 3.38E-10 | 0.7919 | 1.1827 | 5.04E-01 |
| LOC683961 | M0RCY2 | 2 | 9.73E-06 | 1.10E-05 | 2.91E-12 | 4.67E-12 | 0.9039 | 0.0618 | 7.58E-02 |
| LOC684828; Hist1h1e | M0R7B4; P15865 | 5 | 3.87E-04 | 3.58E-04 | 2.35E-09 | 4.85E-09 | 1.0283 | 0.0899 | 6.04E-01 |
| LOC688963 | F1LPG5 | 4 | 6.08E-05 | 6.02E-05 | 2.47E-10 | 1.82E-10 | 1.0327 | 0.2281 | 8.72E-01 |
| Lonp1 | Q924S5 | 2 | 8.39E-06 | 8.86E-06 | 3.08E-12 | 5.85E-12 | 0.8985 | 0.2460 | 6.69E-01 |
| Lrpprc | F1LM33 | 3 | 1.48E-05 | 1.32E-05 | 1.22E-11 | 2.60E-12 | 1.1029 | 0.1019 | 7.70E-01 |
| Lum | P51886 | 4 | 1.27E-04 | 1.22E-04 | 1.16E-09 | 1.04E-09 | 1.1514 | 1.1880 | 9.43E-01 |
| Lypla1 | P70470 | 2 | 2.15E-05 | 1.98E-05 | 1.11E-10 | 4.55E-11 | 1.0897 | 0.4753 | 5.60E-01 |
| Macrod1 | Q8K4G6 | 4 | 3.83E-05 | 3.60E-05 | 1.17E-10 | 7.64E-11 | 0.9959 | 0.1862 | 9.40E-01 |
| Maoa | G3V9Z3 | 5 | 4.12E-05 | 8.18E-05 | 6.61E-10 | 2.51E-09 | 0.5191 | 0.1898 | 1.51E-06 |
| Map1 | P01048 | 5 | 6.36E-05 | 4.78E-05 | 4.61E-10 | 3.99E-10 | 1.1355 | 0.4936 | 6.62E-01 |
| Mb | Q9QZ76 | 5 | 4.62E-03 | 5.29E-03 | 3.44E-06 | 4.71E-06 | 0.8649 | 0.1377 | 1.65E-01 |
| Mccc1 | F1LP30 | 5 | 2.59E-05 | 2.68E-05 | 3.87E-11 | 1.32E-11 | 1.0292 | 0.5651 | 5.65E-01 |
| Mccc2 | Q5XIT9 | 5 | 2.56E-05 | 2.43E-05 | 2.34E-11 | 2.04E-11 | 1.0170 | 0.0637 | 8.52E-01 |
| Mdh1 | O88989 | 5 | 6.45E-04 | 6.82E-04 | 3.26E-08 | 3.37E-08 | 0.9212 | 0.1271 | 3.21E-02 |
| Mdh2 | P04636 | 5 | 1.73E-03 | 1.81E-03 | 5.28E-08 | 5.01E-08 | 1.0351 | 0.0687 | 2.82E-01 |
| Me3 | F1M5N4 | 2 | 1.78E-05 | 1.63E-05 | 2.67E-11 | 8.92E-12 | 1.0961 | 0.1264 | 5.67E-02 |
| Mpc2 | P38718 | 3 | 9.92E-05 | 1.00E-04 | 4.36E-10 | 2.79E-10 | 0.8880 | 0.4527 | 5.18E-01 |
| Mpst | P97532 | 4 | 1.93E-05 | 2.22E-05 | 3.71E-11 | 1.96E-11 | 0.8924 | 0.2132 | 3.18E-01 |
| Msn | A0A096MK30 | 4 | 2.90E-05 | 2.98E-05 | 6.55E-11 | 3.22E-11 | 0.9380 | 0.0825 | 9.36E-01 |
| Mtch2 | B0BN52 | 4 | 5.18E-05 | 5.01E-05 | 5.58E-11 | 4.78E-11 | 1.0052 | 0.0389 | 9.96E-01 |
| Mtx2 | Q5U1Z9 | 2 | 2.14E-05 | 2.08E-05 | 2.20E-11 | 9.44E-12 | 1.0088 | 0.0721 | 7.09E-01 |
| Mug1 | Q03626 | 5 | 6.76E-05 | 6.95E-05 | 1.29E-09 | 4.14E-10 | 0.7824 | 0.1971 | 8.73E-03 |
| Mug1; A1i3 | Q03626; P14046 | 5 | 8.11E-05 | 1.39E-04 | 1.68E-09 | 2.80E-09 | 0.6320 | 0.0947 | 3.56E-04 |
| Murc | B1PRL5 | 4 | 1.97E-05 | 1.82E-05 | 9.20E-12 | 5.34E-12 | 1.1805 | 0.1773 | 2.68E-02 |
| Mut | D3ZKG1 | 3 | 2.69E-05 | 2.55E-05 | 1.69E-11 | 2.32E-11 | 1.0577 | 0.0660 | 1.56E-01 |
| Mybpc3 | P56741 | 5 | 4.37E-04 | 4.40E-04 | 9.28E-09 | 9.33E-09 | 0.9754 | 0.0855 | 9.50E-01 |
| Myh11 | E9PTU4 | 5 | 2.86E-05 | 1.59E-05 | 4.45E-10 | 6.53E-11 | 1.4331 | 7.1111 | 4.62E-01 |
| Myh6 | G3V885 | 5 | 3.54E-03 | 3.67E-03 | 8.03E-07 | 6.84E-07 | 0.9518 | 0.1131 | 4.22E-01 |
| Myh7 | G3V8B0 | 5 | 7.76E-04 | 7.98E-04 | 4.01E-07 | 3.76E-07 | 1.3256 | 1.6238 | 6.69E-01 |
| Myh7; Myh13; Myh6 | G3V8B0; F1M789; G3V885 | 5 | 3.58E-03 | 3.49E-03 | 4.05E-07 | 2.25E-07 | 1.0453 | 0.0891 | 8.33E-01 |
| Myh7; Myh13; Myh6; Myh7b | G3V8B0; F1M789; G3V885; B6RK61 | 5 | 2.26E-03 | 2.11E-03 | 2.08E-07 | 1.39E-07 | 1.0461 | 0.1660 | 8.31E-01 |
| Myh7; Myh6 | G3V8B0; G3V885 | 5 | 4.56E-03 | 4.46E-03 | 6.56E-07 | 5.38E-07 | 1.0288 | 0.0743 | 6.07E-01 |
| Myh7; Myh6; Myh7b | G3V8B0; G3V885; B6RK61 | 5 | 1.85E-03 | 1.91E-03 | 2.92E-07 | 2.27E-07 | 3.3258 | 24.1659 | 5.07E-01 |
| Myh9; Myh11 | Q62812; E9PTU4 | 4 | 1.43E-05 | 1.35E-05 | 2.86E-10 | 3.43E-11 | 1.1683 | 8.7955 | 1.30E-01 |

| | | | | | | | | | |
|---|---|---|---|---|---|---|---|---|---|
| Myl2 | P08733 | 5 | 2.55E-03 | 2.61E-03 | 3.40E-07 | 3.80E-07 | 0.9729 | 0.2152 | 9.42E-01 |
| Myl3 | P16409 | 5 | 3.57E-03 | 3.26E-03 | 6.59E-07 | 5.79E-07 | 1.0386 | 0.1233 | 7.70E-01 |
| Myl4 | P17209 | 4 | 4.42E-06 | 7.90E-05 | 1.86E-10 | 2.62E-08 | 0.0711 | 0.0573 | 5.98E-04 |
| Myl7 | F1M7K3 | 5 | 2.15E-05 | 5.70E-05 | 7.29E-11 | 1.18E-08 | 0.3778 | 34.7821 | 1.75E-01 |
| Myom1 | F1M7T8 | 5 | 9.64E-05 | 1.01E-04 | 4.72E-10 | 5.45E-10 | 1.0202 | 0.0991 | 3.39E-01 |
| Myom1; Myom1 | F1M7T8; Q80UL1 | 2 | 2.37E-05 | 2.68E-05 | 2.41E-11 | 1.65E-11 | 0.8882 | 0.0508 | 9.93E-02 |
| Myom2 | G3V7K1 | 5 | 7.62E-04 | 8.23E-04 | 3.15E-08 | 1.09E-08 | 0.9624 | 0.0458 | 8.00E-01 |
| Myoz2 | D3ZX18 | 5 | 1.59E-04 | 1.57E-04 | 1.45E-09 | 8.42E-10 | 1.0422 | 0.0988 | 6.89E-01 |
| ND5 | A0A096XKT9 | 2 | 4.60E-05 | 4.23E-05 | 1.52E-10 | 1.52E-10 | 1.2310 | 0.4572 | 1.87E-01 |
| Ndrg2 | Q8VBU2 | 4 | 7.62E-05 | 6.91E-05 | 8.57E-11 | 9.37E-11 | 1.0396 | 0.0771 | 9.37E-01 |
| Ndufa10 | Q561S0 | 5 | 1.55E-04 | 1.62E-04 | 2.28E-09 | 1.34E-09 | 0.9968 | 0.0945 | 5.05E-01 |
| Ndufa11 | Q80W89 | 3 | 4.80E-05 | 5.02E-05 | 7.34E-11 | 5.31E-11 | 0.9461 | 0.0511 | 5.21E-01 |
| Ndufa12 | F1LXA0 | 4 | 1.75E-04 | 1.45E-04 | 8.04E-09 | 4.81E-10 | 1.1532 | 0.2766 | 2.93E-01 |
| Ndufa13 | F1LZC5 | 3 | 1.25E-04 | 1.18E-04 | 4.16E-10 | 3.23E-10 | 0.9880 | 0.2281 | 5.84E-01 |
| Ndufa2 | D3ZS58 | 5 | 1.71E-04 | 1.52E-04 | 5.05E-10 | 4.39E-10 | 1.1158 | 0.0326 | 3.78E-01 |
| Ndufa4 | B2RZD6 | 5 | 6.31E-04 | 5.97E-04 | 8.79E-09 | 4.98E-09 | 1.0825 | 0.0403 | 6.37E-01 |
| Ndufa5 | Q63362 | 5 | 9.93E-05 | 1.02E-04 | 2.92E-10 | 2.12E-10 | 0.9870 | 0.0455 | 9.55E-01 |
| Ndufa6 | D4A3V2 | 5 | 1.14E-04 | 1.23E-04 | 1.72E-09 | 9.03E-10 | 0.8450 | 0.2069 | 9.46E-01 |
| Ndufa7l | A9UMV9 | 4 | 9.24E-05 | 8.59E-05 | 1.81E-10 | 1.69E-10 | 1.1537 | 0.2272 | 5.22E-01 |
| Ndufa8 | Q7TP78 | 3 | 1.29E-04 | 1.28E-04 | 6.03E-10 | 7.94E-10 | 1.0055 | 0.0607 | 6.46E-01 |
| Ndufa9 | Q5BK63 | 5 | 1.73E-04 | 1.71E-04 | 3.28E-09 | 1.62E-09 | 1.0595 | 0.1308 | 8.63E-02 |
| Ndufb10 | D4A0T0 | 5 | 2.09E-04 | 1.70E-04 | 2.06E-08 | 1.81E-09 | 1.1247 | 0.3815 | 2.03E-01 |
| Ndufb11 | D4A7L4 | 2 | 1.80E-04 | 1.59E-04 | 9.37E-10 | 7.50E-10 | 1.1056 | 0.0727 | 6.42E-01 |
| Ndufb5 | D4A565 | 4 | 9.64E-05 | 1.02E-04 | 5.28E-10 | 3.25E-10 | 0.9462 | 0.1131 | 8.69E-01 |
| Ndufb6 | D3ZZ21 | 4 | 6.94E-05 | 6.22E-05 | 4.29E-10 | 2.81E-10 | 1.4534 | 7.7927 | 7.36E-01 |
| Ndufb7 | D3ZLT1 | 4 | 8.38E-05 | 8.46E-05 | 2.91E-10 | 2.00E-10 | 0.8506 | 0.1320 | 5.22E-01 |
| Ndufb8 | B2RYS8 | 3 | 5.94E-05 | 7.31E-05 | 5.85E-10 | 2.07E-10 | 0.8751 | 0.2121 | 6.29E-01 |
| Ndufb9 | B2RYW3 | 4 | 8.73E-05 | 8.78E-05 | 3.52E-10 | 2.92E-10 | 0.8695 | 0.0832 | 3.95E-01 |
| Ndufc2 | Q5PQZ9 | 2 | 9.73E-05 | 9.94E-05 | 4.00E-10 | 6.08E-10 | 0.9888 | 0.1004 | 8.45E-01 |
| Ndufs1 | Q66HF1 | 5 | 2.54E-04 | 2.31E-04 | 9.40E-10 | 9.19E-10 | 1.0541 | 0.0533 | 6.79E-02 |
| Ndufs2 | Q641Y2 | 5 | 1.74E-04 | 1.68E-04 | 4.34E-10 | 6.86E-10 | 0.9671 | 0.0492 | 7.09E-01 |
| Ndufs3 | D3ZG43 | 5 | 1.85E-04 | 1.79E-04 | 6.16E-10 | 4.36E-10 | 1.0121 | 0.0396 | 6.85E-01 |
| Ndufs4 | Q5XIF3 | 5 | 1.16E-04 | 1.21E-04 | 6.64E-10 | 6.06E-10 | 0.9663 | 0.1093 | 8.68E-01 |
| Ndufs7 | Q5RJN0 | 5 | 1.10E-04 | 1.04E-04 | 2.37E-10 | 2.31E-10 | 1.0880 | 12.2029 | 6.45E-01 |
| Ndufs8 | B0BNE6 | 4 | 6.40E-05 | 6.48E-05 | 3.53E-10 | 3.32E-10 | 0.8098 | 0.1323 | 5.64E-01 |
| Ndufv1 | Q5XIH3 | 5 | 1.95E-04 | 1.70E-04 | 4.94E-10 | 3.27E-10 | 1.1310 | 0.0480 | 1.60E-02 |
| Ndufv2 | P19234 | 5 | 2.58E-04 | 2.38E-04 | 1.42E-09 | 1.44E-09 | 1.0554 | 0.0482 | 6.56E-01 |
| Ndufv3 | Q6PCU8 | 2 | 5.38E-05 | 4.30E-05 | 2.73E-10 | 2.28E-10 | 1.2515 | 0.3416 | 4.68E-01 |
| Nebl | D4A164 | 5 | 2.75E-05 | 2.78E-05 | 2.46E-11 | 2.35E-11 | 0.9137 | 0.1696 | 7.81E-01 |
| Nid1 | F1LM84 | 5 | 2.97E-05 | 2.68E-05 | 4.51E-11 | 3.91E-11 | 1.1226 | 0.2421 | 2.57E-01 |
| Nit2 | Q497B0 | 5 | 4.29E-05 | 4.00E-05 | 1.62E-10 | 2.22E-10 | 1.0945 | 0.4263 | 7.48E-01 |
| Nme2 | P19804 | 5 | 3.52E-04 | 3.42E-04 | 9.75E-09 | 7.53E-09 | 0.9886 | 0.1340 | 6.18E-01 |
| Nnt | Q5BJZ3 | 5 | 3.15E-04 | 2.96E-04 | 4.59E-08 | 2.38E-08 | 1.0287 | 0.7646 | 4.35E-01 |
| Nppa | P01161 | 4 | 1.33E-05 | 3.54E-05 | 2.16E-11 | 4.31E-08 | 0.3614 | 18.9675 | 2.87E-04 |
| Nqo2 | Q6AY80 | 2 | 1.90E-05 | 1.55E-05 | 3.18E-10 | 3.66E-11 | 1.2226 | 1.5644 | 9.29E-02 |
| Ogdh | Q5XI78 | 5 | 2.15E-04 | 1.99E-04 | 5.31E-10 | 5.77E-10 | 1.0516 | 0.0299 | 3.29E-01 |
| Ogn | D3ZVB7 | 5 | 2.48E-05 | 2.74E-05 | 5.28E-10 | 3.53E-10 | 0.9555 | 1.5066 | 7.35E-01 |
| Ola1 | A0JPJ7 | 2 | 1.51E-05 | 1.61E-05 | 1.90E-11 | 9.04E-12 | 0.9425 | 0.1357 | 7.38E-01 |
| Opa1 | D4A8U5 | 3 | 9.69E-06 | 9.21E-06 | 4.36E-12 | 5.78E-12 | 1.0410 | 0.1243 | 9.72E-01 |

| | | | | | | | | | |
|---|---|---|---|---|---|---|---|---|---|
| Oxct1 | B2GV06 | 5 | 2.31E-04 | 2.17E-04 | 4.64E-09 | 2.17E-09 | 1.0627 | 0.1455 | 7.71E-01 |
| Park7 | O88767 | 5 | 1.82E-04 | 1.75E-04 | 1.04E-08 | 4.93E-09 | 1.0209 | 0.2839 | 9.51E-01 |
| Pcca | P14882 | 2 | 5.29E-05 | 4.23E-05 | 1.77E-10 | 8.78E-11 | 1.1947 | 0.1069 | 1.17E-01 |
| Pcca; Pcca | D4A882; P14882 | 5 | 5.44E-05 | 5.65E-05 | 4.10E-11 | 5.00E-11 | 0.9450 | 0.0355 | 9.76E-01 |
| Pccb | Q68FZ8 | 2 | 4.43E-05 | 4.44E-05 | 4.95E-11 | 4.24E-11 | 1.0040 | 0.0452 | 8.08E-01 |
| Pccb; Pccb | P07633; Q68FZ8 | 5 | 5.04E-05 | 4.72E-05 | 5.65E-11 | 4.57E-11 | 1.0223 | 0.0500 | 3.86E-01 |
| Pdha1l1 | D4A5G8 | 5 | 3.81E-04 | 3.53E-04 | 1.29E-09 | 2.05E-09 | 1.0443 | 0.0431 | 6.48E-01 |
| Pdhb | P49432 | 5 | 3.75E-04 | 3.64E-04 | 2.37E-09 | 1.77E-09 | 1.0490 | 0.0347 | 5.20E-01 |
| Pdhx | Q7TQ85 | 4 | 5.81E-05 | 5.17E-05 | 5.13E-11 | 6.69E-11 | 1.1181 | 0.0601 | 5.09E-01 |
| Pdia3 | P11598 | 4 | 1.39E-04 | 9.62E-05 | 1.92E-09 | 8.07E-10 | 1.2097 | 0.2522 | 3.76E-01 |
| Pdk1 | F1MA54 | 4 | 3.44E-05 | 2.80E-05 | 4.85E-11 | 2.47E-11 | 1.1943 | 0.1219 | 2.20E-01 |
| Pdk2 | Q64536 | 2 | 2.89E-05 | 2.69E-05 | 1.10E-11 | 9.45E-12 | 1.0787 | 0.0302 | 7.42E-02 |
| Pdlim5 | Q62920 | 5 | 9.29E-05 | 9.72E-05 | 7.29E-10 | 4.15E-10 | 0.8925 | 0.1893 | 7.48E-01 |
| Pebp1 | P31044 | 5 | 1.60E-04 | 1.63E-04 | 3.53E-09 | 2.57E-09 | 0.9338 | 0.1864 | 4.04E-01 |
| Pfkm | Q52KS1 | 5 | 8.04E-05 | 7.63E-05 | 3.67E-10 | 7.27E-11 | 0.9985 | 0.1532 | 7.20E-01 |
| Pfn1 | P62963 | 3 | 5.86E-05 | 6.34E-05 | 4.83E-10 | 2.01E-10 | 1.2066 | 0.1620 | 4.57E-01 |
| Pgam1 | P25113 | 5 | 4.54E-05 | 5.14E-05 | 9.81E-11 | 4.40E-10 | 0.8193 | 0.1699 | 1.83E-01 |
| Pgam2 | P16290 | 5 | 2.44E-04 | 2.30E-04 | 4.22E-09 | 7.16E-09 | 0.9822 | 0.4810 | 4.46E-01 |
| Pgam2; Pgam1 | P16290; P25113 | 3 | 9.85E-05 | 1.13E-04 | 6.83E-10 | 4.29E-10 | 0.9178 | 0.1796 | 7.35E-01 |
| Pgk1 | P16617 | 5 | 3.22E-04 | 3.31E-04 | 5.85E-09 | 4.44E-09 | 0.9684 | 0.0904 | 5.55E-01 |
| Pgm1 | A1A5L2 | 5 | 4.50E-05 | 5.26E-05 | 1.65E-10 | 1.10E-10 | 0.9009 | 0.1023 | 2.00E-01 |
| Phb | P67779 | 5 | 9.02E-05 | 8.20E-05 | 1.70E-10 | 1.43E-10 | 1.1122 | 0.0641 | 1.08E-01 |
| Phb2 | Q5XIH7 | 5 | 8.37E-05 | 8.15E-05 | 3.33E-10 | 2.60E-10 | 1.1000 | 0.1757 | 2.24E-01 |
| Pkm | P11980 | 5 | 2.53E-04 | 2.63E-04 | 6.09E-09 | 4.75E-09 | 0.7861 | 0.1733 | 4.29E-01 |
| Plectin 6 | Q6S3A0 | 5 | 1.98E-05 | 2.50E-05 | 1.86E-10 | 4.32E-11 | 0.9510 | 0.2968 | 8.27E-01 |
| Plin4 | M0R7S5 | 2 | 6.95E-05 | 6.84E-05 | 7.60E-11 | 4.94E-11 | 1.0172 | 0.0284 | 7.14E-01 |
| Ppia | P10111 | 2 | 9.44E-05 | 1.11E-04 | 4.07E-10 | 5.41E-10 | 0.8575 | 0.0642 | 3.07E-01 |
| Ppp2r1a | Q5XI34 | 3 | 2.62E-05 | 2.59E-05 | 2.28E-11 | 2.00E-11 | 0.8999 | 0.1047 | 5.46E-02 |
| Prdx1 | Q63716 | 5 | 7.04E-05 | 7.21E-05 | 3.32E-10 | 2.50E-10 | 0.9372 | 0.1025 | 2.83E-01 |
| Prdx2 | P35704 | 5 | 1.51E-04 | 1.71E-04 | 2.52E-09 | 4.77E-09 | 0.9018 | 0.1575 | 4.15E-01 |
| Prdx3 | G3V7I0 | 5 | 1.53E-04 | 1.55E-04 | 4.01E-10 | 4.70E-10 | 0.9547 | 0.1755 | 6.89E-01 |
| Prdx5 | Q9R063 | 5 | 1.45E-04 | 1.32E-04 | 1.57E-09 | 8.95E-10 | 1.0688 | 0.2172 | 7.04E-01 |
| Prdx6 | O35244 | 5 | 1.28E-04 | 1.28E-04 | 1.15E-09 | 1.00E-09 | 0.9875 | 0.2491 | 6.01E-01 |
| Prelp | Q9EQP5 | 4 | 3.38E-05 | 3.34E-05 | 3.27E-10 | 3.63E-10 | 1.0157 | 5.9261 | 7.34E-01 |
| Prkar1a | P09456 | 3 | 1.68E-05 | 1.68E-05 | 4.06E-11 | 1.21E-11 | 0.9175 | 0.1036 | 6.20E-01 |
| Psap | F7EPE0 | 2 | 3.26E-05 | 3.74E-05 | 3.38E-11 | 6.73E-11 | 0.8682 | 0.0779 | 1.85E-01 |
| Psme1 | Q6P9V7 | 3 | 2.47E-05 | 2.74E-05 | 3.04E-10 | 7.69E-11 | 0.8916 | 0.4198 | 6.80E-01 |
| Psme3 | Q7TP38 | 2 | 2.52E-05 | 1.05E-05 | 1.82E-10 | 4.63E-12 | 2.2469 | 1.8244 | 2.78E-01 |
| Ptgr2 | Q5BK81 | 2 | 1.77E-05 | 1.64E-05 | 1.07E-11 | 2.42E-11 | 1.0736 | 0.1442 | 5.15E-01 |
| Ptrf | G3V8L9 | 3 | 8.14E-05 | 7.90E-05 | 1.01E-10 | 2.01E-10 | 1.0328 | 0.0512 | 4.89E-01 |
| Pygb | B2GV03 | 5 | 5.09E-05 | 5.15E-05 | 1.15E-10 | 3.78E-11 | 1.2468 | 0.3110 | 3.91E-03 |
| Pygb; Pygm | B2GV03; B1WBU9 | 5 | 1.81E-04 | 1.84E-04 | 2.52E-09 | 8.33E-10 | 0.9543 | 0.0633 | 8.94E-01 |
| Pygm | B1WBU9 | 5 | 1.84E-04 | 1.69E-04 | 6.45E-10 | 3.58E-10 | 1.0171 | 0.0387 | 5.93E-01 |
| Ran | P62828 | 2 | 4.37E-05 | 5.10E-05 | 2.56E-10 | 1.03E-10 | 0.8731 | 0.0874 | 8.26E-01 |
| Rat apolipopr | Q65ZS7 | 4 | 5.48E-05 | 3.53E-05 | 8.85E-10 | 2.61E-10 | 1.5127 | 1.5797 | 1.57E-02 |

| | | | | | | | | | |
|---|---|---|---|---|---|---|---|---|---|
| otein E protein | | | | | | | | | |
| Reep5 | B2RZ37 | 3 | 3.01E-05 | 2.66E-05 | 3.34E-11 | 1.64E-11 | 1.0614 | 0.1239 | 9.88E-01 |
| RGD1564 515 | F1LYQ4 | 2 | 8.34E-06 | 1.82E-05 | 2.47E-11 | 8.93E-11 | 0.4478 | 0.1146 | 8.81E-03 |
| Rhoc | B2RYP0 | 2 | 1.62E-05 | 1.67E-05 | 1.85E-11 | 5.71E-12 | 0.9077 | 0.0689 | 5.30E-01 |
| Rmdn1 | Q4G069 | 3 | 3.60E-05 | 2.66E-05 | 1.58E-10 | 2.68E-11 | 1.2306 | 0.1965 | 2.03E-01 |
| Rpl18 | P12001 | 2 | 1.53E-05 | 1.52E-05 | 4.27E-12 | 8.23E-12 | 1.0043 | 0.0536 | 9.64E-01 |
| Rpl4 | Q6P3V9 | 2 | 9.52E-06 | 1.04E-05 | 3.59E-12 | 5.24E-12 | 0.9158 | 0.0822 | 2.49E-02 |
| Rplp0 | P19945 | 2 | 1.29E-05 | 1.39E-05 | 2.76E-12 | 5.18E-12 | 0.9255 | 0.0352 | 9.75E-01 |
| Rplp1 | P19944 | 2 | 1.90E-05 | 1.75E-05 | 1.92E-11 | 2.75E-11 | 1.0849 | 0.2100 | 5.52E-01 |
| Rps16 | Q6P3E1 | 2 | 2.34E-05 | 2.18E-05 | 3.28E-11 | 1.69E-11 | 1.0743 | 0.1136 | 6.50E-01 |
| Rps27a | P62982 | 2 | 4.07E-04 | 4.57E-04 | 6.31E-09 | 5.00E-09 | 0.8901 | 0.0393 | 3.03E-01 |
| Rps27a; LOC67959 4 | P62982; F1M0Q4 | 3 | 3.79E-04 | 3.67E-04 | 1.49E-08 | 3.27E-09 | 0.9571 | 0.0615 | 8.96E-01 |
| Rps7 | P62083 | 2 | 1.21E-05 | 1.15E-05 | 3.38E-11 | 4.81E-12 | 0.9961 | 0.2167 | 5.64E-01 |
| Rpsa | P38983 | 2 | 1.08E-05 | 1.28E-05 | 9.50E-12 | 7.08E-12 | 0.8166 | 0.0874 | 2.06E-01 |
| Ryr2 | B0LPN4 | 4 | 2.80E-05 | 2.46E-05 | 1.77E-11 | 1.36E-11 | 1.1054 | 0.0575 | 1.40E-02 |
| Samm50 | Q6AXV4 | 5 | 1.03E-04 | 7.89E-05 | 1.85E-09 | 3.24E-10 | 1.2494 | 0.2815 | 1.49E-02 |
| Scp2 | P11915 | 2 | 2.66E-05 | 1.93E-05 | 9.38E-11 | 1.22E-11 | 1.3861 | 0.3120 | 2.49E-06 |
| Sdha | Q920L2 | 5 | 2.47E-04 | 2.51E-04 | 2.06E-09 | 1.99E-09 | 0.9982 | 0.0550 | 7.83E-01 |
| Sdhb | P21913 | 5 | 1.76E-04 | 1.77E-04 | 6.75E-10 | 1.01E-09 | 1.0679 | 0.0747 | 3.39E-01 |
| Sdhc | Q641Z9 | 2 | 5.74E-05 | 6.01E-05 | 5.50E-11 | 1.04E-10 | 0.8930 | 0.0381 | 6.15E-01 |
| Sdpr | Q66H98 | 2 | 1.04E-05 | 1.11E-05 | 3.16E-12 | 2.49E-12 | 0.9368 | 0.0423 | 3.11E-01 |
| Selenbp1 | Q8VIF7 | 5 | 3.09E-05 | 3.35E-05 | 1.81E-10 | 8.29E-11 | 0.8897 | 0.1858 | 4.26E-01 |
| Serpina1 | P17475 | 5 | 1.46E-04 | 1.29E-04 | 4.41E-09 | 2.25E-09 | 1.1295 | 0.3932 | 3.05E-01 |
| Serpina3k | P05545 | 4 | 6.19E-05 | 4.71E-05 | 7.91E-10 | 2.96E-10 | 0.9760 | 0.5732 | 8.82E-01 |
| Serpina3l | P05544 | 3 | 3.52E-05 | 4.63E-05 | 3.37E-10 | 3.71E-10 | 0.7387 | 0.2264 | 4.26E-01 |
| Serpinc1 | Q5M7T5 | 3 | 4.72E-05 | 2.94E-05 | 2.08E-09 | 2.26E-10 | 1.1886 | 0.9699 | 8.58E-01 |
| Sgca | D3ZDQ9 | 3 | 3.89E-05 | 3.85E-05 | 9.89E-11 | 7.97E-11 | 1.0172 | 0.0843 | 9.81E-01 |
| Slc25a11 | G3V6H5 | 5 | 1.28E-04 | 1.17E-04 | 4.15E-10 | 3.18E-10 | 1.0501 | 0.0830 | 5.24E-01 |
| Slc25a12 | F1LX07 | 5 | 6.35E-05 | 6.00E-05 | 1.18E-10 | 5.43E-11 | 1.0665 | 0.0509 | 5.40E-01 |
| Slc25a13 | F1LZW6 | 3 | 3.17E-05 | 2.63E-05 | 7.70E-11 | 4.40E-11 | 1.2069 | 0.2276 | 3.36E-02 |
| Slc25a13; Slc25a12 | F1LZW6; F1LX07 | 4 | 8.03E-05 | 7.42E-05 | 1.27E-10 | 9.34E-11 | 1.0709 | 0.0862 | 1.55E-01 |
| Slc25a20 | P97521 | 5 | 6.71E-05 | 6.22E-05 | 7.97E-11 | 9.94E-11 | 1.0582 | 0.0539 | 1.69E-01 |
| Slc25a3 | Q6IRH6 | 5 | 6.19E-04 | 5.99E-04 | 6.11E-09 | 9.27E-09 | 0.9929 | 0.0707 | 7.10E-01 |
| Slc25a4 | Q05962 | 5 | 1.72E-03 | 1.68E-03 | 8.62E-08 | 3.95E-08 | 1.0276 | 0.2776 | 4.93E-01 |
| Slc25a5 | Q09073 | 4 | 8.68E-05 | 8.34E-05 | 7.03E-10 | 2.42E-10 | 1.0592 | 0.2824 | 8.26E-01 |
| Slc25a5; Slc25a31; Slc25a4 | Q09073; D3ZB81; Q05962 | 3 | 1.84E-03 | 1.71E-03 | 9.17E-08 | 4.95E-08 | 1.1079 | 2.0620 | 7.78E-01 |
| Slc25a5; Slc25a4 | Q09073; Q05962 | 5 | 2.36E-03 | 2.13E-03 | 1.22E-07 | 7.64E-08 | 1.1941 | 2.1076 | 7.63E-01 |
| Slc4a1 | F8WFT7 | 4 | 1.12E-05 | 1.11E-05 | 1.68E-11 | 7.84E-11 | 0.9746 | 0.7769 | 5.62E-01 |
| Smpx | Q925F0 | 2 | 1.45E-05 | 1.59E-05 | 1.52E-11 | 5.30E-11 | 0.9110 | 0.2357 | 9.66E-02 |
| Smyd1 | D4A3D2 | 4 | 3.43E-05 | 3.10E-05 | 1.22E-10 | 3.46E-11 | 1.0310 | 0.1295 | 9.90E-01 |
| Sod2 | P07895 | 5 | 4.38E-04 | 3.99E-04 | 3.59E-09 | 3.06E-09 | 1.0617 | 0.1402 | 7.64E-01 |
| Sorbs1; Sorbs1 | F1M820; F1M8Z8 | 3 | 1.58E-05 | 1.46E-05 | 1.28E-11 | 9.66E-12 | 1.0743 | 0.1219 | 9.35E-01 |

| | | | | | | | | | |
|---|---|---|---|---|---|---|---|---|---|
| Sorbs2 | F1LPM3 | 2 | 1.98E-05 | 2.07E-05 | 2.71E-11 | 2.25E-11 | 0.9595 | 0.1228 | 2.91E-01 |
| SP120 | Q63555 | 2 | 1.30E-05 | 1.10E-05 | 3.82E-10 | 2.44E-10 | 1.2398 | 20.6723 | 8.68E-02 |
| Sptbn1 | Q6XD99 | 5 | 2.27E-05 | 2.04E-05 | 3.43E-11 | 1.44E-11 | 1.0904 | 0.1045 | 9.18E-02 |
| Srl | F1LWG8 | 5 | 2.63E-04 | 2.48E-04 | 1.04E-09 | 2.07E-09 | 1.0200 | 0.0565 | 8.13E-01 |
| Sucla2 | F1LM47 | 5 | 2.58E-04 | 2.85E-04 | 1.17E-09 | 1.21E-09 | 0.9736 | 0.0369 | 8.58E-01 |
| Suclg1 | P13086 | 5 | 3.45E-04 | 2.77E-04 | 1.22E-08 | 3.25E-09 | 1.1420 | 0.1343 | 1.11E-01 |
| Suclg2 | B1H270 | 5 | 7.28E-05 | 6.13E-05 | 1.05E-10 | 5.90E-11 | 1.2122 | 0.0537 | 1.66E-06 |
| Superoxide dismutase [Cu-Zn] (Fragment) | Q6PEC5 | 5 | 2.90E-04 | 3.24E-04 | 1.02E-08 | 4.36E-09 | 0.9367 | 0.1264 | 6.51E-01 |
| Taf3 | Q6QI09 | 5 | 6.31E-04 | 5.27E-04 | 1.23E-08 | 6.13E-09 | 1.0688 | 0.0753 | 6.94E-01 |
| Tagln | P31232 | 5 | 1.45E-05 | 7.47E-06 | 2.32E-09 | 6.26E-10 | 1.9006 | 98.0218 | 1.02E-02 |
| Tf | P12346 | 5 | 2.66E-04 | 3.68E-04 | 1.91E-08 | 9.80E-09 | 0.8142 | 0.2407 | 1.15E-01 |
| Tgm2 | Q6P6R6 | 5 | 2.01E-05 | 2.14E-05 | 4.28E-11 | 1.47E-11 | 0.9197 | 0.1364 | 8.20E-02 |
| Timm21 | Q5U2X7 | 3 | 1.67E-05 | 1.52E-05 | 6.53E-12 | 5.06E-12 | 1.1111 | 0.0599 | 9.68E-02 |
| Titin (Fragment) | Q9JJ49 | 5 | 6.44E-05 | 6.31E-05 | 2.28E-10 | 1.26E-10 | 1.0034 | 0.1069 | 5.97E-01 |
| Titin (Fragment) | Q63580 | 5 | 9.23E-05 | 9.31E-05 | 5.89E-10 | 4.97E-10 | 1.0261 | 0.0982 | 7.91E-01 |
| Titin protein homolog (Fragment) | P97851 | 2 | 6.19E-05 | 6.53E-05 | 2.39E-10 | 9.70E-11 | 0.9514 | 0.0777 | 2.37E-01 |
| Tln1 | G3V852 | 2 | 7.12E-06 | 7.52E-06 | 7.13E-12 | 3.21E-12 | 0.9318 | 0.1597 | 5.42E-01 |
| Tmod1 | P70567 | 2 | 9.45E-06 | 1.02E-05 | 8.16E-12 | 6.72E-12 | 0.9174 | 0.1366 | 3.15E-01 |
| Tnnc1 | Q4PP99 | 5 | 4.12E-04 | 4.01E-04 | 1.80E-08 | 1.56E-08 | 1.0552 | 0.1718 | 9.61E-01 |
| Tnni3 | P23693 | 5 | 5.10E-04 | 5.00E-04 | 9.43E-09 | 9.38E-09 | 1.0085 | 0.2696 | 9.33E-01 |
| Tnnt2 | F1LQ95 | 5 | 7.91E-04 | 9.20E-04 | 2.84E-08 | 1.64E-08 | 0.8948 | 0.0835 | 1.59E-02 |
| Tpi1 | P48500 | 5 | 3.08E-04 | 3.14E-04 | 1.02E-08 | 8.86E-09 | 0.9496 | 0.1489 | 2.55E-01 |
| Tpm1 | F7FK40 | 2 | 4.11E-05 | 2.33E-05 | 1.63E-09 | 2.25E-10 | 1.8349 | 5.0166 | 3.22E-03 |
| Tpm1 | P04692 | 5 | 9.72E-04 | 1.05E-03 | 8.06E-08 | 4.29E-08 | 0.9896 | 0.1581 | 3.94E-01 |
| Tpm1; Tpm1 | F7FK40; P04692 | 5 | 1.30E-03 | 1.22E-03 | 5.29E-08 | 2.80E-08 | 1.0811 | 0.0594 | 4.71E-01 |
| Tpm2 | Q5FVG5 | 3 | 1.10E-05 | 9.91E-06 | 5.96E-10 | 1.08E-10 | 1.0997 | 9.8873 | 6.29E-01 |
| Tpm2; Tpm1 | Q5FVG5; P04692 | 2 | 1.11E-03 | 1.07E-03 | 6.93E-08 | 3.26E-08 | 1.0310 | 0.1910 | 9.39E-01 |
| Tpm2; Tpm1; Tpm1 | Q5FVG5; F7FK40; P04692 | 2 | 9.21E-04 | 8.32E-04 | 6.91E-08 | 4.08E-08 | 1.1481 | 0.1120 | 8.28E-01 |
| Tpm3; Tpm1; Tpm1 | Q63610; F7FK40; P04692 | 3 | 3.43E-04 | 3.30E-04 | 2.39E-08 | 2.27E-08 | 0.9613 | 0.4269 | 6.55E-01 |
| Tpm3; Tpm2; | Q63610; Q5FVG5; | 5 | 1.67E-03 | 1.56E-03 | 1.39E-07 | 8.24E-08 | 1.0404 | 0.0848 | 9.62E-01 |

| | | | | | | | | | |
|---|---|---|---|---|---|---|---|---|---|
| Tpm1; Tpm1 | F7FK40; P04692 | | | | | | | | |
| Trim72 | A0JPQ4 | 5 | 3.60E-05 | 3.41E-05 | 5.34E-11 | 5.29E-11 | 1.0439 | 0.0935 | 7.21E-01 |
| Tst | P24329 | 2 | 2.28E-05 | 2.46E-05 | 4.79E-11 | 4.95E-11 | 0.8568 | 0.1269 | 5.82E-01 |
| Tuba1a | P68370 | 5 | 4.50E-05 | 7.17E-05 | 2.85E-09 | 2.88E-09 | 0.7819 | 0.4687 | 4.56E-01 |
| Tuba1a; Tuba4a | P68370; Q5XIF6 | 5 | 1.26E-04 | 1.35E-04 | 5.17E-10 | 6.43E-10 | 0.9735 | 0.1207 | 8.12E-01 |
| Tuba4a | Q5XIF6 | 5 | 9.25E-05 | 9.49E-05 | 6.53E-10 | 4.68E-10 | 0.9731 | 0.1009 | 6.02E-01 |
| Tubb4b | G3V7C6 | 4 | 4.03E-05 | 4.39E-05 | 9.81E-11 | 7.76E-11 | 0.8814 | 0.3764 | 8.07E-01 |
| Tubb5 | P69897 | 2 | 2.05E-05 | 2.12E-05 | 3.49E-11 | 4.88E-11 | 0.9892 | 0.1483 | 9.09E-01 |
| Tubb5; Tubb4b | P69897; G3V7C6 | 5 | 1.36E-04 | 1.15E-04 | 5.36E-08 | 5.04E-08 | 1.0560 | 6.1131 | 5.09E-01 |
| Tufm | P85834 | 5 | 1.38E-04 | 1.43E-04 | 1.06E-09 | 5.16E-10 | 0.9631 | 0.0583 | 9.84E-01 |
| Txnrd2 | Q9Z0J5 | 2 | 6.75E-06 | 6.97E-06 | 1.28E-12 | 8.30E-13 | 0.9542 | 0.0297 | 8.15E-01 |
| Uba1 | Q5U300 | 3 | 1.50E-05 | 1.61E-05 | 1.59E-11 | 1.01E-11 | 0.9106 | 0.0920 | 2.03E-01 |
| Ube2l3 | B2RZA9 | 2 | 1.85E-05 | 2.07E-05 | 9.20E-12 | 1.09E-11 | 0.8727 | 0.0336 | 2.71E-01 |
| Ube2n | Q9EQX9 | 2 | 2.10E-05 | 2.37E-05 | 1.27E-11 | 9.23E-12 | 0.8567 | 0.0335 | 4.26E-01 |
| Ugp2 | Q4V8I9 | 3 | 1.97E-05 | 1.83E-05 | 1.94E-11 | 4.84E-11 | 1.0768 | 0.3058 | 8.41E-01 |
| Uncharacterized protein | M0R5J4 | 5 | 2.62E-04 | 3.19E-04 | 8.60E-09 | 6.01E-09 | 0.8497 | 0.0971 | 6.71E-02 |
| Uncharacterized protein | D4A8Y1 | 5 | 4.13E-05 | 4.13E-05 | 8.77E-11 | 3.29E-11 | 0.9819 | 0.0757 | 3.78E-01 |
| Uncharacterized protein | D3ZEK8 | 2 | 1.70E-05 | 1.87E-05 | 5.22E-12 | 4.93E-12 | 0.8116 | 0.0276 | 4.49E-01 |
| Uncharacterized protein | D3Z874 | 5 | 6.32E-05 | 5.89E-05 | 1.10E-10 | 5.08E-11 | 1.0379 | 0.0552 | 8.78E-01 |
| Uncharacterized protein (Fragment) | F1M7L9 | 2 | 2.55E-05 | 2.33E-05 | 2.11E-11 | 4.46E-11 | 1.0879 | 0.1322 | 2.54E-01 |
| Uncharacterized protein (Fragment) | F1LPQ6 | 2 | 1.40E-04 | 1.32E-04 | 8.51E-09 | 7.13E-09 | 1.0889 | 1.1539 | 5.88E-01 |
| Uncharacterized protein (Fragment) | D4A4B0 | 5 | 2.69E-04 | 2.20E-04 | 1.23E-08 | 3.40E-09 | 1.0551 | 0.1269 | 9.71E-01 |
| Uncharacterized protein; Eno3; Enolase; Eno2 | M0R5J4; P15429; D3ZYU0; P07323 | 2 | 2.58E-04 | 3.35E-04 | 8.98E-09 | 2.53E-09 | 0.7485 | 0.2311 | 5.83E-01 |

| | | | | | | | | | |
|---|---|---|---|---|---|---|---|---|---|
| Uncharacterized protein; Enolase | M0R5J4; D3ZYU0 | 4 | 1.62E-04 | 1.99E-04 | 3.12E-09 | 2.74E-09 | 0.7726 | 0.1140 | 5.74E-02 |
| Uncharacterized protein; Uncharacterized protein (Fragment) | D4A8Y1; D4A4B0 | 5 | 6.00E-05 | 6.05E-05 | 1.82E-10 | 1.26E-10 | 0.9903 | 0.0966 | 7.25E-01 |
| Uqcr10 | B2RYX1 | 2 | 1.50E-04 | 1.58E-04 | 8.14E-09 | 1.92E-09 | 0.9523 | 0.5014 | 5.63E-01 |
| Uqcrb | B2RYS2 | 3 | 1.47E-04 | 1.49E-04 | 5.51E-10 | 9.43E-10 | 0.9933 | 0.0894 | 8.14E-01 |
| Uqcrc1 | Q68FY0 | 5 | 4.30E-04 | 4.02E-04 | 7.10E-09 | 8.21E-09 | 1.1087 | 0.0961 | 1.20E-01 |
| Uqcrc2 | P32551 | 5 | 4.55E-04 | 3.99E-04 | 3.42E-09 | 3.23E-09 | 1.1403 | 0.0412 | 1.40E-04 |
| Uqcrfs1 | P20788 | 5 | 3.39E-04 | 3.34E-04 | 2.40E-09 | 3.00E-09 | 1.0718 | 0.0616 | 1.40E-01 |
| Uqcrh | Q5M9I5 | 3 | 6.73E-05 | 7.67E-05 | 5.82E-10 | 7.50E-10 | 0.9958 | 0.1447 | 7.65E-01 |
| Uqcrq | Q7TQ16 | 2 | 1.96E-04 | 1.98E-04 | 1.31E-09 | 1.81E-09 | 1.0052 | 0.2703 | 9.65E-01 |
| Vcl | P85972 | 5 | 1.09E-04 | 7.90E-05 | 1.37E-09 | 2.42E-10 | 1.2609 | 0.1649 | 1.71E-03 |
| Vcp | P46462 | 5 | 2.93E-05 | 2.76E-05 | 1.98E-11 | 7.14E-12 | 0.9111 | 0.0673 | 5.73E-01 |
| Vdac1 | Q9Z2L0 | 5 | 1.76E-04 | 1.83E-04 | 7.40E-10 | 6.33E-10 | 0.9128 | 0.0558 | 8.27E-01 |
| Vdac1; Vdac1 | F1M2D3; Q9Z2L0 | 5 | 4.78E-04 | 4.72E-04 | 6.08E-09 | 2.86E-09 | 1.0216 | 0.0391 | 7.76E-01 |
| Vdac2 | P81155 | 5 | 1.39E-04 | 1.37E-04 | 2.71E-10 | 2.21E-10 | 1.0340 | 0.0419 | 1.88E-01 |
| Vdac3 | A0A0A0MY29 | 5 | 2.72E-04 | 2.18E-04 | 5.37E-09 | 2.44E-09 | 1.1786 | 0.1667 | 1.07E-01 |
| Vim | G3V8C3 | 5 | 3.29E-05 | 3.43E-05 | 1.27E-09 | 9.37E-10 | 0.9407 | 1.3240 | 7.37E-01 |
| Vim; Des | G3V8C3; Q6P725 | 2 | 2.94E-04 | 2.88E-04 | 1.65E-09 | 2.52E-09 | 1.0160 | 0.0506 | 2.02E-01 |
| Ywhae | P62260 | 4 | 5.45E-05 | 5.84E-05 | 1.80E-10 | 1.07E-10 | 0.9179 | 0.0831 | 3.92E-01 |
| Ywhag | P61983 | 4 | 4.39E-05 | 2.95E-05 | 3.78E-10 | 9.04E-11 | 1.3601 | 0.5054 | 4.34E-02 |
| Ywhah | P68511 | 4 | 3.98E-05 | 3.81E-05 | 4.39E-10 | 8.53E-11 | 1.0043 | 0.2956 | 8.12E-01 |
| Ywhah; Ywhaq; Ywhag; Ywhaz; Ywhae | P68511; P68255; P61983; P63102; P62260 | 3 | 1.26E-04 | 1.34E-04 | 3.02E-09 | 1.17E-09 | 0.8689 | 0.1206 | 9.17E-01 |
| Ywhaz | P63102 | 4 | 2.56E-05 | 2.83E-05 | 5.30E-11 | 4.81E-11 | 0.9003 | 0.0933 | 5.99E-01 |
| Zero beta-globin (Fragment) | Q63011 | 2 | 1.69E-03 | 2.33E-03 | 4.04E-07 | 2.03E-06 | 0.7295 | 0.1592 | 5.56E-01 |
| Zero beta-globin (Fragment); Hbb-b1 | Q63011; Q62669 | 5 | 7.57E-04 | 8.39E-04 | 5.72E-08 | 2.53E-07 | 0.9729 | 2.6427 | 7.56E-01 |
